# Pancreatic tumor eradication via selective Pin1 inhibition in cancer-associated fibroblasts and T lymphocytes engagement

**DOI:** 10.1038/s41467-022-31928-7

**Published:** 2022-07-25

**Authors:** Jiaye Liu, Yang Wang, Chunyang Mu, Meng Li, Kewei Li, Shan Li, Wenshuang Wu, Lingyao Du, Xiaoyun Zhang, Chuan Li, Wei Peng, Junyi Shen, Yang Liu, Dujiang Yang, Kaixiang Zhang, Qingyang Ning, Xiaoying Fu, Yu Zeng, Yinyun Ni, Zongguang Zhou, Yi Liu, Yiguo Hu, Xiaofeng Zheng, Tianfu Wen, Zhihui Li, Yong Liu

**Affiliations:** 1 Department of Thyroid and Parathyroid Surgery, West China Hospital, Sichuan University, Chengdu, China; 2 Laboratory of Thyroid and Parathyroid diseases, Frontiers Science Center for Disease-Related Molecular Network, West China Hospital, Sichuan University, Chengdu, China; 3 State Key Laboratory of Biotherapy and Cancer Center, West China Hospital, Sichuan University and Collaborative Innovation Center, Chengdu, China; 4 Respiratory Health Institute, Frontiers Science Center for Disease-related Molecular Network, West China Hospital, Sichuan University, Chengdu, China; 5 Department of Medical Biochemistry and Biophysics, Karolinska Institute, Stockholm, Sweden; 6 Department of Liver Surgery & Liver Transplantation Center, West China Hospital, Sichuan University, Chengdu, China; 7 Guangzhou Institutes of Biomedicine and Health, Chinese Academy of Sciences, Guangzhou, China; 8 Guangzhou Regenerative Medicine and Health Guangdong Laboratory, Bioland Laboratory, Guangzhou, China; 9 Department of Pediatric Department, West China Hospital, Sichuan University, Chengdu, China; 10 Department of Hepatobiliary Surgery, Daping Hospital, Army Medical University, Chongqing, China; 11 Center of Infectious Diseases, West China Hospital, Sichuan University, Chengdu, China; 12 Department of Gastrointestinal Surgery, West China Hospital, Sichuan University, Chengdu, China; 13 Department of Gastroenterology, West China Hospital, Sichuan University, Chengdu, China; 14 Department of Rheumatology and Immunology, Rare Disease Center, West China Hospital, Sichuan University, Chengdu, China; 15 Department of Endocrinology and Metabolism, Center for Diabetes and Metabolism Research, West China Hospital, Sichuan University, Chengdu, China

**Keywords:** Pancreatic cancer, Pancreatic cancer, Pancreatic cancer

## Abstract

Cancer associated fibroblasts (CAFs) support tumors via multiple mechanisms, including maintaining the immunosuppressive tumor microenvironment and limiting infiltration of immune cells. The prolyl isomerase Pin1, whose overexpression in CAFs has not been fully profiled yet, plays critical roles in tumor initiation and progression. To decipher effects of selective Pin1 inhibition in CAFs on pancreatic cancer, here we formulate a DNA-barcoded micellular system (DMS) encapsulating the Pin1 inhibitor AG17724. DMS functionalized with CAF-targeting anti-FAP-α antibodies (antiCAFs-DMS) can selectively inhibit Pin1 in CAFs, leading to efficacious but transient tumor growth inhibition. We further integrate DNA aptamers (AptT), which can engage CD8+ T lymphocytes, to obtain a bispecific antiCAFs-DMS-AptT system. AntiCAFs-DMS-AptT inhibits tumor growth in subcutaneous and orthotopic pancreatic cancer models.

## Abstract (web summary)

Pharmacological inhibition of the prolyl isomerase PIN1, highly expressed in cancer cells and cancer associated fibroblasts (CAF), has been proposed for cancer therapy. Here the authors report the design of a DNA-barcoded micellular system functionalized with antibodies targeting CAFs and a T cell recruiting aptamer to deliver the PIN1 inhibitor AG17724, showing antitumor response in preclinical models of pancreatic cancer.

## Introduction

Cancer immunotherapies, including checkpoint blockade, adoptive cellular therapy, cancer vaccinology, and bispecific T cell engagers, have shown potential for the cure of cancers^1–3^. Nonetheless, only a small subset of the patients within a large cohort can respond favorably to these immunotherapies^4,5^. Accumulative findings have correlated this with the immunosuppressive tumor microenvironment (TME)^6–8^, to which cancer-associated fibroblasts (CAFs) greatly contribute^9–12^. CAFs are heterogeneous stromal cells and are prominent components of the TME in solid tumors and shape the immune ecosystem of TME toward a tolerant and immunosuppressive milieu via multiple mechanisms, including the production of multiple cytokines and chemokines that then mediate the recruitment and functional differentiation of innate and adaptive immune cells^7,13,14^. Furthermore, CAFs can directly interact with tumor-infiltrating immune cells in negative ways, abrogating their function of tumor cell killing^15,16^.

Prolyl isomerase Pin1, which is highly expressed in both cancer cells and CAFs^17^, facilitates multiple cancer-driving pathways by regulating the conformational transformation of phosphorylated Serine/Threonine-Proline motif^18,19^. Pharmacological inhibition of Pin1 has therefore been regarded as a potent anticancer strategy^17^. A few small molecule compounds that inhibit Pin1, such as all-trans retinoic acid, arsenic trioxide, juglone, AG17724, KPT-6566, and sulfopin, have been identified and used or repurposed for investigating the roles of Pin1 in oncogenesis^20–24^. All these compounds, however, when applied in vivo, only a small fraction of injected amount can finally distribute into the tumor.

**Fig. 1:**
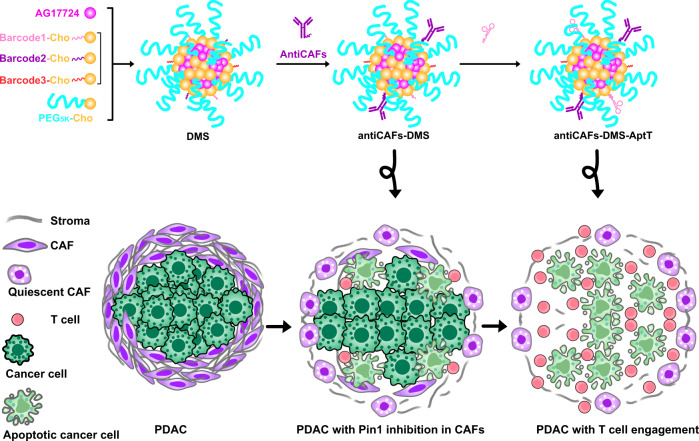
Schematic illustration of antiCAFs-DMS and antiCAFs-DMS-AptT for selective delivery of PIN inhibitor to CAFs and then directing T Lymphocytes to TME of PDAC.

To figure out the exclusive effects of Pin1 in CAFs on tumor progression, one strategy can be about delivering currently available Pin1 inhibitors via customized drug delivery systems (DDSs) to CAFs. DDSs, including these targeting CAFs, have continuously proven their preclinical successes for cellular or even subcellular targeting drug transportation^25–29^. DDSs formulated for the delivery of Pin1 inhibitor to CAFs, however, have not been developed yet. DNA-barcoding on DDSs has recently been emerging as a superior and applicable way to track the drug of nanoparticle that entered each tumor cell^30^, facilitate the discovery of nanoparticles targeting specific tissues and cells^31^, or enable tunable functionalization of biomaterials for immune cell modulation^32,33^. Herein, we report a DNA-barcoded micellular system to deliver the Pin1 inhibitor AG17724 (designated as “DMS”) (Fig. 1). DMS functionalized with antibodies targeting CAFs (antiCAFs-DMS) is customized to specifically deliver AG17724 to CAFs in subcutaneous and orthotopic pancreatic ductal adenocarcinoma (PDAC), thus helping us to understand functions of Pin1 in CAFs on supporting PDAC growth. Furthermore, antiCAFs-DMS is mounted with an immune cell-recruiting DNA aptamer (antiCAFs-DMS-AptT) to engage CD8^+^ T lymphocytes for PDAC eradication.

## Results

### DMS packs AG17724 and displays DNA barcodes for post functionalization

**Fig. 2:**
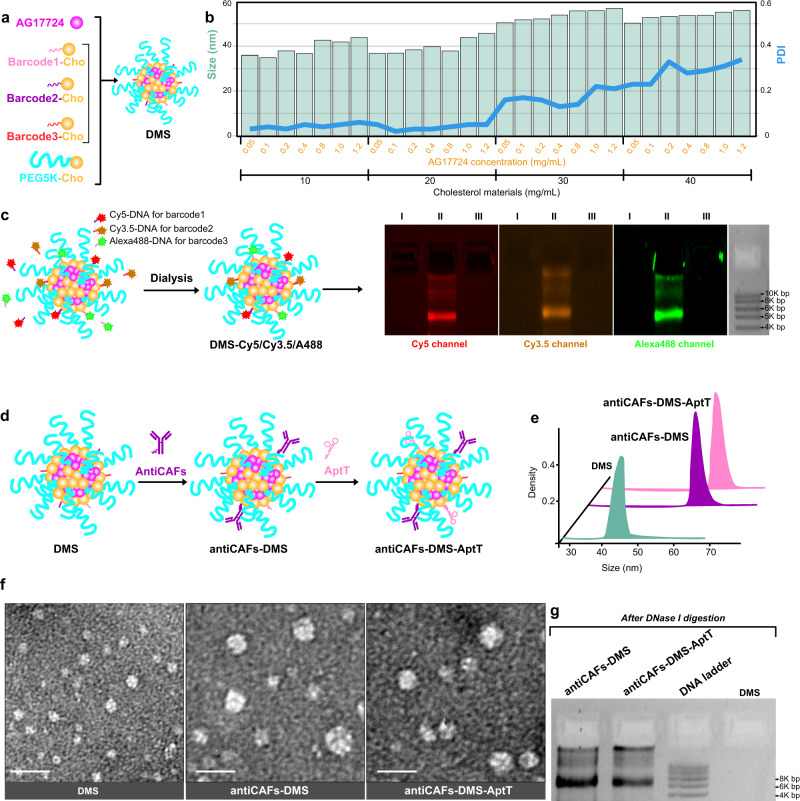
Characterization of formulations. **a** Schematic compositions and self-assembly of DMS. **b** Corresponding sizes and PDIs of DMS prepared from different concentrations of AG17724 and cholesterol materials. Three independent repetitions got similar results. **c** Validation of DNA barcodes on DMS via using fluorescent probe-labeled complimentary DNA oligos. I, DMS; II, DMS-Cy5/Cy3.5/A488; III, DMS incubated with Cy5-scrambled DNA, Cy3.5-scrambled DNA, and A488-scrambled DNA. Three independent repetitions got similar results. **d** Schematic workflow to prepare antiCAFs-DMS and antiCAFs-DMS-AptT from DMS. **e** Hydrodynamic size distributions of DMS, antiCAFs-DMS, and antiCAFs-DMS-AptT. Three independent repetitions got similar results. **f** TEM images of nanoparticles. Scale bars are 100 nm. Three independent repetitions got similar results. **g** Silver staining-based antibody detections after releasing antibodies from the nanoparticles. Three independent repetitions got similar results. Source data are provided as a Source Data file.

We prepared the DMS via self-assembly from PEG_5K_-cholesterol (PEG_5K_-Cho), DNA-barcoded cholesterol (BarcodeX-Cho), and Pin1 inhibitor AG17724 (Fig. 2a). The hydrophobicity of AG17724 drove its encapsulation by the cholesterol core of DMS. The hydrophilic PEG_5K_ polymers surrounding DMS were supposed to stabilize the system^34^. DNA barcodes on the final DMS could facilitate post-functionalization, allowing sequential and reliable attachments of DNA-conjugated ligands. Theoretically, many different barcodes, which correspond to a specific sequence of the DNA, can be used here. We used three (Supplementary Table 1) to show the proof of concept. Besides, all these materials nowadays are commercially available and widely used in biomedical research, which can thus facilitate reproductivity and accelerate translation.

To figure out the optical DMS preparation, the molar ratio of barcode1-Cho/barcode2-Cho/barcode3-Cho/PEG_5K_-Cho was fixed at 1/1/1/7, while serial concentrations of them (from 10 to 40 mg/mL) and AG17724 (from 0.05 to 1.2 mg/mL) were screened with measuring the corresponding size and polydispersity index (PDI) (Fig. 2b). It showed that sizes were always between 30 nm to 60 nm while PDI increased to more than 0.1 once the concentration of cholesterol materials was above 30 mg/mL. This led us to use “20 mg/mL cholesterol materials with 1.2 mg/mL AG17724” to prepare the final DMS, which had AG17724 encapsulation efficiency and loading efficiency at 83.6 ± 1.9% and 45.8 ± 2.4%, respectively. DMS had its size at 43 ± 1.8 nm and PDI at 0.05 ± 0.01. DMS had a negative zeta potential at around −4.3 mV (Supplementary Fig. 1), which should be mainly contributed by DNA barcodes. DMS showed size stabilities with 10% serum at room temperature for 1-week (Supplementary Fig. 2). Less than 10% of loaded AG17724 was released during this one-week evaluation, further demonstrating its stability (Supplementary Fig. 3). This delayed release in vitro can reduce the risk of immediate release of AG17724 from DMS in bloodstream, mitigating off-target effects. After internalizing by targeting cells, the release of AG17724 would be accelerated by the lysosomes.

To check the availability of DNA barcodes on DMS, we used complementary DNA strands attached with different fluorescent probes (Supplementary Table 2), including Cy5, Cy3.5, and Alexa488, to visualize them. After removing excessive probes via dialysis, we ran samples on 2% agarose gel and then imaged the gels under specific channels (Fig. 2c). We can detect all signals from the DMS sample, indicating that all three different barcodes were well presented and accessible even though it is hard to absolutely quantify each barcode. One group of these barcodes was used as coordinates to hybridize DNA-conjugated CAFs-targeting antibodies (antiCAFs) (Supplementary Fig. 4) to get antiCAFs-DMS (Fig. 2d). We used the antibody recognizing the membrane biomarker, fibroblast activation protein alpha (FAP-α), on CAFs as the antiCAFs^35^. Furthermore, aiming to direct immune cells against tumors, we hybridized DNA aptamers (Supplementary Table 3), which were screened to bind CD8^+^ T lymphocytes with high specificity and selectivity^36^, with another group of barcodes on antiCAFs-DMS to get the bispecific antiCAFs-DMS-AptT. Hydrodynamic sizes of both antiCAFs-DMS and antiCAFs-DMS-AptT were similarly around 63 nm (Fig. 2e), which were around 20 nm bigger than DMS. Transmission electron microscopy (TEM) imaging of negatively stained samples further validated size differences among these three preparations (Fig. 2f). We verified the existence of antibodies on both antiCAFs-DMS and antiCAFs-DMS-AptT by gel silver staining after releasing antibody via DNase I digestion (Fig. 2g).

### Antibody-functionalized DMS selectively inhibits Pin1 in CAFs

**Fig. 3:**
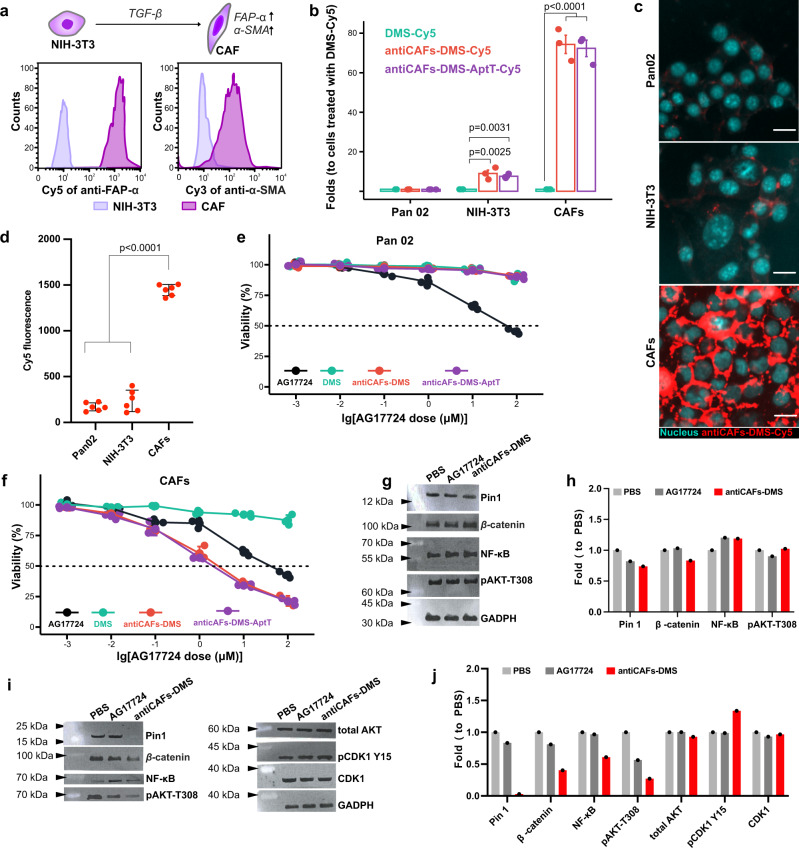
Specific Pin1 inhibition in CAFs achieved by antibody modified DMS in vitro. **a** Establishment and validation of CAFs via high expressions of both FAP-α and α-SMA. **b** Cellular uptake of Cy5-labeled formulations on Pan02 cells, NIH-3T3 cells, and CAFs, measured by flow cytometry (*n* = 3 biologically independent experiments; error bars, mean ± SD). **c** Cellular uptake of Cy5-labeled antiCAFs-DMS on cells, imaged by fluorescent microscopy. Scale bars are 20 μm. Three independent repetitions got similar results. **d** Cellular uptake of Cy5-labeled antiCAFs-DMS on cells, quantified by flow cytometry (*n* = 6 biologically independent samples; error bars, mean ± SD). Cytotoxicity on Pan02 cells (**e**) and CAFs (**f**) (*n* = 3 biologically independent experiments; error bars, mean ± SD). **g** Western blotting detection of Pin1 and relative proteins from Pan02 cells after treating with PBS, AG17724 (0.5 μM), or antiCAFs-DMS (corresponds to 0.5 μM of AG17724) for 24 hours. **h** Quantitative analysis of the western blotting results from Pan02 cells (Three independent repetitions got similar results.). **i** Western blotting detection of Pin1 and relative proteins from CAFs treated with PBS, AG17724, or antiCAFs-DMS after treating with PBS, AG17724 (0.5 μM) or antiCAFs-DMS (corresponds to 0.5 μM of AG17724) for 24 hours. **j** Quantitative analysis of the western blotting results from CAFs (Three independent repetitions got similar results.). One-way analysis of variance followed by Turkey posttests. Source data are provided as a Source Data file.

We cultured CAFs by inducing the differentiation of normal fibroblasts (NIH-3T3 cells) with transforming growth factor beta (TGF-β)^37^, and we validated its reliability via showing drastically increased expressions of both FAP-α and alpha-smooth muscle actin (α-SMA) (Fig. 3a). Flow cytometry-based cellular uptake analysis showed the high selectivity of both antiCAFs-DMS and antiCAFs-DMS-AptT on CAFs rather than pancreatic cancer cells (Pan02), displaying around 70-fold differences (Fig. 3b). Further qualitative (Fig. 3c) and quantitative (Fig. 3d) analysis on the uptake of antiCAFs-DMS among Pan02 cells, NIH-3T3 cells and CAFs demonstrated the similar differences.

We then studied the cytotoxicity of all formulations on Pan02 cells (Fig. 3e) and CAFs (Fig. 3f). Owe to its poor cell permeability^38–40^, compound AG17724 itself showed high IC_50_ at around 50 μM for both cells. IC_50_ of DMS exceeded our experimental range for both cells, which was due to its low cell uptake efficiency. Intriguingly, antiCAFs-DMS and antiCAFs-DMS-AptT showed their IC_50_ on CAFs at around 1.2 μM, whereas their cytotoxicity on Pan02 cells was much lower. This indicated that attached FAP-α antibodies can firstly help antiCAFs-DMS and antiCAFs-DMS-AptT targeting CAFs, they then also can facilitate the internalization of the whole system by cells, achieving a specific and efficient delivery of Pin1 inhibitor into CAFs.

To study the selective inhibition of Pin1 catalytic activity by AG17724, we performed PPIase assay and compared AG17724 with other Pin1 inhibitors including all-trans retinoic acid (ATRA) and Juglone. AG17724 showed a more potent Pin1 inhibition capacity (Ki at 0.03 μM) than either ATRA (Ki at 1.99 μM) or Juglone (Ki above 10 μM) (Supplementary Fig. 5a). At the cellular level, we compared the effects of AG17724 or antiCAFs-DMS on the proliferation of Pin1 knockdown CAFs (Supplementary Fig. 5b) and wild-type CAFs, showing that Pin1-knockdown CAFs were more resistant to AG17724 (Supplementary Fig. 5c) or antiCAFs-DMS (Supplementary Fig. 5d) than wild-type CAFs. To further support the thesis that AG17724 targets Pin1 in cells, we next carried out RT-qPCR to examine the effect of AG17724, antiCAFs-DMS, ATRA, or Pin1 knockdown on an abundance of a set of oncogenes and tumor suppressors whose expression is regulated by Pin1^41,42^. On wild-type CAFs, it shows that AG17724 itself could not change the abundances of these transcripts, which could be again due to its poor cell permeability^40^. The treatment of antiCAFs-DMS showed a similar capacity as ATRA or shPin1 to affect transcriptions of these selected genes (Supplementary Fig. 5e), indicating that AG17724 delivered by antiCAFs-DMS indeed inhibited Pin1.

To see if Pin1 could be inhibited, cells were treated with 0.5 μM of AG17724 for 4 hours and relative proteins were analyzed. As a critical modifier of multiple cancer-related pathways, Pin1 has been revealed to activate more than 55 proteins, including β-catenin, NF-κB, and AKT. We herein checked them, and we observed that, for pancreatic cancer cells, Pin1 and these three relative proteins were at similar levels no matter whether they were treated with phosphate-buffered saline (PBS), AG17724, or antiCAFs-DMS (Fig. 3g, h). For CAFs, antiCAFs-DMS significantly inhibited Pin1, and also efficiently inhibited β-catenin, NF-κB, and AKT to different extents. AG17724 itself showed the capacity to inhibit AKT, however, it didn’t block Pin1, β-catenin, and NF-κB (Fig. 3i, j). These results showed similar patterns to the cellular uptake part and cytotoxicity part aforementioned, further indicating that antiCAFs-DMS was potent for selective Pin1 inhibition in CAFs.

### Pin1 inhibitions in CAFs reduce the invasion and growth of pancreatic cancer spheroids

**Fig. 4:**
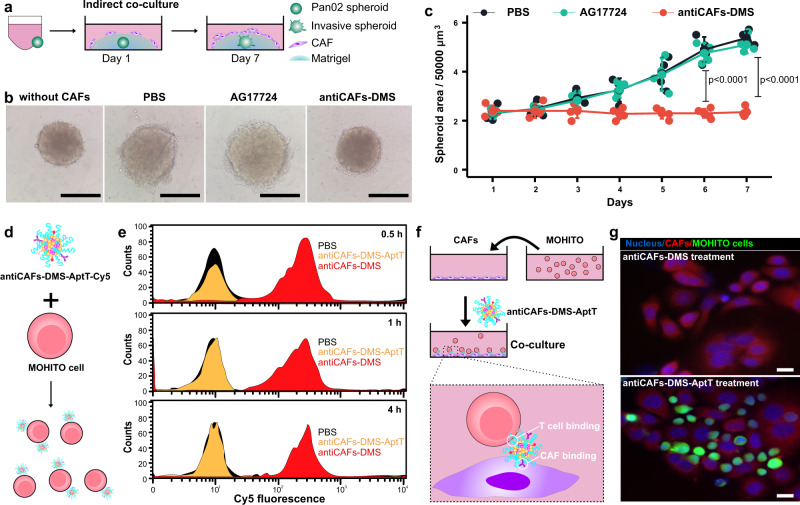
Cancer cells and T-cell engagement via bispecific antiCAFs-DMS-AptT in 3D and 2D models. **a** Schematic workflow of Pan02 spheroids and CAFs indirect co-culture. **b** Representative morphologic images of Pan02 spheroids at the end of the indirect co-culture. Scale bars are 100 μm. **c** Dynamic growth curves of Pan02 spheroids during the indirect co-culture (*n* = 5 biologically independent spheroids; Error bars, mean ± SD). Two-tailed Student’s *t* tests. **d** Schematic workflow of the incubation of MOHITO cells with Cy5-labeled antiCAFs-DMS-AptT. **e** Quantitative analysis of the binding of Cy5-labeled antiCAFs-DMS or antiCAFs-DMS-AptT to MOHITO cells via flow cytometry (Three independent repetitions got similar results.). **f** Schematic illustration of the direct co-culture of CAFs and MOHITO cells. **g** Representative microscopic images of the co-culture experiment. Three independent repetitions got similar results. Scale bars are 10 μm. Source data are provided as a Source Data file.

CAFs have been revealed to enhance the growths of different tumors^11^. For pancreatic cancer, especially, the molecular crosstalk between cancer cells and CAFs facilitates tumor invasion and growth^17,43^. To determine whether selective Pin1 inhibition in CAFs affects their ability to act on pancreatic cancer cells, we conducted the indirect co-culture of pre-formed pancreatic cancer spheroids with CAFs (Fig. 4a). Unlike spheroids cultured under the same condition but without adding CAFs, spheroids with indirect CAFs co-culture showed invasive cells at their surfaces, confirming that CAFs can remotely act on cancer cells, which could be via humoral factors (Fig. 4b). AG17724 (0.5 μM) failed to inhibit invading signs around spheroids in matrigel. In contrast, the antiCAFs-DMS treatment stopped the invasion of spheroids, displaying shrinking margins. The dynamic volume records of these spheroids further showed the inhibitive effect of antiCAFs-DMS on their growth (Fig. 4c). Spheroids with antiCAFs-DMS treatment showed the lowest ratio of live cells to dead cells (Supplementary Fig. 6). These results together firstly highlighted that Pin1 is important for CAFs to promote growth and invasion of pancreatic cancer spheroids. Secondly, we showed that Pin1 inhibitor AG17724 delivery, via antiCAFs-DMS, into CAFs might be an alternative and potent way for pancreatic cancer treatment.

### Aptamer modification on antiCAFs-DMS bridges T Lymphocytes to CAFs

Solid tumors, PDAC especially, often show their resistance to immunotherapy^44^. Apart from their own immunosuppressive TME, in which cancer cells debilitate the antitumor immunity of resident immune cells via secretion or presentation of inhibiting molecules and collaboration with CAFs, dense architectures, largely contributed by CAFs, raise physical obstacles for active peripheral immune cells to infiltrate^45–47^. With the interest in bridging active peripheral T lymphocytes to PDAC, we prepared antiCAFs-DMS-AptT as mentioned above. After incubating murine CD8^+^ T cells with Cy5-labeled systems (Fig. 4d), flow cytometry analysis showed that cells treated with antiCAFs-DMS-AptT had much higher fluorescent intensity than cells treated with antiCAFs-DMS, confirming that this aptamer decoration can efficiently lead the system to stick to T cells (Fig. 4e). There was no big difference, in term of Cy5 signal on T cells, among time points of 0.5, 1 and 4 hours, indicating that both binding and saturation were fast.

Since antiCAFs-DMS-AptT contains antibodies for targeting CAFs but also aptamers for the binding of CD8^+^ T cells, it ideally could function as bispecific modules, which have already shown promising potentials in immunotherapy^48–50^, to bridge T cells to CAFs. To validate this, we conducted the co-culture of CAFs and T cells in vitro (Fig. 4f). We observed that T cells were barely located on CAFs under antiCAFs-DMS treatment. AntiCAFs-DMS-AptT treatment, nevertheless, showed its effect to bridge T cells to CAFs (Fig. 4g). These results indicated that, apart from the delivery of Pin1 to CAFs, antiCAFs-DMS-AptT potentially can also direct T lymphocytes to CAFs, resulting in more immune cells in TME. Being different from bispecific antibodies, aptamers, or nanoparticles, which usually still can’t overcome physical obstacles of PDAC, antiCAFs-DMS-AptT can render solid tumors accessible for T lymphocytes. We also isolated murine primary CD8^+^ T cells to perform ex vivo co-culture experiments with CAFs and luciferase-expressing cancer cells. Our results showed that antiCAFs-DMS-AptT can significantly induce CD8^+^ T cell-mediated lysis on both CAFs and cancer cells (Supplementary Fig. 7a, b), indicating that antiCAFs-DMS-AptT indeed worked as a bispecific system to bridge CD8^+^ T cells to CAFs and exert cytotoxic effects on pancreatic cancer cells nearby.

The aptamer modified on antiCAF-DMS-AptT was previously discovered for the traceless isolation of pure CD8^+^ T cells at a high yield^36^. AntiCAFs-DMS-AptT thus is designed to bind CD8^+^ T cells but not get internalized by them. To prove this, we used DNase I treatment to remove antiCAFs-DMS-AptT-Cy5 staying on the surface of CD8^+^ T cells and measured the Cy5 signal before and after (Supplementary Fig. 8), showing that DNase I can almost completely decrease Cy5 signal from high level to the level of PBS treatment. This indicated that antiCAFs-DMS-AptT mostly stayed on the cell surface of CD8^+^ T cells. Besides, we investigated the viability of CD8^+^ T cells after incubation with AG17724, DMS, antiCAFs-DMS, or antiCAFs-DMS-AptT for 48 hours. It showed that, after being encapsulated into DMS, the toxicity of AG17724 to T cells decreased (Supplementary Fig. 9a). We further tested CD8^+^ T cell functions after treating them with 0.5-μM AG17724 corresponding antiCAFs-DMS-AptT, via measuring T cell expansion, IFN-γ, IL-2, and TNF. Our results (Supplementary Fig. 9b–e) displayed that antiCAFs-DMS-AptT did not affect the functions of T cells in these four aspects. We can attribute these to the very low uptake of DMS systems by T cells.

### Biodistribution and effects from Pin1 inhibition in CAFs and T cells engagement

**Fig. 5:**
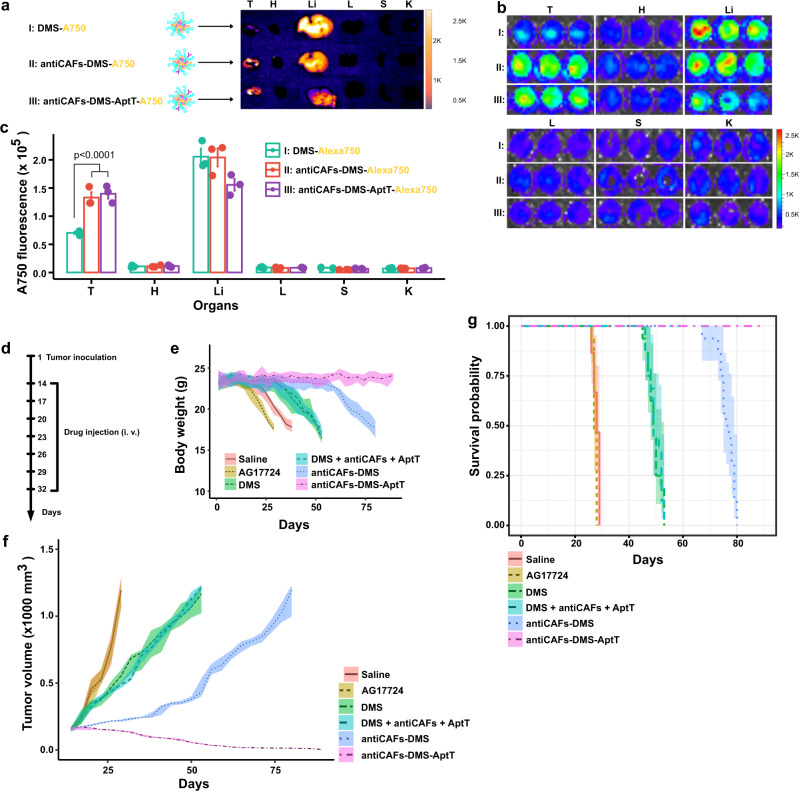
Biodistribution and therapeutic effects. **a** Representative ex vivo imaging of tumors and organs (T tumor, H heart, Li liver, L lung, S spleen, K kidney) from mice intravenously injected with Alexa750-labeled DMS, antiCAFs-DMS, and antiCAFs-DMS-AptT. **b** Representative imaging of homogenates prepared from tumors or organs (T tumor, H heart, Li liver, L lung, S spleen, K kidney) from mice intravenously injected with Alexa750-labeled DMS, antiCAFs-DMS, and antiCAFs-DMS-AptT. **c** Quantitative Alexa750 fluorescent intensity analysis of homogenates prepared from tumors or organs (T tumor, H heart, Li liver, L lung, S spleen, K kidney) (*n* = 3 biologically independent animals; error bars, mean ± SD). One-way analysis of variance followed by Turkey posttests. **d** treatment schedule. **e** Body weight records of tumor-bearing mice receiving different treatments (10 mice per group). **f** Tumor volume growth curves (10 mice per group). **g** Kaplan–Meier tumor-inoculated mouse survival curves (10 mice per group). Source data are provided as a Source Data file.

To assess if our delivery systems could successfully distribute into tumors in vivo, we labeled them with Alexa750 and then intravenously administrated them to mice bearing subcutaneous PDAC. After 4 hours, we can see that livers were the main bio-distributing organs of all systems (Fig. 5a). Very low Alexa750 signals were detected on hearts, lungs, spleens, and kidneys. For tumors, it showed that both antiCAFs-DMS and antiCAFs-DMS-AptT can accumulate to tumors much more efficiently than DMS. To make it more precise and quantitative, we prepared homogenates from these collected organs or tumors, and then imaged them and performed quantitative analysis (Fig. 5b, c). It showed the same trends as organ imaging, quantitively presenting that, compared with DMS, antibody decorations improved its tumor accumulation by around two folds.

We then evaluated their antitumor effects in vivo by following our treatment schedule (Fig. 5d). Body weight changes were recorded. Compared to control mice (saline), AG17724 injection (10 mg/kg) accelerated weight loss, proving that the compound itself caused toxicity to mice. All treatments with nano-formulated AG17724 slowed down the loss of body weight to different extents. AntiCAFs-DMS-AptT treatment showed its capacity to stabilize body weight during the whole experimental period (Fig. 5e). On tumor growth, we didn’t see any effect of AG17724 itself. Both DMS and the mixture of DMS, antibody, and aptamers (DMS+antiCAFs+AptT) administrations slightly delayed the tumor growth. AntiCAFs-DMS stabilized tumors for around 40 days, but it failed to inhibit their growth afterward. AntiCAFs-DMS-AptT showed its promising antitumor capacity, almost eradicating the established tumors (Fig. 5f). Accordingly, the survival time of mice also ranked antiCAFs-DMS-AptT as the best antitumor formulation among our treatments (Fig. 5g).

### CAFs and T cells quantification in tumor tissues

**Fig. 6:**
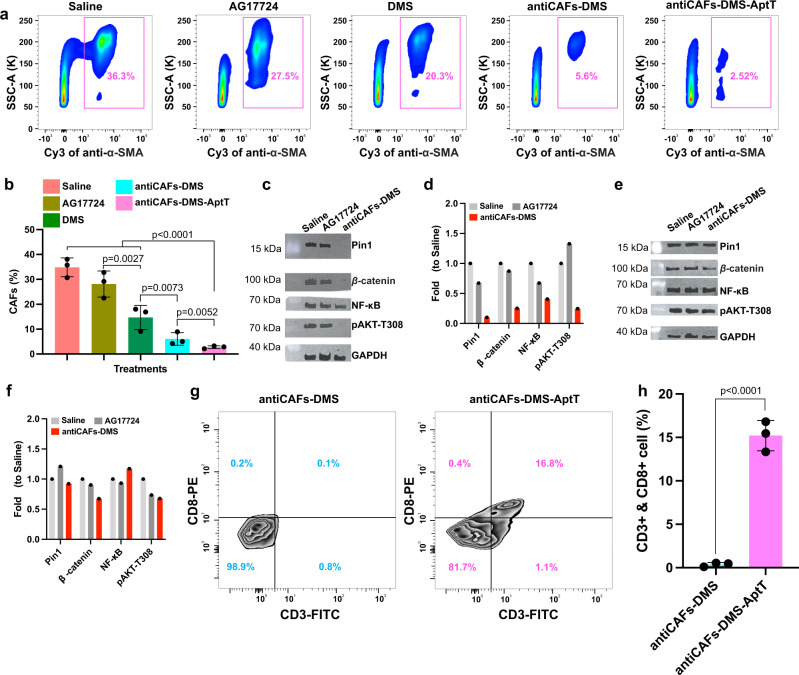
Tumor tissue analysis on CAFs, Pin1, and T lymphocytes. **a**, **b** Quantitative analysis, via cell sorting and counting, of CAFs in tumors from mice receiving different treatments (*n* = 3 biologically independent animals; error bars, mean ± SD). One way analysis of variance followed by Turkey posttests. **c** Western blotting detection of Pin1 and relative proteins in sorted CAFs from tumors collected from mice treated with saline, AG17724 or antiCAFs-DMS. **d** Quantitative analysis of the western blotting results of sorted CAFs (Three independent repetitions got similar results.). **e** Western blotting detection of Pin1 and relative proteins in non-CAFs cells from tumors collected from mice treated with saline, AG17724, or antiCAFs-DMS. **f** Quantitative analysis of the western blotting results of non-CAFs cells (Three independent repetitions got similar results.). **g**, **h** Quantitative analysis, via cell sorting and counting, of CD8^+^ T lymphocytes in tumors from mice treated with antiCAFs-DMS or antiCAFs-DMS-AptT (*n* = 3 biologically independent animals; error bars, mean ± SD). Two-tailed Student’s *t* tests. Source data are provided as a Source Data file.

To check if our CAFs-targeting Pin1 inhibitor delivery systems worked, as expected, to block Pin1 in tumors during the treatment, we took tumors on the 25th day for fluorescence-activated cell sorting analysis. CAFs counting showed us that percentages of CAFs inside tumors were reduced from 36.3% to 20.3%, 5.6%, and 2.5% by DMS, antiCAFs-DMS, and antiCAFs-DMS-AptT, respectively (Fig. 6a, b). This indicated that DMS functionalized with CAFs-targeting antibody can improve its specificity, resulting in more CAFs depletion. Lipid droplet accumulation assay with isolated CAFs showed that antiCAFs-DMS-AptT could significantly increase the number of droplets in CAFs (Supplementary Fig. 10a, b), meaning that efficient Pin1 inhibition can lead CAFs to be quiescent. Cytokine assay showed that Pin1 inhibition from antiCAFs-DMS-AptT treatment inhibited CAFs to secrete a wide range of cytokines, whereas cells treated with AG17724 had similar profiles as control (Supplementary Fig. 10c). High expressions of cytokines, including IL-6 and IL-8, have been regarded as indicators of inflammatory CAFs. We saw their decreased expressions after antiCAFs-DMS-AptT treatment, telling us that the treatment didn’t really induce myofibroblastic CAFs to be inflammatory CAFs.

Pin1 and related proteins in these sorted cells were analyzed via western blots. For CAFs in tumors, Pin1 was significantly inhibited by antiCAFs-DMS treatment whereas AG17724 didn’t display its effect. Besides, β-catenin, NF-κB, and AKT, whose activations are partially controlled by Pin1, were also inhibited by antiCAFs-DMS (Fig. 6c, d). For sorted pancreatic cancer cells from the tumors, we didn’t see an amount of changes in these proteins (Fig. 6e, f). Since our delivery systems contained DNA barcodes, it facilitated us to perform qPCR to quantify the number of DNA barcodes distributed inside CAFs and non-CAFs cells. We detected around 22 times more DNA barcodes in CAFs than in non-CAFs cells (Supplementary Fig. 11). These results together directly further confirmed that antiCAFs-DMS had its cellular selectivity in vivo towards CAFs. Apart from Pin1 inhibition in CAFs, antiCAFs-DMS-AptT treatment, which almost eradicated the established tumors, was also supposed to function as a bispecific CD8^+^ T cells engager. We proved this by comparing CD8^+^ T cell populations inside tumors treated with antiCAFs-DMS or antiCAFs-DMS-AptT. We detected around 16% more intratumor CD8^+^ T cells (16.8%) under antiCAFs-DMS-AptT treatment than antiCAFs-DMS treatment (0.1%) (Fig. 6g, h). These CD8^+^ T cells were further profiled to be active rather than exhausted (Supplementary Fig. 12). This confirmed our expectation, and also told us that the combination of Pin1 inhibition in CAFs and CD8^+^ T lymphocytes engaging could be a potent way to render PDAC eradicable.

### Response of orthotopic murine pancreatic cancer to antiCAFs-DMS-AptT

**Fig. 7:**
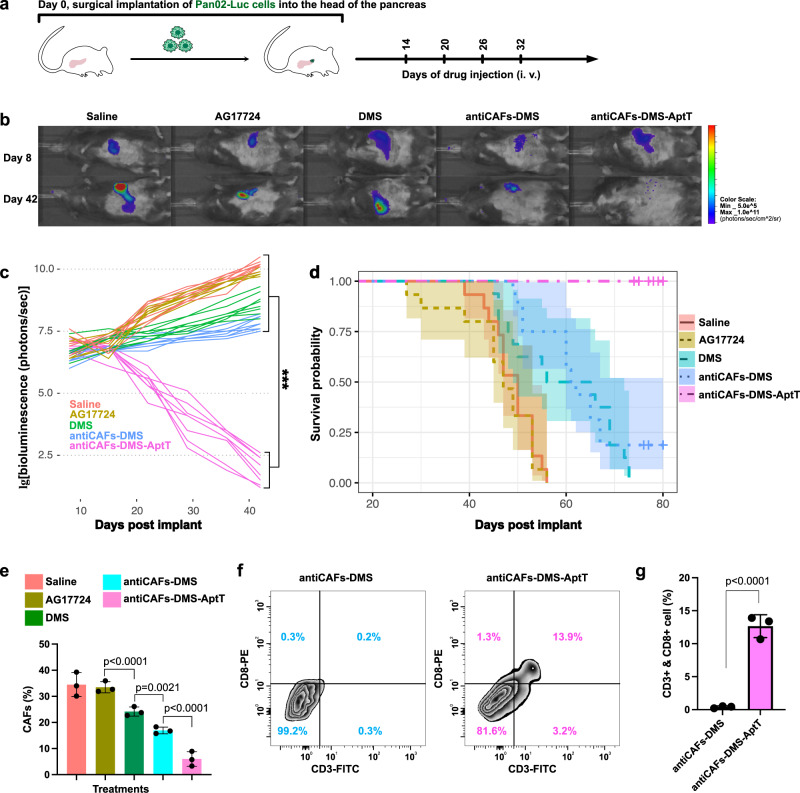
Treatment effects of antiCAFs-DMS-AptT on orthotopic pancreatic cancer model. **a** Treatment schedule of mice bearing orthotopic pancreatic cancer. **b** Representative bioluminescent images of mice treated with different formulations at day 8 and day 42 post cancer establishment (six mice per group). **c** Tumor development of each mouse, quantified by bioluminescence signal (six mice per group). Two-way ANOVA. **d** Kaplan–Meier survival curves of mice (six mice per group). **e** Quantitative analysis, via cell sorting and counting, of CAFs in tumors from mice receiving different treatments (*n* = 3 biologically independent animals; error bars, mean ± SD). **f**, **g** Quantitative analysis of CD8^+^ T lymphocytes in tumors from mice treated with antiCAFs-DMS or antiCAFs-DMS-AptT (*n* = 3 biologically independent animals; error bars, mean ± SD). Two-tailed Student’s *t* tests. Source data are provided as a Source Data file.

We further established orthotopic PDAC to assess if antiCAFs-DMS-AptT treatments eradicate cancer cells in the pancreas. Luciferase-expressing Pan02 cells (Pan02-Luc) we used here helped us, via direct bioluminescent imaging, monitor the cancer developments. Mice received their first treatment on the 14th-day post cancer cell implant (Fig. 7a). Dose administration of each formulation corresponded to 10 mg/kg AG17724. It showed that, after four rounds of treatments, mice of antiCAFs-DMS-AptT group had almost no bioluminescent signals from cancer cells (Fig. 7b), indicating the potent anti-PDAC efficacy of antiCAFs-DMS-AptT on orthotopic pancreatic cancer model. The empty antiCAFs-DMS-AptT (without AG17724 encapsulation) showed no inhibiting effects on tumor progression (Supplementary Fig. 13). This could be attributed to the inherent tumor heterogeneity and highly desmoplastic and immunosuppressive TME of pancreatic cancer, which limits T cell infiltration. Although DMS or antiCAFs-DMS also showed their capacity to slightly slow down the growth rate of cancer cells in pancreas, mice treated with them experienced increasing tumor burdens (Fig. 7c). Correspondingly, antiCAFs-DMA-AptT significantly prolonged the survival time of mice, and the survival rate was 100% during our 80-day investigation (Fig. 7d). Cell population analysis of orthotopic tumors indicated that antiCAFs-DMS-AptT treatment resulted in not only CAFs depletion (Fig. 7e) but also the increase of CD8^+^ T cells (Fig. 7f, g) in tumors.

Since biodistribution results showed that a major fraction of antiCAFs-DMS-AptT distributed into the liver, we assessed if it would affect hepatic functions via measuring glutamic-pyruvate transaminase (GPT), glutamic-oxaloacetic transaminase 1 (GOT1), total protein (TP), albumin (ALB), total bilirubin (TBIL) and alkaline phosphatase (ALP) at the 40^th^ day of treatment schedule. Even though the values of six indicators fluctuated by AG17724 or antiCAFs-DMS-AptT treatment, they were within the normal range (Supplementary Fig. 14). This indicated that neither free AG17724 nor antiCAFs-DMS-AptT elicited significant liver toxicity, representing that antiCAFs-DMS-AptT is biocompatible during the treatment.

## Discussion

Due to the critical roles of Pin1 in tumor initiation and progression, small molecules for inhibiting Pin1 have continuously been screened or developed. However, these inhibitors can lose the pharmacological activity they had in vitro once administrated and diluted in vivo. Besides, a few available drugs have been repurposed for Pin1 inhibition in tumors, which can generally inhibit Pin1 but can’t restrict the inhibition within a specific cell population of the tumor^17,20–24^. The DNA-barcoded micellular systems we are developing in this work prove the feasibility to regulate Pin1 in the cell population of interest. This will then give insights into cell-level antitumor targeting therapy rather than at the whole tumor tissue level.

Densely structured solid tumors are usually resistant to current therapies, which are highly contributed by CAFs^14,47^. Recent literature showed that overexpressed Pin1 displays both in cancer cells and CAFs and aggravates PDAC^17^. How much Pin1 in CAFs contributes to tumor progression, however, is unknown since the lack of drugs exclusively blocking Pin1 in CAFs. We find that Pin1 inhibitor AG17724 can be targeted into CAFs, both in vitro and in vivo, via delivering it by antiCAFs-DMS. Functionally, antiCAFs-DMS selectively and drastically inhibited Pin1 in CAFs, resulting in higher cytotoxicity on CAFs than pancreatic cancer cells. AG17724 itself didn’t show any antitumor therapeutic effect in vivo, which might be related to the poor cell permeability of the compound^38–40^. Intriguingly, we find that effective Pin1 inhibition in CAFs, via antiCAFs-DMS, can slow down the growth of both subcutaneous and orthotopic pancreatic PDAC. However, it is not a potent way to stop tumors since they grew fast once we halted drug injections.

Immunotherapies could be combined with chemotherapy to get better antitumor efficacy^51^, which inspired us to prepare our bispecific antiCAFs-DMS-AptT. Artificial bispecific modules, including bispecific antibodies, bispecific aptamers, and bispecific nanoparticles, are emerging as potent antitumor drugs via directing immune cells of the host against cancer cells^48–50^. The highly desmoplastic TME of certain types of solid tumors, however, can limit their ability to bring immune cells into tumor tissue^17^. We find that antiCAFs-DMS-AptT can eradicate the established tumors. We speculate that Pin1 inhibition in CAFs by antiCAFs-DMS-AptT firstly can disrupt the desmoplastic and immunosuppressive TME, leading to an accessible TME for immune cell infiltration. In the beginning, anti-CAFs-DMS-AptT might not be able to bring CD8^+^ T lymphocytes into pancreatic tumor tissues, nevertheless, antiCAFs-DMS-AptT can still deliver AG17724 into CAFs and thus disrupt the immunosuppressive TME of pancreatic cancer, rendering it “reachable” and “reactive” by immune cells. Recent papers have concluded that, on PDAC, Pin1 inhibition in CAFs via chemical compounds can change the highly desmoplastic and immunosuppressive TME of PDAC, making PDAC eradicable by immunotherapy^17^. During the next treatment stage, anti-CAFs-DMS-AptT then can redirect CD8^+^ T lymphocytes into pancreatic tumor tissue, resulting in the eradication of cancer cells.

In summary, we demonstrated antibody-functionalized DNA-barcoded micellular systems by which selective Pin1 inhibition in CAFs can be achieved. This renders pancreatic tumors eradicable by cytotoxic T lymphocyte engagement. DNA barcodes on these micellular systems offer an easy-to-change way to customize the targeting demand, opening the way for Pin1 inhibition in certain cell populations of the tumor and thus uncovering mechanistic details on how Pin1 supports tumor initiation and progression.

## Methods

### Ethical regulation

All animal experimental procedures were approved by the Animal Experimentation Ethics Committee of Sichuan University (Chengdu, China).

### Reagents, cells, and mice

AG17724, absolute ethanol, chloroform, and dimethyl sulfoxide were purchased from Sigma-Aldrich. DNA barcodes-cholesterol conjugates (Barcode1-Cho: GTGATGGAGATTTATTCTCTT-Cho; Barcode2-Cho: AAAAGAATGATGATAATCATG-Cho; Barcode3-Cho: AGTACTGTGCTAAGATGGTGT-Cho), DNA-florescent conjugates (AAGAGAATAAATCTCCATCAC-Cy5; CATGATTATCATCATTCTTTT-Cy3.5; ACACCATCTTAGCACAGTACT-Alexa488), DBCO-modified DNA oligonucleotides (AAGAGAATAAATCTCCATCAC-DBCO), primers of Barcode1-Cho (Forward: GTGATGGAGATTTAT; Reverse: AAGAGAATAAATCTC) and DNA aptamers (AAGAGAATAAATCTCCATCACCCAGAGTGACGCAGCAACAGAGGTGTAGAAGTACCGTGAACAAGCTTGAAATTGTCTCTGACAGAGGTGGACACGGTGGCTTTTAGT) were synthesized by Integrated DNA Technologies. PEG_5K_-Cho was purchased from Nanosoft Polymers (Winston-Salem, USA). Dulbecco’s Modified Eagle Medium (DMEM, high glucose), fetal bovine serum (FBS), penicillin-streptomycin, Trypsin-EDTA (0.25%), and phosphate-buffered saline (PBS) were purchased from Thermo Fisher SCIENTIFIC. Anti-FAP-α antibody (MAB9727-100, 0.25 µg/mL as working concentration.) and anti-phospho-CDC2/CDK1 (Y15) antibody (AF888-SP, 0.2 µg/mL as working concentration.) were purchased from R&D SYSTEMS. Anti-α-SMA antibody (ab5694, 1 µg/mL as working concentration.), anti-Pin1 antibody (EPR18546-317, 1/2000 as working dilution.), anti-β-catenin antibody (IGX4794R-3, 1 µg/mL as working concentration.), anti-NF-κB p65 antibody (ab207297, 1 µg/mL as working concentration.), anti-AKT1 (phospho T308) antibody (ab278565, 0.1 µg/mL as working concentration.), anti-GAPDH (6C5) antibody (ab8245, 1/2000 as working concentration.), a Cy3 conjugation kit for antibody labeling (ab188287) and a Cy5 conjugation kit for antibody labeling (ab188288) were purchased from abcam. Anti-phospho-Akt (Thr308) antibody (05-802 R, 1/1500 as working dilution.) was purchase from Sigma-Aldrich. HRP-conjugated goat anti-mouse IgG (H + L) cross-adsorbed secondary antibody (G-21040, 1/10000 as working dilution.), FITC-labeled mouse anti-CD3 (17A2) antibody (11-0032-82, 1 µg/mL as working concentration.), PE-labeled mouse anti-CD8 antibody (12-0081-82, 1 µg/mL as working concentration.) and anti-CDK1 (A17) antibody (33-1800, 1 µg/mL as working concentration.) were purchased from Thermo Fisher SCIENTIFIC. Brilliant Violet 510™ anti-mouse CD45 antibody (103137, 1 µg/mL as working concentration.) was purchased from BioLegend. Pan02 cell line (CRL-2553) and NIH-3T3 cell line (CRL-1658) were purchased from ATCC (USA). Pan02-Luc cell line was purchased from Labcorp. Immortalized mouse CD4^+^ CD8^+^ T cell line (MOHITO) (T0131) were purchased from abm. 6-week-old female C57BL/6 mice were purchased from Chengdu Dashuo Biological Institute (Chengdu, China).

### Preparation and characterization of nano-formulations

We used the thin-film hydration method to prepare DMS. In 100-mL round-bottomed flasks, series amounts (as indicated in Fig. 1b) of barcode1-cho, barcode2-cho, barcode3-cho, PEG_5K_-Cho (the molar ratio of barcode1-cho/barcode2-cho/ barcode3-cho/PEG_5K_-Cho was fixed at 1/1/1/7) and AG17724 were dissolved in organic solvent containing 10 mL of absolute ethanol and 1 mL for 1 hour under 37 °C. To obtain thin films, under vacuum, the solvent was removed using rotary evaporation. The flasks were further dried in vacuum drier overnight. The thin films were hydrated in PBS for 0.5 hour in a 37 °C water bath. Collected solutions were sonicated by a probe sonicator at 80 W for 75 seconds. The last step was to filter samples through sterile polyethersulfone membranes with pore size at 0.22 μm (Sigma-Aldrich, USA).

To prepare antiCAFs-DMS, DMS was incubated with DNA-antibody conjugate, whose molar amount was 10-fold excessive than barocode1 on DMS, in PBS at 37 °C overnight. The sample was centrifugated at 2 K × *g* for 5 minutes, excessive DNA-antibody conjugates were in the supernatant and were removed.

To prepare antiCAFs-DMS-AptT, DMS was incubated with DNA-antibody conjugate (10-fold excessive than barocode1 on DMS) and DNA apatamers (10-fold excessive than barocode2 on DMS) in PBS at 37 °C overnight. The sample was centrifugated at 2 K × *g* for 5 minutes, excessive DNA-antibody conjugates and DNA aptamers were in the supernatant and were then removed.

Particles sizes, PDI, and zeta potentials were measured via Malvern Zetasizer Nano ZS90 instrument (Malvern Instruments Ltd., UK).

### Drug release assay

In vitro, AG17724 release study was performed using a dialysis method at 37 °C for 1 week. PBS containing 0.1% (v/v) Tween 80 was used as the release buffer. Nano-formulations were placed into dialysis tubes (MWCO = 8000 Da) and tightly sealed. Then the dialysis tubes were placed into 40 mL of release buffer and were incubated under 37 °C with gentle oscillating at 50 rpm. At specific time points, 1 mL of samples were taken, then centrifuged for 0.5 hour. AG17724 released was quantified in the supernatant by HPLC. 100 μL of release medium was taken out and replaced with equal volume of fresh release buffer. Then the samples were diluted with dimethyl sulfoxide and the concentrations of AG17724 were determined at the wavelength λ = 225 nm by HPLC.

### DNA-antibody conjugation

We used SiteClick™ Antibody Azido Modification Kit (Invitrogen, USA) to conjugate DNA oligos to the Fc region of IgG antibody. Mouse anti-FAP-α antibody was concentrated and buffer exchanged to 200 μg in 50 μL antibody preparation buffer (Component A). We then modified the carbohydrate domain of the antibody by adding 10 μL of β‐galactosidase (Component D) and 6-hour incubation. Azide-attached antibody was produced via an overnight reaction (30 °C) with UDP-GalNAz (Component E) and GalT enzyme (Component H). At the last step, DBCO-modified DNA oligos were added to the azide-attached antibody for 4-hour copper-free click chemistry action. During each step, the product of interest was purified via 50 K Amicon Ultra-0.5 mL Centrifugal Filters.

### Cell culture

Pan02 cells and NIH-3T3 cells were cultured in DMEM (high glucose) medium containing 10% FBS, 50 units/mL of penicillin, and 50 µg/mL of streptomycin at 37 °C in a 5% CO_2_ humidified environment incubator (Thermo Fisher Scientific, USA).

To get CAFs, NIH-3T3 cells were incubated with 10 ng/mL of TGF-β for 24 hours. To validate CAFs, cells suspensions were collected and incubated with Cy5-labeled anti-FAP-α antibody or Cy3-labeled anti-α-SMA antibody under room temperature for 20 minutes. After washing with cold PBS three times, 50 K cells were analyzed by flow cytometer (Cytomics™ FC 500, Beckman Coulter, Miami, FL, USA) to quantify the fluorescent intensity.

MOHITO cells were cultured in a six-well plate at 37 °C in a 5% CO_2_ humidified environment incubator (Thermo Fisher Scientific, USA). The medium for this cell line was Prigrow II medium (abm, USA) containing 20% FBS, 10 ng/mL mouse IL-7, 50 units/mL of penicillin, and 50 µg/mL of streptomycin.

### Cellular uptake assays

For quantitative analysis, cells were seeded into six-well plates at the density of 100 K cells per well and cultured for 24 hours. Cy5-labeled DMS, antiCAFs-DMS, or antiCAFs-DMS-AptT were added to cells, at the final Cy5 concentration of 2 mg/mL, for 4-hour incubation. Cells were washed with cold PBS twice, trypsinized and resuspended in 0.5 mL of PBS. The Cy5 intensity of cells was measured by a flow cytometer (Cytomics™ FC 500, Beckman Coulter, Miami, FL, USA). 50 K cells were recorded for each sample.

For microscopic imaging, cells, at the density of 10 K cells per well, were seeded into 8-well chamber (Millicell® EZ SLIDES, Merck Millipore) 24 hours prior to adding our nano-formulations. After 4-hour incubation, cells in wells were washed with cold PBS for three times and followed by fixing in 4% (v/v) paraformaldehyde. Nucleus were stained with Fluoroshield Mounting Medium with DAPI (Abcam). Fluorescence imaging was performed on Axio Imager.M2 (Zeiss, Germany).

### Cell viability assay

We performed an ATP-based luminescent cell viability assay using CellTiter-Glo Luminescent Cell Viability Assays (Promega). Cells were seeded into 96-well opaque white polystyrene microplate (Corning), at the density of 20 K cells per well, and cultured for 24 hours. Cells were incubated with various concentrations of AG17724, DMS, antiCAFs-DMS, or antiCAFs-DMS-AptT. After 48 hours, plates were equilibrated at room temperature for 0.5 hour. Cells were lysed in 100 μL of CellTiter-Glo reagent (Promega) for 2 minutes. After incubating at room temperature for 10 minutes, the luminescence was measured on a multimode microplate reader (Thermo Fisher Scientific Varioskan Flash, USA).

### Western blotting

Cells of interest were harvested and lysed by cell lysis buffer (Beyotime, China). Electrophoresis of samples was run on homemade 10% SDS-PAGE gels. Then samples were transferred from the gel to polyvinylidene fluoride (PVDF) films, which were next incubated with anti- primary antibodies (1:1 K) for 24 overnight. PVDF films were incubated with HRP-labeled goat anti-rabbit secondary antibodies (1:5 K) and washed. At the last, secondary antibodies were detected by Immobilon Western HRP Substrate (Millipore, Billerica, USA) on ChemiScope 6000 Touch System (Shanghai, China).

### Indirect co-culture of pancreatic cancer spheroids and CAFs

To culture pancreatic cancer spheroids, at 37 °C in a 5% CO2 humidified environment incubator (Thermo Fisher Scientific, USA), 3 K Pan02 cells (per well) were seeded into Ultra Low Attachment 96-well plate with the round bottom (Corning) for 7-day culture in DMEM (high glucose) with 20% FBS, 50 units/mL of penicillin and 50 µg/mL of streptomycin.

For indirect co-culture experiment, 10 organoids were transferred into 50-mL GFR Matrigel (356231, Corning) in DMEM (high glucose) with 20% FBS, 50 units/mL of penicillin, and 50 µg/mL of streptomycin. 50 K CAFs, which were pre-incubated with 0.5 μM of AG17724 or antiCAFs-DMS, were then seeded on the top of Matrigel for 7 days, followed by recording organoid growth using Cyntellect Celigo (Cyntellect) and analyzing the organoid size using Cyntellect Celigo software (version 1.3, Cyntellect).

### CD8^+^ T cell binding assay

200 K of MOHITO cells per well in six-well plate were cultured for 24 hours. Cy5-labeled antiCAFs-DMS or antiCAFs-DMS-AptT were added to cells, at the final Cy5 concentration of 2 mg/mL, for 0.5-hour, 1-hour or 4-hour incubation. After washing with cold PBS twice, cells were resuspended in 0.5 mL of PBS. The Cy5 intensity of cells was measured by a flow cytometer (Cytomics™ FC 500, Beckman Coulter, Miami, FL, USA).

### Confocal microscopy study of cell–cell interaction

10 K of CAFs per well were seeded into eight-well chamber (Millicell® EZ SLIDES, Merck Millipore) for overnight culture and then stained by CellTracker™ Red CMTPX Dye (Invitrogen, USA) and Hoechst (Invitrogen, USA) for 10 minutes at 37 °C. In parallel, 50 K of MOHITO cells per well in a six-well plate were cultured for overnight culture and then stained by CellTracker™ Green CMFDA Dye (Invitrogen, USA) and Hoechst (Invitrogen, USA) for 10 minutes at 37 °C. Washing CAFs and MOHITO cells with corresponding cell culture medium (4 °C) to remove the unstained solution. MOHITO cells were then added to CAFs for co-culture, and they were treated with antiCAFs-DMS or antiCAFs-DMS-AptT (control the concentration of AG17724 at 0.1 μM) at 4 °C for 2 hours. Then, cells were fixed with paraformaldehyde at a final concentration of 1% (w/v), and the images of cell–cell complexes were observed by confocal microscopy (TCS SP5 AOBS confocal microscopy system, Leica, Germany).

### Subcutaneous PDAC model, biodistribution, and antitumor assay

We established the subcutaneous PDAC model by subcutaneous inoculation (at the right back of 6-week-old female C57BL/6 mice) of 100-μL mixed cell suspension containing 1000 K Pan02 cells and 500 K NIH-3T3 cells. After 2 weeks, tumors grew to the volume of around 180 mm^3^.

For biodistribution imaging, nine subcutaneous PDAC-bearing mice were randomly divided into three groups. Alexa750-labeled DMS, antiCAFs-DMS, and antiCAFs-DMS-AptT were intravenously injected at 200 mg/kg of Alexa750. After 4 hours, mice were euthanized and tumors and organs were imaged using the IVIS Spectrum system (Caliper Life Sciences, Hopkinton, MA, USA). Then, tumors and organs were homogenized in PBS and placed in 96-well plate for imaging.

For antitumor assay, 40 tumor-bearing mice were randomly divided into 4 groups (10 per group). Treatments started on the 14th day after tumor inoculation. Treatment was carried out once every 3 days for in total of seven rounds. Drugs were injected via the tail vein. Body weights of mice and tumor volumes were recorded every 3 days. Mice were euthanized once tumor volumes of them reached 1200 mm^3^ (which is the maximal tumor size permitted by our Animal Experimentation Ethics Committee). Tumor volumes were measured and calculated using the formula ½ × length × width^2^.

### Cell population analysis of tumors

For tumor dissociation, collected tumors were dissociated into a single cell suspension using the Tumor Dissociation Kit, mouse (Miltenyi Biotec, Nordics) in combination with the gentleMACS Octo Dissociator with heaters (Miltenyi Biotec, Nordics) according to the manufacturer’s instructions.

For isolation and counting CAFs via FACS, single-cell suspensions of tumors, 200 K cells per sample, were suspended in 50 μL of PBS (pH 7.2), 2 mM EDTA, and 0.5% BSA (PEB) buffer. Cy3-labeled anti-α-SMA antibody was added to cell suspensions and incubated under 4 °C for 10 minutes. After washing via PEB twice, samples were stained with 5 μg/mL propidium iodide (Miltenyi Biotec) immediately before analysis using the MACSQuant™ Analyzer (Miltenyi Biotec).

For CD8^+^ T cell analysis in single-cell suspensions of tumors, single-cell suspensions of tumors were firstly incubated with 12.5 μg/mL mouse IgG (Sigma-Aldrich, USA) PBS for 15 min on ice to block the unspecific binding of antibodies. FITC-labeled mouse anti-CD3 antibody, PE-labeled mouse anti-CD8 antibody, and Brilliant Violet 510™ anti-mouse CD45 antibody were diluted in flow buffer consisting of PBS with 10% FBS. Cell suspensions then were incubated with the antibody mix in 96 v-bottom well plates (Corning, Costar), on ice, in the dark, for 0.5 hour. Following the incubation, 100 μL of flow buffer was added to each well, and the plates were centrifuged at 410 × *g* for 6 minutes at 4 °C. Supernatants were discarded and cell pellets were resuspended in 150 μL of flow buffer per well and centrifuged again. Cell viability was assessed by 1 μg/mL propidium iodide prior to flow cytometric analysis. We also checked the expansion activity of isolated CD8^+^ T cells via counting the live and dead T cells (after trypan blue staining) with an automated cell counter (Countess™ 3, Invitrogen). Samples were then analyzed using the MACSQuant™ Analyzer (Miltenyi Biotec).

### Orthotopic model of pancreatic cancer and antitumor assay

We established the orthotopic murine pancreatic cancer model by surgical implantation of 500 K Pan02-Luc cells into the head of the pancreas of 6-week-old female C57BL/6 mice. Treatments, as described above, were started on the 14th-day post-implant. Bioluminescence imaging was conducted with the IVIS Spectrum system (Caliper Life Sciences, Hopkinton, MA, USA) to monitor the tumor developments. Before the imaging, d-Luciferin (PerkinElmer, USA), dissolved in DPBS, was injected intra-peritoneally into mouse (150 mg d-Luciferin/kg body weight). Mice were euthanized once their body weight decreased to 15 g (which is the humane endpoint permitted by our Animal Experimentation Ethics Committee).

### Statistics

All the statistical analyses were carried out in GraphPad Prism 9.

### Reporting summary

Further information on research design is available in the Nature Research Reporting Summary linked to this article.

## Supplementary information

Supplementary Information

Peer Review File

Reporting Summary

## Data Availability

The authors declare that the data that support the findings of this study are available within the Article and its Supplementary Information file. Source data are provided with this paper.

## Source data

Source data
