# Supplementary material for: Pancreatic tumor eradication via selective Pin1 inhibition in cancer-associated fibroblasts and T lymphocytes engagement
Source: Nat Commun. 2022 Jul 25;13:4308. doi: 10.1038/s41467-022-31928-7 (PMC9314377; doi:10.1038/s41467-022-31928-7)
Supplement: Supplementary file 1 — Supplementary Information [file 41467_2022_31928_MOESM1_ESM.pdf]

Supplementary Information

Pancreatic Tumor Eradication via PIN1 Inhibition in Cancer Associated Fibroblasts and T Lymphocytes Engagement

Jiaye Liu[*1,2,3,4], Yang Wang[*5], Chunyang Mu[*6], Meng Li[7,8], Kewei Li[9], Shan Li[10], Wenshuang Wu[1,2], Lingyao Du[11], Xiaoyun Zhang[6], Chuan Li[6], Wei Peng[6], Junyi Shen[6], Yang Liu[1,2], Dujiang Yang[12], Kaixiang Zhang[1,2], Qingyang Ning[1,2], Xiaoying Fu[12], Yu Zeng[13], Yinyun Ni[4], Zongguang Zhou[12], Yi Liu[14], Yiguo Hu[3], Xiaofeng Zheng[#15], Tianfu Wen[#6], Zhihui Li[#1,2], Yong Liu[#12]

[1]Department of Thyroid and Parathyroid Surgery, West China Hospital, Sichuan University, Chengdu, China
[2]Laboratory of Thyroid and Parathyroid diseases, Frontiers Science Center for Disease-Related Molecular Network, West China Hospital, Sichuan University, Chengdu, China
[3]State Key Laboratory of Biotherapy and Cancer Center, West China Hospital, Sichuan University and Collaborative Innovation Center, Chengdu, China
[4]Respiratory Health Institute, Frontiers Science Center for Disease-related Molecular Network, West China Hospital, Sichuan University, Chengdu, China
[5]Department of Medical Biochemistry and Biophysics, Karolinska Institute, Stockholm, Sweden
[6]Department of Liver Surgery & Liver Transplantation Center, West China Hospital, Sichuan University, Chengdu, China
[7]Guangzhou Institutes of Biomedicine and Health, Chinese Academy of Sciences, Guangzhou, China
[8]Guangzhou Regenerative Medicine and Health Guangdong Laboratory, Bioland Laboratory, Guangzhou, China
[9]Department of Pediatric Department, West China Hospital, Sichuan University, Chengdu, China
[10]Department of Hepatobiliary Surgery, Daping Hospital, Army Medical University, Chongqing, China
[11]Center of Infectious Diseases, West China Hospital, Sichuan University, Chengdu, China
[12]Department of Gastrointestinal Surgery, West China Hospital, Sichuan University, Chengdu, China
[13]Department of Gastroenterology, West China Hospital, Sichuan University, Chengdu, China
[14]Department of Rheumatology and Immunology, Rare Disease Center, West China Hospital, Sichuan University, Chengdu, China
[15]Department of Endocrinology and Metabolism, Center for Diabetes and Metabolism Research, West China Hospital, Sichuan University, Chengdu, China

*These authors contributed equally
# These authors jointly supervised this work

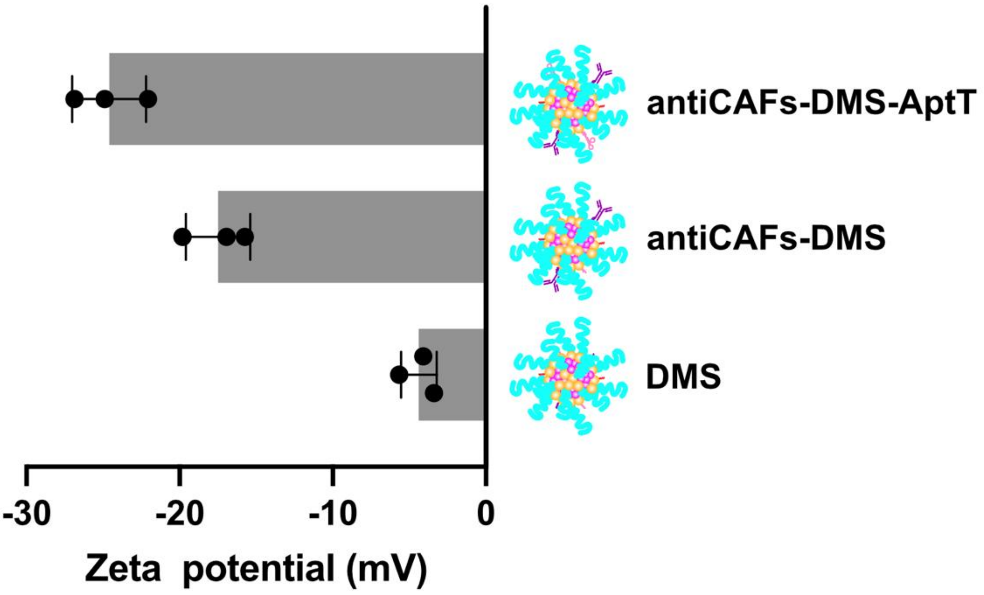

Supplementary Fig.1: Zeta potentials of DMS, antiCAFs-DMS and antiCAFs-DMS-AptT (n=3 biologically independent experiments). Error bars, mean ± SD. Source data are provided as a Source Data file.

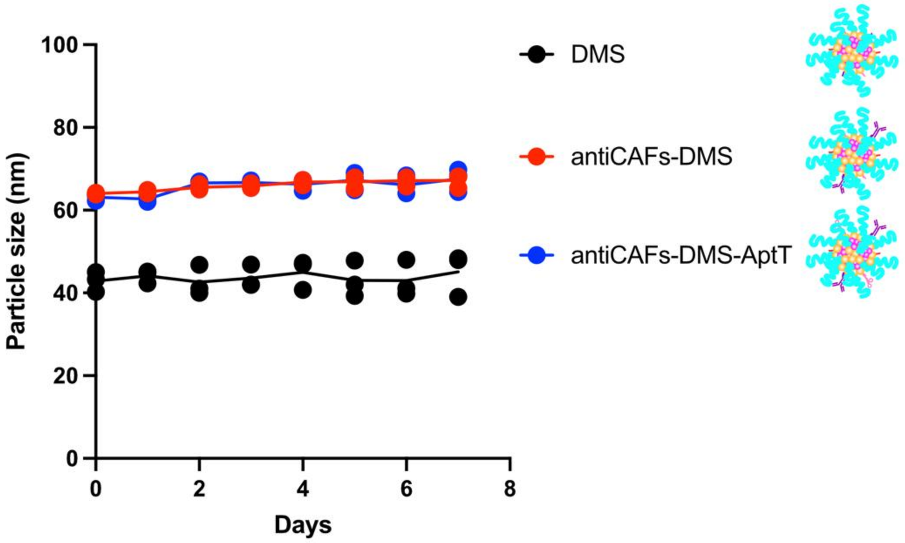

Supplementary Fig.2: Sizes changes of DMS, antiCAFs-DMS and antiCAFs-DMS-AptT in PBS containing 10% FBS for one week incubation under room temperature (n=3 biologically independent experiments). Error bars, mean ± SD. Source data are provided as a Source Data file.

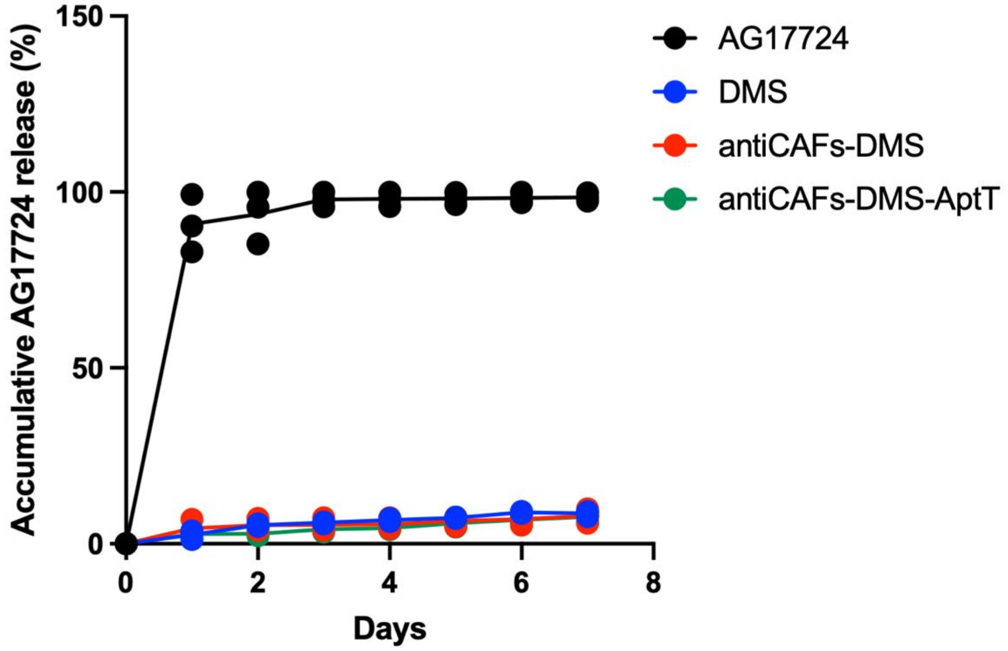

Supplementary Fig.3: Accumulative release of AG17724 from different preparations in PBS containing 10% FBS for one week under room temperature (n=3 biologically independent experiments). Error bars, mean ± SD. Source data are provided as a Source Data file.

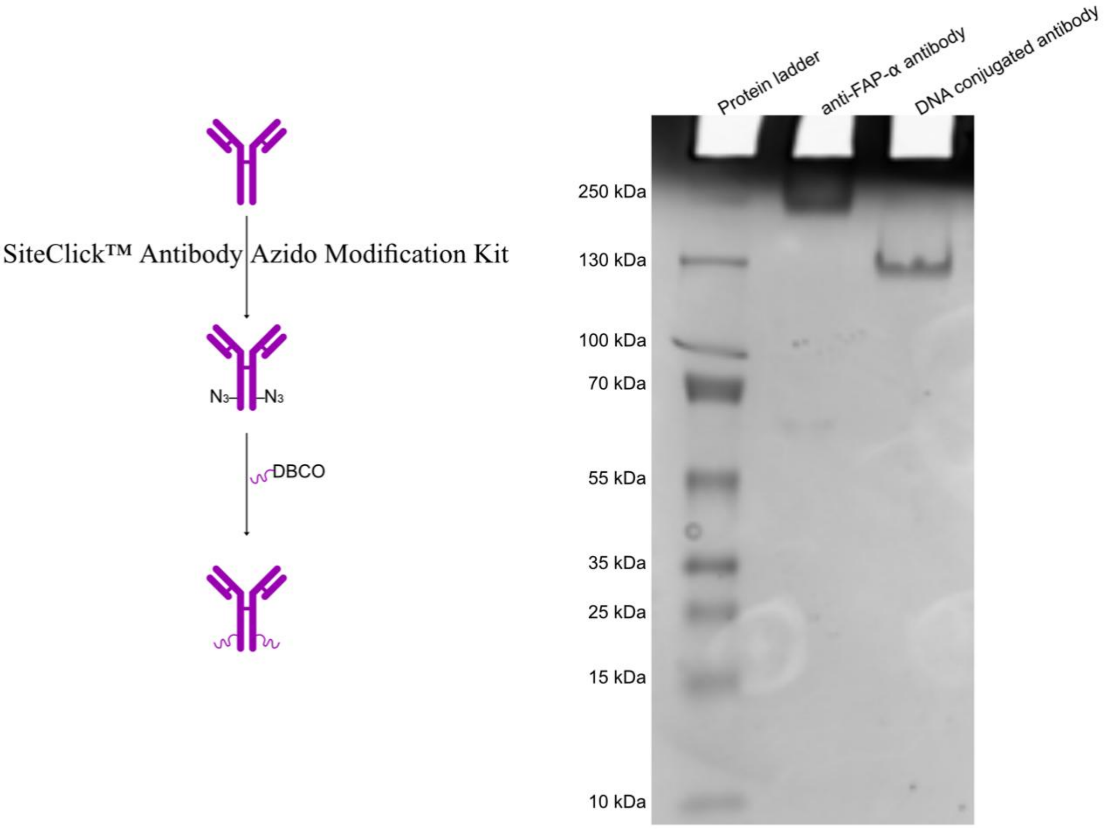

Supplementary Fig.4: DNA-antibody validation on 10% PAGE gel. The gel was stained with Coomassie. Three independent repetitions got similar results. Source data are provided as a Source Data file.

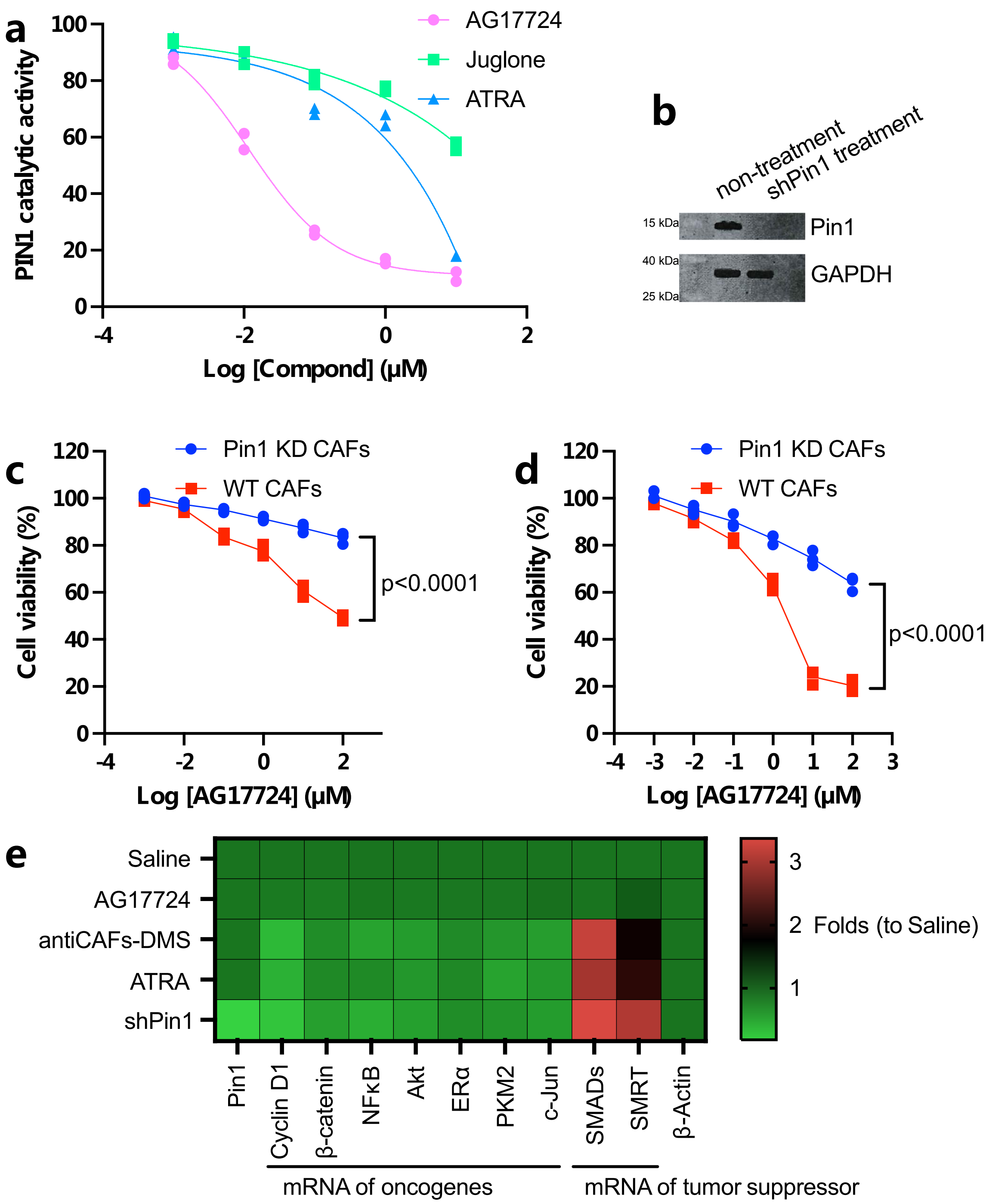

Supplementary Fig.5: AG17724 targets Pin1. (**a**), Inhibition of Pin1 catalytic activity by AG17724, Juglone or ATRA, as measured by PPIase assay (n=2 biologically independent experiments). (**b**), Western blot showing the decrease in Pin1 protein levels in shPin1 treated cells. WT and Pin1 KD CAFs were treated with indicated concentrations of AG17724 (**c**) or corresponding antiCAFs-DMS (**d**) for 48 hours, followed by MTT cell growth assay with the readout absorbance measured by 570 nm (n=3 biologically independent experiments). Two-way ANOVA was performed for the statistical analysis. (**e**), RT-qPCR analysis of gene expressions as indicated after 24-hour treatments with AG17724 (0.5 μM), antiCAFs-DMS (corresponds to 0.5 μM) and ATRA (25 μM). Mean values of three biological replicates is plotted in this heatmap. Source data are provided as a Source Data file.

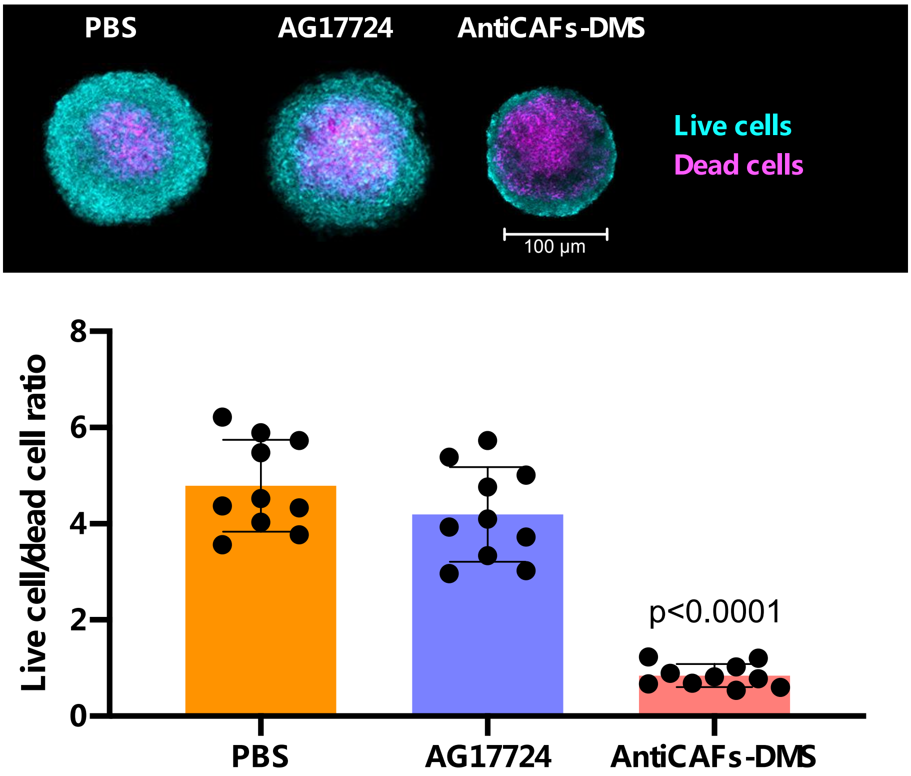

Supplementary Fig.6: Treated spheroids were stained using the viability/cytotoxicity assay kit to determine live and dead cells. Images were generated using maximum projection from a Z-stack of seven images 15 µm apart. Bar plot at the bottom shows the live cell to dead cell ratios of spheroids (n=10 biologically independent spheroids). Error bars, mean ± SD. Source data are provided as a Source Data file.

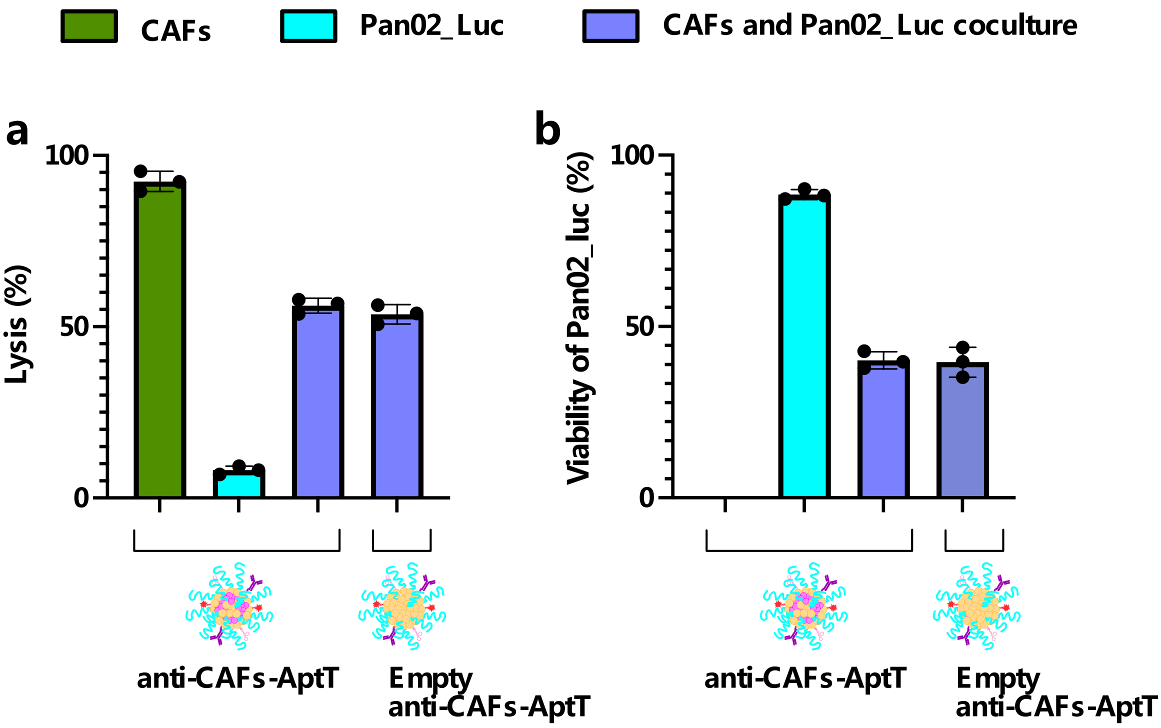

Supplementary Fig.7: Coculture experiment to study T cell-mediated cytotoxicity. $CD8^+$ T cells as effector cells at an effector-to-target ("target" means Pan02_Luc and CAFs together, and Pan02_Luc/CAFs = 2/1) ratio of 3:1. (**a**), Cell lysis assay. (**b**), Viability assay of Pan02_Luc cells via the method of bioluminescence (n=3 biologically independent experiments). Error bars, mean ± SD. Source data are provided as a Source Data file.

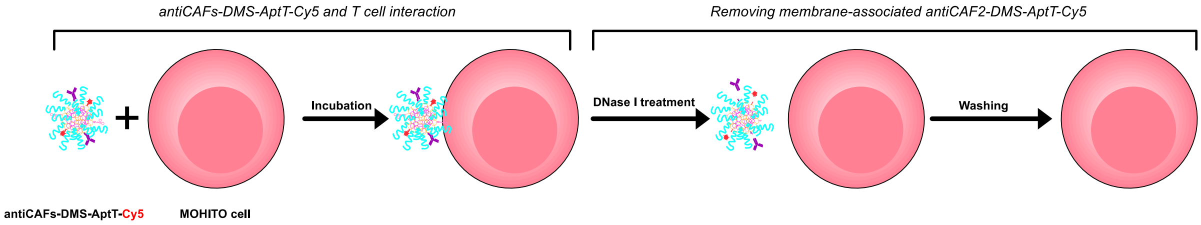

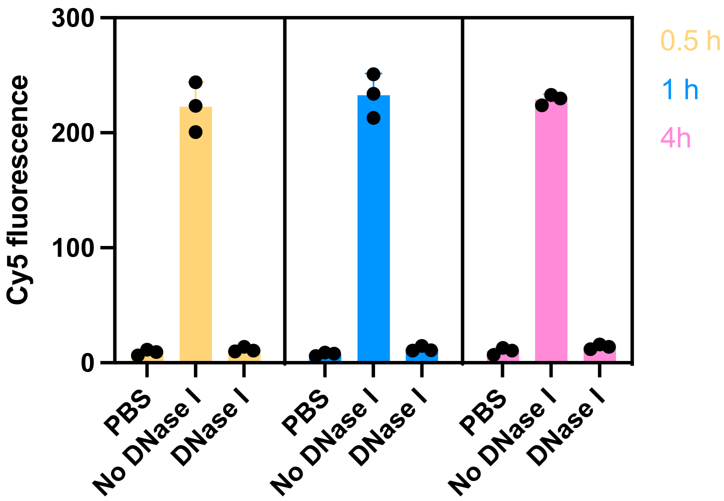

Supplementary Fig.8: DNase I treatment workflow (Top chart) to study the association of antiCAFs-DMS-AptT-Cy5 with CD8+ T cells. After different time points as indicated, Cy5 signals were detected and plotted (n=3 biologically independent experiments). Error bars, mean ± SD. Source data are provided as a Source Data file.

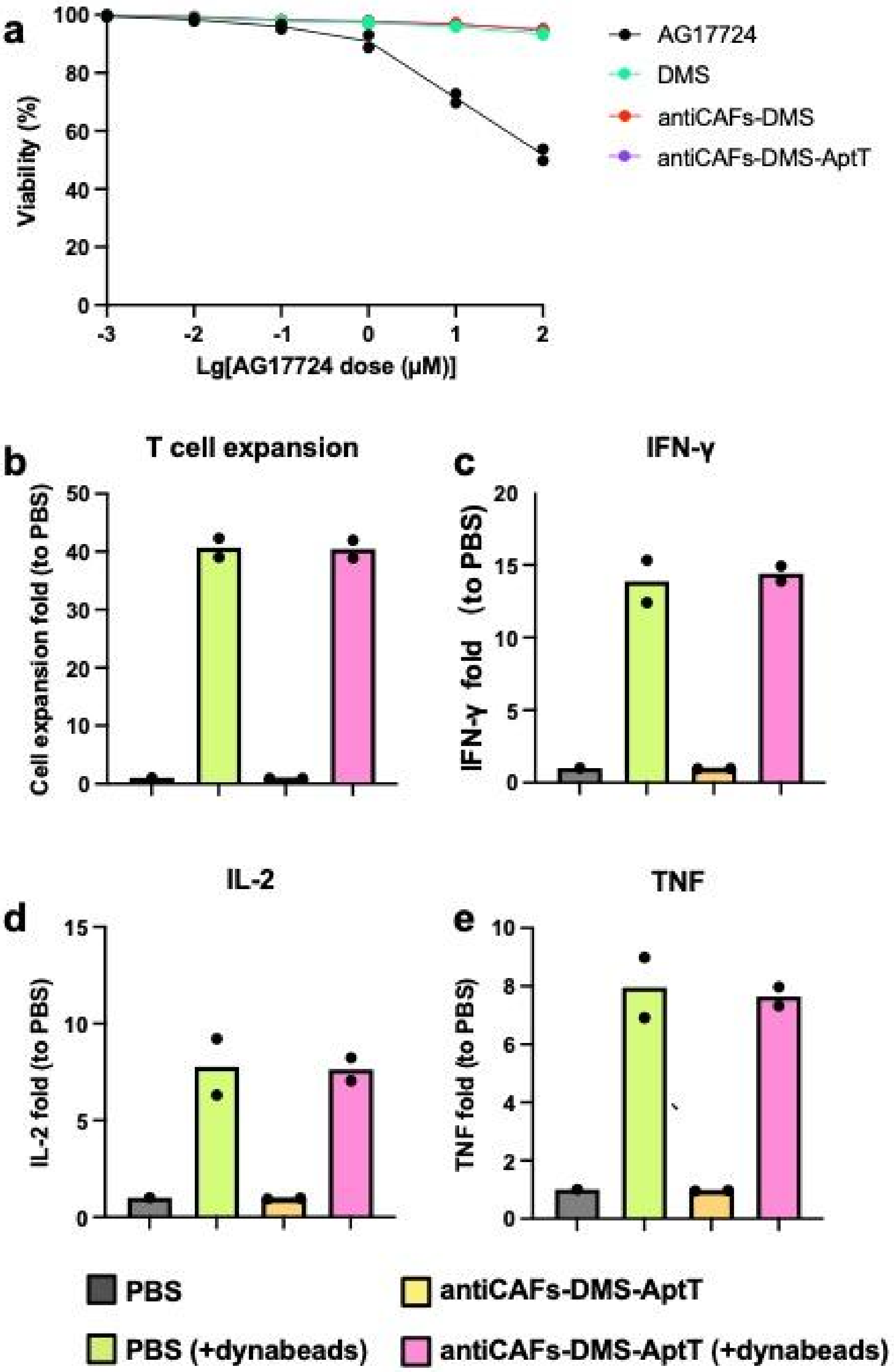

Supplementary Fig.9: CD8⁺ T cell viability and function assays. (**a**), Viability of CD8+ T cells with different treatments as indicated. (**b**) T cell expansion, (**c**) IFN-γ, (**d**) IL-2 and (**e**) TNF measurements after firstly treating the cells with/without antiCAFs-DMS-AptT for 48 hours then with/without antiCD3&antiCD28 Dynabeads for 6 days. n=2 biologically independent experiments. Source data are provided as a Source Data file.

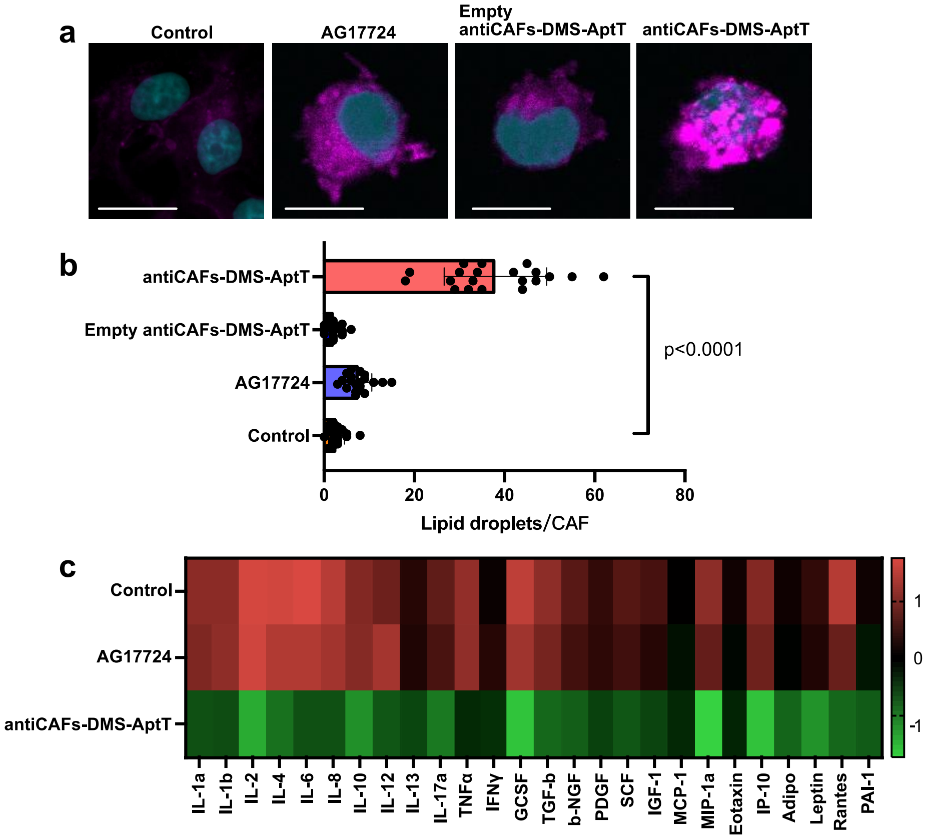

Supplementary Fig.10: PIN1 inhibition by antiCAFs-DMS-AptT causes quiescent CAFs and inhibits a wide range of cytokines. (**a-b**), lipid droplet accumulation assay on α-SMA positive CAFs isolated from tumor via incubation with saline(control), 0.5-μM AG17724 or antiCAFs-DMS-AptT for 24 hours (n=20 biologically independent cells). Scale bars, 50 μm. Error bars, mean ± SD; Two-tailed Student's t tests. (**c**), cytokine production using cytokine array. Source data are provided as a Source Data file.

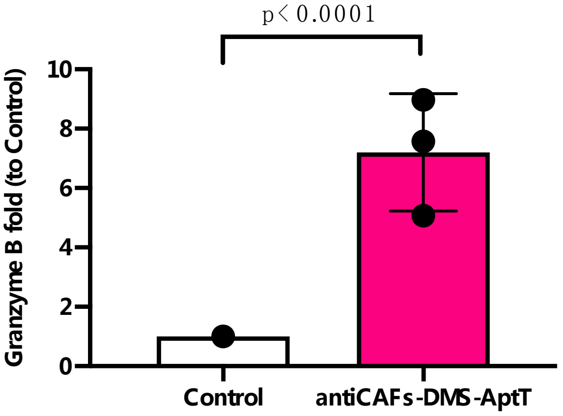

Supplementary Fig.11: qPCR analysis to compare DNA barcode1 in CAFs and non-CAFs cells of tumor (n=3 biologically independent experiments). Each dot stands for one mouse. Error bars, mean ± SD. Source data are provided as a Source Data file.

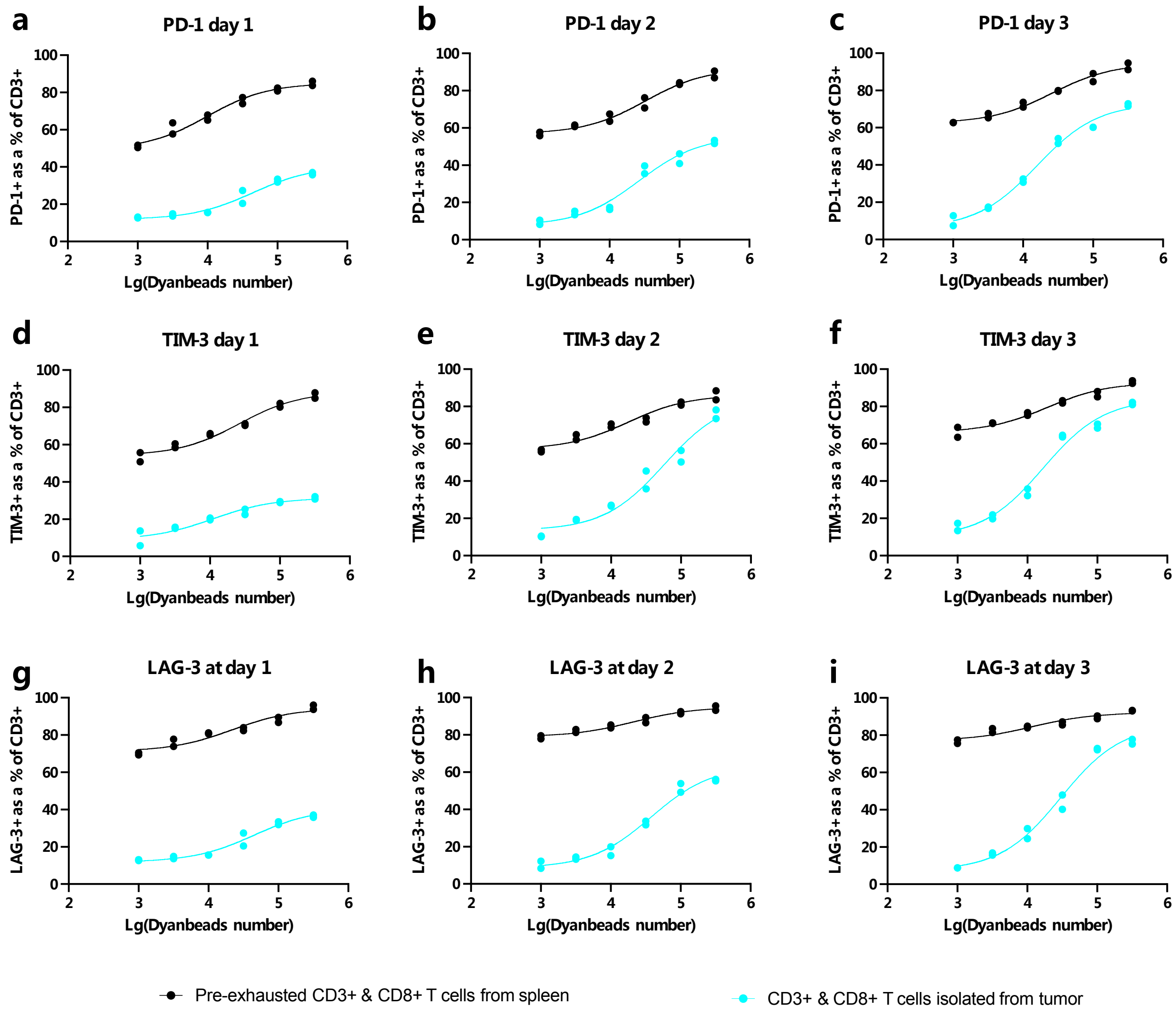

Supplementary Fig.12: T cell exhaustion assay via measuring the expressions of PD-1(**a-c**), TIM-3 (**d-f**) and LAG-3 (**g-i**). At the 25th day of antiCAFs-DMS-AptT treatment, CD3+ & CD8+ T cells were isolated from tumor. These cells or pre-exhausted CD3+ & CD8+ T cells were seeded at 200K/well. Exhausted T cells had been repeatedly stimulated (5 times total, every 2-3 days) with Dynabeads CD3/CD28 (1:1 bead-to-cell ratio). In-well, activation was induced with Dynabeads CD3/CD28. Every 24h, 10uL samples were analysed using the iQue® Mouse T Cell Kit. n=2 biologically independent animals. Source data are provided as a Source Data file.

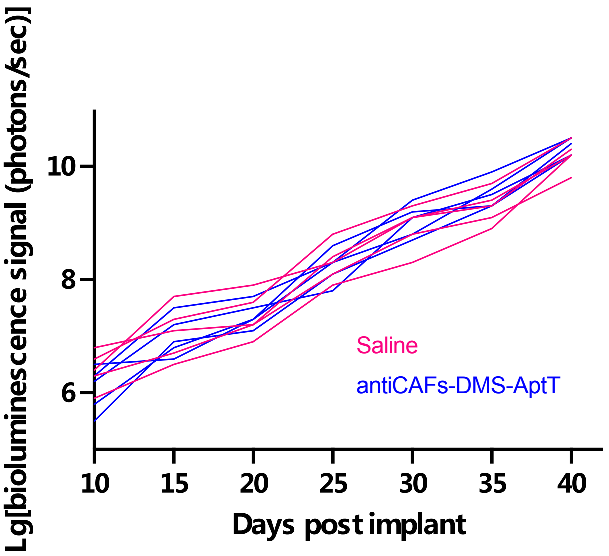

Supplementary Fig.13: Treatment effects of empty antiCAFs-DMS-AptT (no AG17724 encapsulation) on orthotopic pancreatic cancer model (5 mice per group). Tumor development of each mouse, quantified by bioluminescence signal. Source data are provided as a Source Data file.

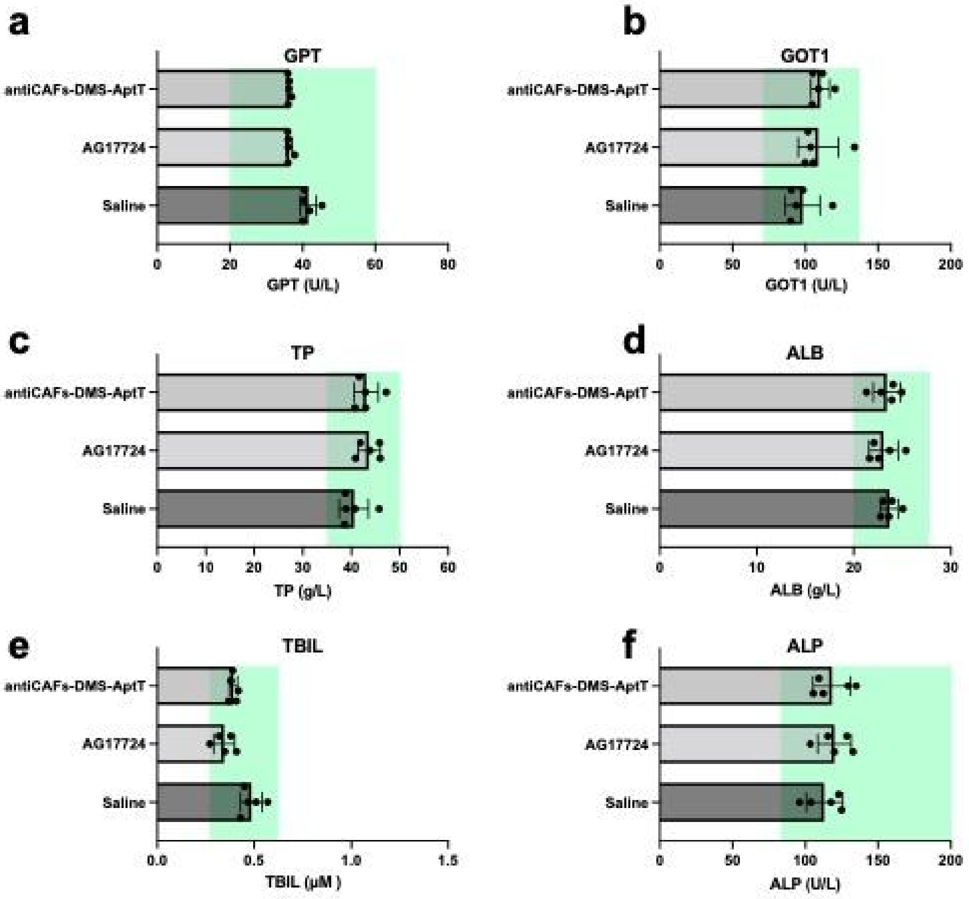

Supplementary Fig.14: Biochemistry results from mice (n=5 biologically independent animals) treated with saline, AG17724 or antiCAFs-DMS-AptT. (**a-f**) These results show mean and standard deviation of the biochemistry index as indicated at the 40th day of our treatment schedule. Green areas show the normal ranges. Source data are provided as a Source Data file.

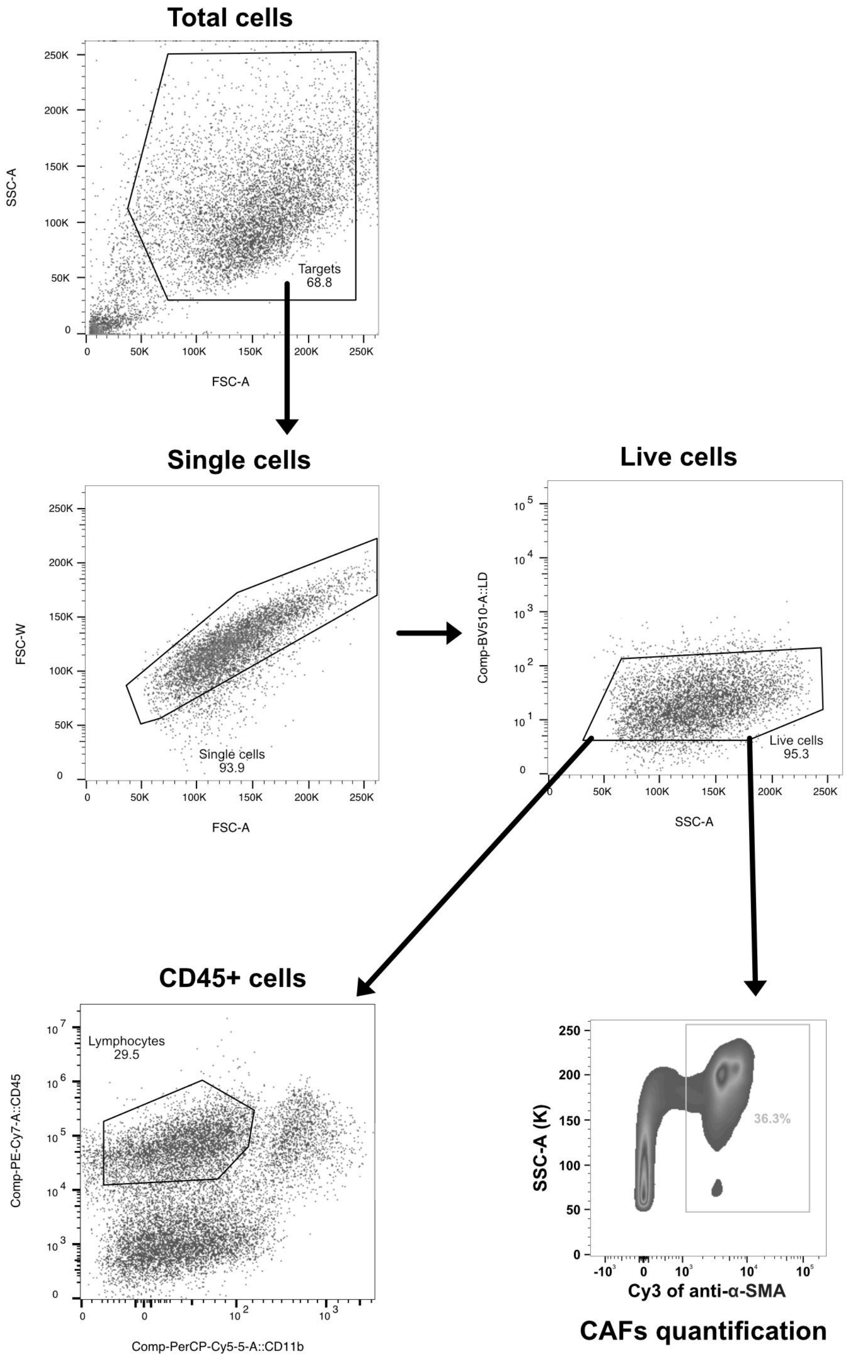

Supplementary Fig.15: Flow cytometry gating strategy. Lymphocytes (apply to Fig.6G, 6H and Fig.7F, 7G.) and CAFs (apply to Fig.6A, 6B and Fig.7E) of tumor tissue gating illustration. Source data are provided as a Source Data file.

Supplementary Table 1: Sequences of DNA barcodes.

Name	DNA-Cho	Producing Company
Barcode1-Cho	GTGATGGAGATTTATTCTCTT-Cho	Integrated DNA Technologies
Barcode2-Cho	AAAAGAATGATGATAATCATG-Cho	Integrated DNA Technologies
Barcode3-Cho	AGTACTGTGCTAAGATGGTGT-Cho	Integrated DNA Technologies

Supplementary Table 2: Probed sequences for DNA barcodes hybridization.

Name	Sequence-modification	Producing Company
Cy5-DNA for barcode1	AAGAGAATAAATCTCCATCAC-Cy5	Integrated DNA Technologies
Cy3.5-DNA for barcode2	CATGATTATCATCATTCTTTT-Cy3.5	Integrated DNA Technologies
Alexa488-DNA for barcode3	ACACCATCTTAGCACAGTACT-Alexa488	Integrated DNA Technologies

Supplementary Table 3: Sequence of DNA aptamer targeting $CD8^+$ T cells.

Name	Sequence-modification	Producing Company
Handle-Aptamaer	AAGAGAATAAATCTCCATCACCCAG AGTGACGCAGCAACAGAGGTGTAGA AGTACCGTGAACAAGCTTGAAATTG TCTCTGACAGAGGTGGACACGGTGG CTTTTAGT	Integrated DNA Technologies
