# Supplementary material for: Pancreatic tumor eradication via selective Pin1 inhibition in cancer-associated fibroblasts and T lymphocytes engagement
Source: Nat Commun. 2022 Jul 25;13:4308. doi: 10.1038/s41467-022-31928-7 (PMC9314377; doi:10.1038/s41467-022-31928-7)
Supplement: Supplementary file 3 — Reporting Summary [file 41467_2022_31928_MOESM3_ESM.pdf]

nature portfolio

Corresponding author(s): Yong Liu

Last updated by author(s): Jun 29, 2022

Reporting Summary

Nature Portfolio wishes to improve the reproducibility of the work that we publish. This form provides structure for consistency and transparency in reporting. For further information on Nature Portfolio policies, see our Editorial Policies and the Editorial Policy Checklist.

Statistics

For all statistical analyses, confirm that the following items are present in the figure legend, table legend, main text, or Methods section.

n/a	Confirmed	
☐	☒	The exact sample size (n) for each experimental group/condition, given as a discrete number and unit of measurement
☐	☒	A statement on whether measurements were taken from distinct samples or whether the same sample was measured repeatedly
☐	☒	The statistical test(s) used AND whether they are one- or two-sided<br>*Only common tests should be described solely by name; describe more complex techniques in the Methods section.*
☐	☒	A description of all covariates tested
☐	☒	A description of any assumptions or corrections, such as tests of normality and adjustment for multiple comparisons
☐	☒	A full description of the statistical parameters including central tendency (e.g. means) or other basic estimates (e.g. regression coefficient) AND variation (e.g. standard deviation) or associated estimates of uncertainty (e.g. confidence intervals)
☐	☒	For null hypothesis testing, the test statistic (e.g. F, t, r) with confidence intervals, effect sizes, degrees of freedom and P value noted<br>*Give P values as exact values whenever suitable.*
☒	☐	For Bayesian analysis, information on the choice of priors and Markov chain Monte Carlo settings
☒	☐	For hierarchical and complex designs, identification of the appropriate level for tests and full reporting of outcomes
☒	☐	Estimates of effect sizes (e.g. Cohen's d, Pearson's r), indicating how they were calculated

Our web collection on statistics for biologists contains articles on many of the points above.

Software and code

Policy information about availability of computer code

Data collection	Flow cytometry samples were collected using Cytomics FC 500 with CXP Software (Beckman Coulter) . Fluorescent microscopic imaging data was collected using ZEN microscopy software (Zeiss); Confocal imaging data was collected using Leica Application suiteX LAS X software (Leica). Live animal imaging was acquired using Living Image Software (IVIS Imaging Systems, Caliper Life Sciences).
Data analysis	GraphPad Prism (9.0.2), FIJI (ImageJ 1.48-1.53c), FlowJo (Version 10).

For manuscripts utilizing custom algorithms or software that are central to the research but not yet described in published literature, software must be made available to editors and reviewers. We strongly encourage code deposition in a community repository (e.g. GitHub). See the Nature Portfolio guidelines for submitting code & software for further information.

Data

Policy information about availability of data

All manuscripts must include a data availability statement. This statement should provide the following information, where applicable:

- Accession codes, unique identifiers, or web links for publicly available datasets
- A description of any restrictions on data availability
- For clinical datasets or third party data, please ensure that the statement adheres to our policy

The authors declare that all data supporting the findings of this study are available within the article and its supplementary information. Source data are provided with this paper. The source data are compiled as a source data file.

Field-specific reporting

Please select the one below that is the best fit for your research. If you are not sure, read the appropriate sections before making your selection.

- [x] Life sciences
- [ ] Behavioural & social sciences
- [ ] Ecological, evolutionary & environmental sciences

For a reference copy of the document with all sections, see nature.com/documents/nr-reporting-summary-flat.pdf

Life sciences study design

All studies must disclose on these points even when the disclosure is negative.

Sample size	For the in vitro studies, no statistic method was used to predetermine sample size. We followed the conventional way of quantification accepted in our published paper (PMID: 35111955, 34650055) to determine the sample size. The minimum of three independent measurements were used allowing to perform statistical tests.<br>For in vitro studies, We followed the previous experience maintaining the balance between the statistical significance and minimizing the number of used animals., and following already published papers in the context of cancer treatment (PMID: 35111955, 32327656).
Data exclusions	No data was excluded from the study.
Replication	Biological replicates of each experiment in each panel were indicated in the figure legends, and all attempts at replication were successful.
Randomization	For both in vivo and in vitro studies, allocation of samples and organisms into experimental groups was random.
Blinding	The investigators were not blinded for the allocation of groups during experiments. Image analysis was conducted blinded, , because the researchers needed to keep track of the experiments and the analysis was performed by the same personnel. Fully blinded animal experiments were not possible due to personnel availability to accommodate such situations.

Reporting for specific materials, systems and methods

We require information from authors about some types of materials, experimental systems and methods used in many studies. Here, indicate whether each material, system or method listed is relevant to your study. If you are not sure if a list item applies to your research, read the appropriate section before selecting a response.

Materials & experimental systems

n/a	Involved in the study	
[ ]	[x]	Antibodies
[ ]	[x]	Eukaryotic cell lines
[x]	[ ]	Palaeontology and archaeology
[ ]	[x]	Animals and other organisms
[x]	[ ]	Human research participants
[x]	[ ]	Clinical data
[x]	[ ]	Dual use research of concern

Methods

n/a	Involved in the study	
[x]	[ ]	ChIP-seq
[ ]	[x]	Flow cytometry
[x]	[ ]	MRI-based neuroimaging

Antibodies

Antibodies used

anti-FAP-α antibody (#MAB9727-100, Clone #983802, R&D SYSTEMS, Species reactivity: mouse. Use: Flow Cytometry. Working concentration: 0.25 μg/mL (10E06 cells).).
anti-α-SMA antibody (#ab5694, abcam, Species reactivity: mouse, human. Use: Flow Cytometry. Working concentration: 1 μg/mL (10E06 cells).).
anti-PIN1 antibody (#EPR18546-317, abcam, Species reactivity: mouse, rat, human. Use: WB. Working dilution: 1/2000.).
anti-β-catenin antibody [IGX4794R-3] (#ab223075, abcam, Species reactivity: mouse, human. Use: WB. Working concentration: 1 μg/mL.).
anti-NF-κB p65 antibody [E379] (#ab207297, abcam, Species reactivity: mouse, human. Use: WB. Working concentration: 1 μg/mL.).
anti-AKT1 (phospho T308) antibody [AktT308-G12] (#ab278565, abcam, Species reactivity: mouse, rat, human. Use: WB. Working concentration: 0.1 μg/mL.).
anti-phospho-Akt (Thr308) antibody (#05-802R, Clone #50-1C-25, Sigma-Aldrich, Species reactivity: human, mouse, rabbit, chicken, bovine. Use: WB. Working dilution: 1/1500.).
anti-phospho-CDC2/CDK1 (Y15) antibody (#AF888-SP, R&D SYSTEMS, Species reactivity: mouse, rat, human. Use: WB. Working concentration: 0.2 μg/mL.).
anti-CDK1 antibody (A17) (#33-1800, ThermoFisher SCIENTIFIC, Species reactivity: mouse. Use: WB. Working concentration: 1 μg/mL.).
anti-GAPDH antibody [6C5] (#ab8245, abcam, Species reactivity: mouse, rat, human. Use: WB. Working dilution: 1/2000.).
HRP-conjugated goat anti-mouse IgG (H+L) cross-adsorbed secondary antibody (#G-21040, ThermoFisher SCIENTIFIC, Species

	reactivity: mouse. Use: WB. Working dilution: 1/10000.).<br>FITC-labeled anti-CD3 antibody (17A2) (#11-0032-82, ThermoFisher SCIENTIFIC, Species reactivity: mouse. Use: Flow Cytometry. Working concentration: 1 μg/mL.).<br>PE-labeled mouse anti-CD8 antibody (#12-0081-82, ThermoFisher SCIENTIFIC, Species reactivity: mouse. Use: Flow Cytometry. Working concentration: 1 μg/mL.).<br>Brilliant Violet 510™ anti-mouse CD45 antibody (#103137, Clone 30-F11, Biolegend, Species reactivity: mouse. Use: Flow Cytometry. Working concentration: 1 μg/mL.).
Validation	Antibodies were obtained commercially and their validation data are available on the manufacturer's website:<br>anti-FAP-α antibody (#MAB9727-100, Clone # 983802, R&D SYSTEMS, Species reactivity: mouse.), https://www.rndsystems.com/products/mouse-fibroblast-activation-protein-alpha-fap-antibody-983802_mab9727<br>anti-α-SMA antibody (#ab5694, abcam, Species reactivity: mouse, human.), https://www.abcam.com/alpha-smooth-muscle-actin-antibody-ab5694.html?productWallTab=ShowAll<br>a mouse anti-PIN1 antibody (#EPR18546-317, abcam, Species reactivity: mouse, rat, human.), https://www.abcam.com/pin1-antibody-epr18546-317-ab192036.html<br>anti-β-catenin antibody [IGX4794R-3] (#ab223075, abcam, Species reactivity: mouse, human.), https://www.abcam.com/beta-catenin-antibody-igx4794r-3-ab223075.html<br>anti-NF-κB p65 antibody [EPR20592-246] (#ab281081, abcam, Species reactivity: mouse.), https://www.abcam.com/nf-kb-p65-antibody-epr20592-246-bsa-and-azide-free-detector-ab281081.html<br>anti-AKT1 (phospho T308) antibody [AktT308-G12] (#ab278565, abcam, Species reactivity: mouse, rat, human.), https://www.abcam.com/akt1-phospho-t308-antibody-aktt308-g12-ab278565.html<br>anti-GAPDH antibody [6C5] (#ab8245, abcam, Species reactivity: mouse, rat, human.), https://www.abcam.com/gapdh-antibody-6c5-loading-control-ab8245.html<br>HRP-conjugated goat anti-mouse IgG (H+L) cross-adsorbed secondary antibody (#G-21040, ThermoFisher SCIENTIFIC, Species reactivity: mouse.), https://www.thermofisher.com/antibody/product/Goat-anti-Mouse-IgG-H-L-Cross-Adsorbed-Secondary-Antibody-Polyclonal/G-21040<br>FITC-labeled anti-CD3 antibody (17A2) (#11-0032-82, ThermoFisher SCIENTIFIC, Species reactivity: mouse.), https://www.thermofisher.com/antibody/product/CD3-Antibody-clone-17A2-Monoclonal/11-0032-82<br>PE-labeled mouse anti-CD8 antibody (#12-0081-82, ThermoFisher SCIENTIFIC, Species reactivity: mouse.), https://www.thermofisher.com/antibody/product/CD8a-Antibody-clone-53-6-7-Monoclonal/12-0081-82<br>TGF beta-1 Monoclonal Antibody (F.888.7) (#MA5-15065, ThermoFisher SCIENTIFIC, Species reactivity: mouse, rat, human.), https://www.thermofisher.com/antibody/product/TGF-beta-1-Antibody-clone-F-888-7-Monoclonal/MA5-15065<br>Brilliant Violet 510™ anti-mouse CD45 antibody (#103137, Clone 30-F11, Biolegend, Species reactivity: mouse.), https://www.biolegend.com/de-at/products/brilliant-violet-510-anti-mouse-cd45-antibody-7995<br>anti-phospho-Akt (Thr308) antibody (#05-802R, Clone #50-1C-25, Sigma-Aldrich, Species reactivity: human, mouse, rabbit, chicken, bovine.), https://www.sigmaaldrich.com/SE/en/product/mm/05802r<br>anti-phospho-CDC2/CDK1 (Y15) antibody (#AF888-SP, R&D SYSTEMS, Species reactivity: mouse, rat, human.), https://www.rndsystems.com/products/human-mouse-rat-phospho-cdc2-cdk1-y15-antibody_af888<br>anti-CDK1 antibody (A17) (#33-1800, ThermoFisher SCIENTIFIC, Species reactivity: mouse.), https://www.thermofisher.com/antibody/product/CDK1-Antibody-clone-A17-Monoclonal/33-1800

Eukaryotic cell lines

Policy information about cell lines

Cell line source(s)	Pan02 cell line and NIH-3T3 cell line were purchased from ATCC (USA). Pan02-Luc cell line was purchased from Labcorp (USA). Immortalized mouse CD4+ CD8+ T cell line (MOHITO) were purchased from abm (USA).
Authentication	These cell lines are authenticated by the Sichuan University Characterized Cell Line Core Facility by STR profiling.
Mycoplasma contamination	All cell lines were tested for and found to be free of mycoplasma contamination.
Commonly misidentified lines (See ICLAC register)	No commonly misidentified cell lines were used in the study.

Animals and other organisms

Policy information about studies involving animals; ARRIVE guidelines recommended for reporting animal research

Laboratory animals	6-8 week old female C57BL/6 mice were purchased from Chengdu Dashuo Biological Institute (Chengdu, China). All mice were maintained under ambient room temperature (22 °C) with 40%-70% humidity and light/dark cycle of 12 hours/12 hours.
Wild animals	No wild animals were used in this study.
Field-collected samples	No field collected samples were used in this stud.
Ethics oversight	All animal experimental procedures were approved by the Animal Experimentation Ethics Committee of Sichuan University.

Note that full information on the approval of the study protocol must also be provided in the manuscript.

Flow Cytometry

Plots

Confirm that:

- [x] The axis labels state the marker and fluorochrome used (e.g. CD4-FITC).
- [x] The axis scales are clearly visible. Include numbers along axes only for bottom left plot of group (a 'group' is an analysis of identical markers).
- [x] All plots are contour plots with outliers or pseudocolor plots.
- [x] A numerical value for number of cells or percentage (with statistics) is provided.

Methodology

Sample preparation	1, cultured cell flow cytometry: cells were seeded into six-well plates at the density of 100K cells per well and cultured for 24 hours. Cy5-labeled DMS, antiCAFs-DMS or antiCAFs-DMS-AptT were added to cells, at the final Cy5 concentration of 2 mg/mL, for 4-hour incubation. Cells were washed with cold PBS twice, trypsinized and resuspended in 0.5 mL of PBS. The Cy5 intensity of cells was measured by a flow cytometer.<br>2, tissue cell flow cytometry: collected tumors were dissociated into a single cell suspension using the Tumor Dissociation Kit, mouse in combination with the gentleMACS Octo Dissociator with heaters according to the manufacturer's instructions. For isolation and counting CAFs via FACS, single cell suspensions of tumors, 200K cells per sample, were suspended in 50 µL of PBS (pH 7.2), 2 mM EDTA, and 0.5% BSA (PEB) buffer. Cy3-labled anti-α-SMA antibody was added to cell suspensions and incubated under 4 °C for 10 minutes. After washing via PEB twice, samples were stained with 5 µg/mL propidium iodide (Miltenyi Biotec) immediately before analysis using the MACSQuant™ Analyzer (Miltenyi Biotec). For CD8+ T cell analysis in single cell suspensions of tumors, single cell suspensions of tumors were firstly incubated with 12.5 µg/mL mouse IgG (Sigma-Aldrich, USA) PBS for 15 min on ice to block unspecific binding of antibodies. FITC-labeled mouse anti-CD3 antibody, PE-labeled mouse anti-CD8 antibody and Brilliant Violet 510™ anti-mouse CD45 antibody were diluted in flow buffer consisting of PBS with 10% FBS. Cell suspensions then were incubated with the antibody mix in 96 v-bottom well plates (Corning, Costar), on ice, in the dark, for 0.5 hour. Following the incubation 100 µL of flow buffer was added to each well, and the plates were centrifuged at 410 g for 6 minutes at 4°C. Supernatants were discarded and cell pellets were re-suspended in 150 µL of flow buffer per well and centrifuged again. Cell viability was assessed by 1 µg/mL propidium iodide prior to flow cytometric analysis. Samples were then analyzed using the MACSQuant™ Analyzer (Miltenyi Biotec).
Instrument	Cytomics™ FC 500 cytometer (Beckman Coulter)
Software	FlowJo
Cell population abundance	For in vitro cultured-cell flow cytometry analysis, no cell sorting was performed for the experiment of flow cytometry.
Gating strategy	The first gating step was to get cells on the basis of the cells' forward scatter (FSC-A) and side scatter (SSC-A) properties.<br>Second, single cells were identified by plotting their FSC-A and FSC-W.<br>Third, live cells were identified based on CD45+ (with BV510 labeled CD45 antibody).<br>The last, cell populations were identified by different labeled antibodies.

- [x] Tick this box to confirm that a figure exemplifying the gating strategy is provided in the Supplementary Information.
