# Supplementary material for: Pancreatic tumor eradication via selective Pin1 inhibition in cancer-associated fibroblasts and T lymphocytes engagement
Source: Nat Commun. 2022 Jul 25;13:4308. doi: 10.1038/s41467-022-31928-7 (PMC9314377; doi:10.1038/s41467-022-31928-7)
Supplement: Supplementary file 2 — Peer Review File [file 41467_2022_31928_MOESM2_ESM.pdf]

Peer Review File

Pancreatic Tumor Eradication via Selective PIN1 Inhibition in Cancer Associated Fibroblasts and T Lymphocytes Engagement

Open Access This file is licensed under a Creative Commons Attribution 4.0 International License, which permits use, sharing, adaptation, distribution and reproduction in any medium or format, as long as you give appropriate credit to the original author(s) and the source, provide a link to the Creative Commons license, and indicate if changes were made. In the cases where the authors are anonymous, such as is the case for the reports of anonymous peer reviewers, author attribution should be to 'Anonymous Referee' followed by a clear attribution to the source work. The images or other third party material in this file are included in the article's Creative Commons license, unless indicated otherwise in a credit line to the material. If material is not included in the article's Creative Commons license and your intended use is not permitted by statutory regulation or exceeds the permitted use, you will need to obtain permission directly from the copyright holder. To view a copy of this license, visit http://creativecommons.org/licenses/by/4.0/.

REVIEWER COMMENTS

Reviewer #1 (Remarks to the Author): with expertise in Pin1 biology

Liu et al. proposed an interesting therapeutic approach by using DNA-barcoded micellular systems (DMS) encapsulating Pin1 inhibitor, in targeting Pin1 in Cancer associated fibroblast (CAFs). The authors further showed that CD8+ T lymphocytes engagement using integrated DNA aptamers (AptT). The bispecific antiCAFs-DMS-AptT system can bind to both CAF and CD8+ T lymphocytes and then suppressed Pin1 in CAF and induced CD8+ T lymphocytes infiltration, and then eradicate pancreatic cancer tumor in mouse model.

Overall, experimental approach to target Pin1 specifically in CAFs in PDAC is novel and the results are interesting. However, there are major concerns in the paper. Most notably, the authors used the commercially available compound AG17724 as a Pin1 inhibitor. However, I have not seen any original paper demonstrating that AG17724 is a Pin1-specific inhibitor, raising the question what is the cellular target for AG17724.

Major Comments

1) The authors used AG17724 as a Pin1 inhibitor in this study. What was the reason the authors selected AG17724 in this study? There is not original paper demonstrating that AG17724 is a Pin1 inhibitor. Since there are a number of Pin1 inhibitors published recently, the authors should repeat the experiments using other Pin1 inhibitors as well as Pin1 genetic inhibition to approve that they specifically target Pin1, not other targets.

2) Figure 2H-2K. The authors should describe the dose or concentration of AG17724 and AG17724 of anti-CAFs-DMS.

3) The bispecific antiCAFs-DMS-AptT system is very impressive. The authors showed that anti-CAFs-DMS-AptT can bind to both CAFs and CD8+ T lymphocytes. However, how anti-CAFs-DMS-AptT deliver CD8+T-cell into the tumor microenvironment in vivo? Because pancreatic cancer has an immunosuppressive tumor microenvironment (TME) which prevents CD8+ T cell infiltration by its abundant ECM and cytokines from cancer cells and CAFs.

4) According to the mechanisms of the anti-CAFs-DMS-AptT system, the anti-CAFs-DMS-AptT might suppress Pin1 in CD8+ T lymphocytes. However, the authors did not mention the Pin1 function in CD8+ T lymphocytes.

5) Figure 4E. How much concentration of AG17724 in anti-CAFs-DMS or anti-CAFs-DMS-AptT did the authors use in in vivo study? The authors should describe it.
To show the predominance, it would be better to use same concentration,

6) Figure 5A and 5B. The authors demonstrated that anti-CAFs-DMS-AptT depleted CAFs. However, the previous study presented that CAF depletion accelerated tumor growth in pancreatic cancer mouse model (Ref. Özdemir et al., Cancer Cell. 2014 Jun 16; 25(6):719-34). The authors should explain or discuss the difference between this study and the previous studies.

7) Figure 5G. The authors only use CD8 and CD3 antibodies to detect CD8+ T cells in flow cytometry. If the authors could remove dead cells and CD45 negative cells before gating CD8 and CD3, the result will be more accurate.

8) The authors described that “the anti-CAFs-DMS-AptT disrupts the desmoplastic and immunosuppressive TME” in discussion section (page 15), but they only investigate CAFs and CD8. To prove anti-CAFs-DMS-AptT disrupt the desmoplastic and immunosuppressive TME, the authors should investigate extracellular matrix (ECM) volume and other immune suppressive cells such as Tregs, MDSCs, Macrophages in TME.

9) The authors described that “This renders pancreatic tumors eradicable by cytotoxic T lymphocytes engagement’ in discussion section (page 15). However, it is still unclear whether CD8+ T lymphocytes contribute to tumor eradication in this study, and the authors did not investigate cytotoxic ability of T lymphocytes. The authors should investigate Perforin or Granzyme B in CD8+ T lymphocytes to prove their cytotoxic ability.

Minor Comment
1) Figure 4-6. To understand the in vivo experiment, it would be better to put tumor photos after the treatment. In addition, to prove CAF and CD8+ T cells present in TME, it would be better to perform immunohistochemistry staining or immunofluorescent staining.

Reviewer #2 (Remarks to the Author): with expertise in DNA barcoding - nanomedicine

The authors have described a nanomedicine-based system to deliver PIN1 inhibitors to CAFs, in order to treat cancer.

(1) In the introduction, please discuss and cite previously published DNA barcoding studies.

(2) There are a few grammatical / writing errors. Please re-read the manuscript carefully to correct them.

(3) The PDI of 0.05 is impressive, as is the stability for a week. Nice job!

(4) Figure 1c: If I'm interpreting this correctly, it would make sense to include a scrambled DNA control (i.e., one that does not bind the tagged sequence) in the studies.

(5) The claim of selectivity made in Figure 2 is important to the manuscript, given that the idea is to selectively deliver PIN1 to CAFs and not other cell types. However, the claim is not substantiated by the data. The claims need to be made with in vivo data, not by comparing different cell lines.

(6) The same is true for the data shown in Figure 3. Again, the claims of the dual system and its T cell mechanism need to be observed in vivo, not just in culture.

(7) Please make the data in Figure 4 easier to see / read.

(8) The data in Figure 5c,d are convincing.

(6)

Reviewer #3 (Remarks to the Author): with expertise in pancreatic cancer, TME, CAF

Authors investigate novel therapeutic approaches using bispecific drug delivery systems (DDS) for the treatment of pancreatic cancer. This study has the potential to address unmet clinical needs since only a small subset of patients with pancreatic cancer respond favorably to treatment despite the advances in cancer immunotherapies. This poor response is attributed in part to the immunosuppressive tumor microenvironment (TME), and therefore developing therapies capable to modulate the TME and facilitate T cell infiltration would be of high interest in the biomedical field.

In the present study, authors have developed a bispecific DNA-barcoded micellular system (DMS) which allows to deliver PIN1 inhibitor specifically into stromal cells and engage with cytotoxic T cells reducing tumor growth in vitro and in vivo. As a result, authors demonstrate that DMS systems

encapsulating PIN1 inhibitors are a feasible strategy to enhance the efficacy of this inhibitors in vivo. Interestingly, the screening of AG17724 in 3D and PDAC mice models pointed to an enhanced inhibition of the tumor progression by conjugating aptamers (Apt) to the DMS delivery system which favored T lymphocytes engagement to CAF and infiltration into the tumor compared to the unmodified DMS system.

Certainly, aptamer-based targeted DDS have attracted considerable attention because of their advantageous properties, nonetheless many aspects in this study remain unconclusive for which the manuscript requires major revisions to resolve the following questions:

• Results showed that bispecific anti-CAFs-DMS-Apt system encapsulating AG17724 can be used to selectively deliver PIN1 inhibitors into CAFs. However, A17724 target engagement and specific inhibition of PIN1 remains to be fully demonstrated by PPIase isomerase inhibition assays and/or RNA-seq analysis. On-target effects are not conclusive in Fig 2H, since the protein expression analysis of PIN1 and relative proteins in CAFs and Pan02 cells do not prove direct inhibition of PIN1 itself. In addition, closer relative proteins to PIN1 could have been chosen such as CDK1 for the analysis of downstream effects instead of AKT or NFkB which might be modulated as a result of cross-talks with other signaling pathways. In any case, the authors need to specify in Fig 2 that the antibody used in the western blot was P-T308-AKT accordingly to material and methods and add total AKT to the figure. Better quality in the band resolution might be achieved by using the Bolt system and/or Licor antibodies to improve the quantitative analysis of the western blot. On the same note, why is the protein expression of PIN1 decreased upon treatment (seen in fig 2J.) A17724 is claimed to be an inhibitor PIN1, supposedly reducing the activity of the protein. But also the level of protein is affected. How? Why is inhibition of PIN1 leading to lower expression of PIN1? This raises the question of PIN1 really is the target?

• Co-cultures of Pan02 spheroids with CAFs in ultra-low adherent 3D-speroid (Fig 3A-C) showed an enhanced effect of AG17724 on spheroid growth when delivered using the anti-CAFs-DMS system. Notably, despite the significant reduction in the spheroid area in treated vs control (PBS), spheroids remained larger than 2000 um3 after 7days of treatment suggesting that the 3D model failed to predict the effects in vivo. In addition, results suggest that the treatment using the anti-CAFs-DMS system exerts an antiproliferative effect in viable cells while senescence and hypoxic cells (nuclei of the spheroid) remains intact. To clarify this point, Live/dead staining would allow to have a better insight on the effect at the different layers of the spheroid and further investigate on potential cytotoxic effects triggered by AG17724 either on CAFs or cancer cells.

• Binding between T cells (MOHITO) and CAFs was validated in vitro (Fig 3). Further cytotoxic assays in co-culture with pancreatic cancer cells would demonstrate whether the T cells indeed exert a cytotoxic effect on cancer cells after treatment using the anti-CAFs-DMS-Apt system.

• The proposed DMS system is bispecific. The effect of AG17724 is only checked in cancer cells and CAFs. With the current approach is AG17724 also delivered to T cells. What effect is AG17724 having on T cells? Is PIN1 activity affected in the T cells, and in that case, a relevant question is if this is affecting T cell function?

• The consequences of PIN1 inhibition on CAFs still needs to be addressed in further detail. In recent years it has been shown that CAFs can be further subdivided in different subtypes. The CAFs studied here are SMA positive, indicating that they have a myofibroblastic CAF phenotype. Does PIN1 inhibition lead to a switch in phenotype to a more inflammatory phenotype? Or do the CAFs become quiescent? A quite direct way of answering these questions would be to sort out the CAFs after treatment and perform bulk RNA-seq and to compare the transcriptome profiles between saline and anti-CAFs-DMS treated CAFs.

• In fig 5 G-H the infiltrating T cells are studied. Further profiling of these infiltrating T cells would be easy to. Are these T cells active? Are they degranulated? Exhausted? Since the T cells are identified with flowcytometry, adding an additional flow-based T cell functionality assay would be very

informative.

• Since the approach is bispecific, it is important to disentangle the effects of each component one by one, and then in combination. How much does the AptT component (and the fact that more CD8+ cells infiltrate the tumor) contribute to in vivo effect? What is PIN1 inhibition add? By adding experiments with unloaded DMS (without AG17724) this could be investigated.

Minor concerns:

• Efficient cellular uptake was demonstrated by flow cytometry-based analysis in CAFs compared to pancreatic cancer cells (Fig 2). In addition, a significant reduction of CAFs (Fig 5) together with antitumor efficacy (Fig 6) was achieved after 18 days of treatment in subcutaneous and orthotopic pancreatic ductal adenocarcinoma (PDAC) mice models. Remarkably, at day 42 a significant reduction in tumor volume was observed using the anti-CAF-DMS-Apt system to deliver AG17724 without causing significant effects on body weight. Altogether, results suggest that the antibodies and aptamers conjugated to DMS might bind to targeted proteins with high affinity and specificity in vivo; nonetheless Fig 4A shows that there is a major fraction of antiCAF-DMS-Apt that accumulates in liver. Little is discussed about the repercussions that this observation might have in terms of toxicity, immunogenicity, long-term in vivo degradation, pharmacokinetics, or biocompatibility. Further safety and efficacy studies will be needed to discard toxicity of the DMS system itself.

• The need and the function of the DNA barcodes needs to be explained more in detail.

• The DMS system showed high drug encapsulation and stability for one week at room temperature. Nonetheless, it is plausible that a certain fraction of the drug might be released in a non-controlled manner due to issues related to the stability of DDS in vivo. Therefore, the stability of anti-CAFs-DMS-Apt systems needs to be tested in vivo; as well as potential effects on non-targeted cells over time.

• Grammar and proof-reading will be needed throughout the manuscript (i.e. lines 76, 88, 106, 130, 169, 246-49, 253-256, 290, 321, 332, 336-338, 354-355). Some missing references were also found (i.e. lines 208 and 218). Authors need to specify the number of technical and biological replicates in each experiment.

Taken it altogether, the authors conclude that bispecific aptamer-functionalized targeted DDS anti-CAFs-DMS-Apt system encapsulating AG17724 have shown its efficacy and potency to eradicate subcutaneous and orthotopic pancreatic ductal adenocarcinoma (PDAC). This conclusion might be considered a bit premature and overstated at this point since effective PIN1 inhibition in CAFs is not demonstrated yet, and issues regarding safety and efficacy in vivo need to be further addressed. Having said that, the anti-CAFs-DMS-Apt system is a promising therapeutic approach which offers the possibility to have a better insight into cell-level antitumor targeting therapy, as well as to better understand the biological functions of PIN1 in different cellular contexts including specific CAF subpopulations which contribute to the TME and are involved in the tumor progression.

Point-by-point response to the reviewers

Our responses:

Reviewer #1 (Remarks to the Author): with expertise in Pin1 biology

Liu et al. proposed an interesting therapeutic approach by using DNA-barcoded micellular systems (DMS) encapsulating Pin1 inhibitor, in targeting Pin1 in Cancer associated fibroblast (CAFs). The authors further showed that CD8+ T lymphocytes engagement using integrated DNA aptamers (AptT). The bispecific antiCAFs-DMS-AptT system can bind to both CAF and CD8+ T lymphocytes and then suppressed Pin1 in CAF and induced CD8+ T lymphocytes infiltration, and then eradicate pancreatic cancer tumor in mouse model.

Overall, experimental approach to target Pin1 specifically in CAFs in PDAC is novel and the results are interesting. However, there are major concerns in the paper. Most notably, the authors used the commercially available compound AG17724 as a Pin1 inhibitor. However, I have not seen any original paper demonstrating that AG17724 is a Pin1-specific inhibitor, raising the question what is the cellular target for AG17724.

We thank the reviewer for the comments and for his/her work.

Major Comments

(1) The authors used AG17724 as a Pin1 inhibitor in this study. What was the reason the authors selected AG17724 in this study? There is not original paper demonstrating that AG17724 is a Pin1 inhibitor. Since there are a number of Pin1 inhibitors published recently, the authors should repeat the experiments using other Pin1 inhibitors as well as Pin1 genetic inhibition to approve that they specifically target Pin1, not other targets.

REPONSE: Yes, AG17724 is a relatively new Pin1 inhibitor. AG17724 was screened out as a potent Pin1 inhibitor by Pfizer Global Research and Development, and the structure-based design of it had been clearly explained and published **[**Bioorg Med Chem Lett. 2009 Oct 1;19(19):5613-6. **|** Bioorg Med Chem Lett. 2010 Apr 1;20(7):2210-4. **|** Bioorg Med Chem Lett. 2014 Sep 1;24(17):4187-91.**]** Since then, AG17724 is validated and used as a structurally distinct Pin1 inhibitor in a few publications **[**Haematologica. 2021 Dec 1; 106(12): 3090–3099. **|** Haematologica. 2021 Dec 1; 106(12): 3030–3031. **|** Scientific Reports volume 6, Article number: 18822 (2016).**]**. Despite significant improvements of AG17724 in Pin1 inhibitor

affinity, it failed to show cellular effects on cancer cells due to poor cell permeability [Bioorg Med Chem Lett. 2014 Sep 1;24(17):4187-91.], it thus needs a delivery system to transport it into cells. Apart from these facts, the other important reason we used AG17724 is its hydrophobic property thus we can easily encapsulate it into the hydrophobic core of our DNA-barcoded micellular system (DMS).

To support the selective inhibition of Pin1 by AG17724, we now have performed parallel experiments with all-trans retinoic acid (ATRA), which is known as the more widely used Pin1 inhibitor [Nature Medicine 2015 May; 21(5): 457–466.], Juglone (which is another widely used Pin1 inhibitor) and shPin1 (Pin1 knockdown). The first experiment we performed is the inhibition of Pin1 catalytic activity, as measured by PPIase assay. The results show that AG17724 (Ki is 0.03 μM, which is similar as the range of previous publication [Bioorg Med Chem Lett. 2014 Sep 1;24(17):4187-91.]) has a more potent Pin1 inhibition efficiency than Juglone (Ki is above 10 μM) and ATRA (Ki is 1.99 μM) (**Fig#1-1A**).

To determine whether AG17724 specifically inhibits Pin1 activity in cells, we then compared its effects on the proliferation of Pin1 KD CAFs [shPin1 treatment, western blot shows the decrease of Pin1 protein levels in shPin1 treated CAFs (**Fig#1-1B**).] and wild-type CAFs. It showed that Pin1 KD CAFs were more resistant to AG17724 or antiCAFs-DMS than wild-type CAFs (**Fig#1-1C, 1D**).

To further support the thesis that AG17724 targets Pin1 in cells, we next carried out RT-qPCR to examine the effect of AG17724, antiCAFs-DMS, ATRA or Pin1 knockdown on abundance of a set of oncogenes and tumor suppressors whose expression is regulated by Pin1 [Nature Medicine 2015 May; 21(5): 457–466. | Nat Rev Mol Cell Biol. 2007;8:904–916. | Cell Res. 2014;24:1033–1049.]. On CAFs, it shows that AG17724, antiCAFs-DMS or ATRA can't reduce mRNA level of Pin1, which makes sense since they do not function in gene transcription. AG17724 itself can't change abundances of these transcripts, which could be again due to its poor cell permeability [Bioorg Med Chem Lett. 2014 Sep 1;24(17):4187-91.]. antiCAFs-DMS shows similar capacity as ATRA or shPin1 to effect transcriptions of these selected genes (**Fig#1-1E**), indicating that AG17724 delivered by antiCAFs-DMS indeed inhibits Pin1.

These results together can confrim that AG17724 targets and inhibits Pin1, but it firstly needs to be delivered into cells by for example our antiCAFs-DMS. We have added these results into the Figure S5 of our revised manuscript.

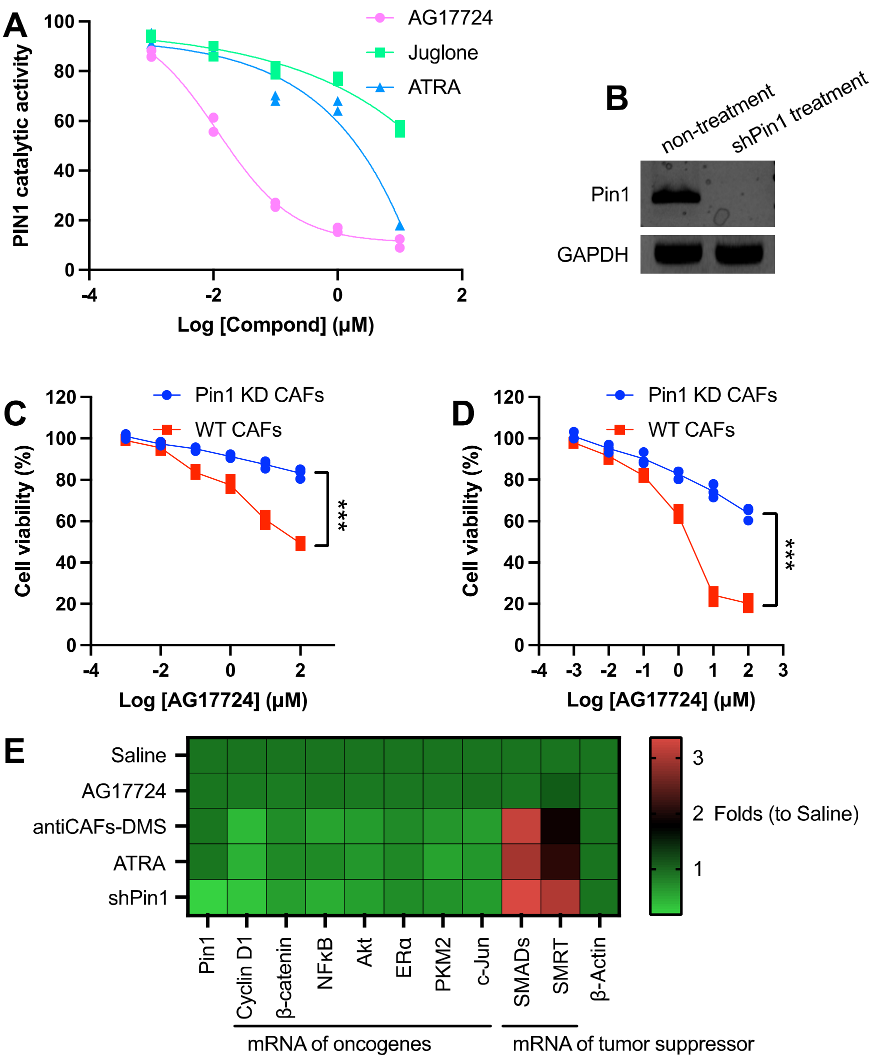

Fig#1-1. AG17724 targets Pin1. **(A)**, Inhibition of Pin1 catalytic activity by AG17724, Juglone or ATRA, as measured by PPIase assay (two experiments). **(B)**, Western blot showing the decrease in Pin1 protein levels in shPin1 treated cells. WT and Pin1 KD CAFs were treated with indicated concentrations of AG17724 **(C)** or corresponding antiCAFs-DMS **(D)** for 48 hours, followed by MTT cell growth assay with the readout absorbance measured by 570 nm (three experiments). ***$p < 0.001$. **(E)**, RT-qPCR analysis of gene expressions as indicated after 24-hour treatments with AG17724 (0.5 μM), antiCAFs-DMS (corresponds to 0.5 μM) and ATRA (25 μM). Mean values of three biological replicates is plotted in this heatmap.

(2) Figure 2H-2K. The authors should describe the dose or concentration of AG17724 and AG17724 of anti-CAFs-DMS.

REPONSE: Thanks for pointing this out, in the experiments of Figure 2H-2K,

concentration of AG17724 was 0.5 μM. We have indicated this information in our revised legend of Fig.2.

(3) The bispecific antiCAFs-DMS-AptT system is very impressive. The authors showed that anti-CAFs-DMS-AptT can bind to both CAFs and CD8+ T lymphocytes. However, how anti-CAFs-DMS-AptT deliver CD8+T-cell into the tumor microenvironment *in vivo*? Because pancreatic cancer has an immunosuppressive tumor microenvironment (TME) which prevents CD8+ T cell infiltration by its abundant ECM and cytokines from cancer cells and CAFs.

REPONSE: Thanks. How anti-CAFs-DMS-AptT works *in vivo* depends on the treatment stages. We can explain as following: ① In the beginning, anti-CAFs-DMS-AptT might not be able to bring CD8+ T lymphocytes into pancreatic tumor tissues, nevertheless, antiCAFs-DMS-AptT can still deliver AG17724 into CAFs and thus disrupt the immunosuppressive TME of pancreatic cancer, rendering it "reachable" and "reactive" by immune cells. Recent papers have also concluded that, on pancreatic ductal adenocarcinoma (PDAC), Pin1 inhibition in CAFs *via* chemical compounds can change the highly desmoplastic and immunosuppressive TME of PDAC, making PDAC eradicable by immunotherapy [Cell, 2021-09-02, Vol.184 (18), p.4753-4771.e27]. ② During the next treatment stage, anti-CAFs-DMS-AptT then can redirect CD8+ T lymphocytes into pancreatic tumor tissue, resulting in the eradication of cancer cells. We have added corresponding discussions about this question into our revised manuscript.

(4) According to the mechanisms of the anti-CAFs-DMS-AptT system, the anti-CAFs-DMS-AptT might suppress Pin1 in CD8+ T lymphocytes. However, the authors did not mention the Pin1 function in CD8+ T lymphocytes.

REPONSE: The aptamer recognizing $CD8^+$T lymphocyte in our system was discovered by Nataly Kacherovsky et al. **[**Nature Biomedical Engineering volume 3, pages783–795 (2019)**]** and used for the traceless isolation of pure $CD8^+$ T cells at low cost and high yield. antiCAFs-DMS-AptT thus is designed to bind T cells but not get internalized by them. We now have added corresponding experimental results to prove this: We incubated $CD8^+$ T cells with antiCAFs-DMS-AptT-Cy5 for different time point and measured the Cy5 signal from T cells before and after DNase I treatment (DNase I treatment can degrade DNA aptamers bridging

antiCAFs-DMS-AptT-Cy5 to T cells). It (**Fig#1-2**) shows that DNase I can almost completely decrease Cy5 signal from high level to the level of PBS treatment (control), indicating that antiCAFs-DMS-AptT mostly stayed on cell surface of T cells rather than in the cells.

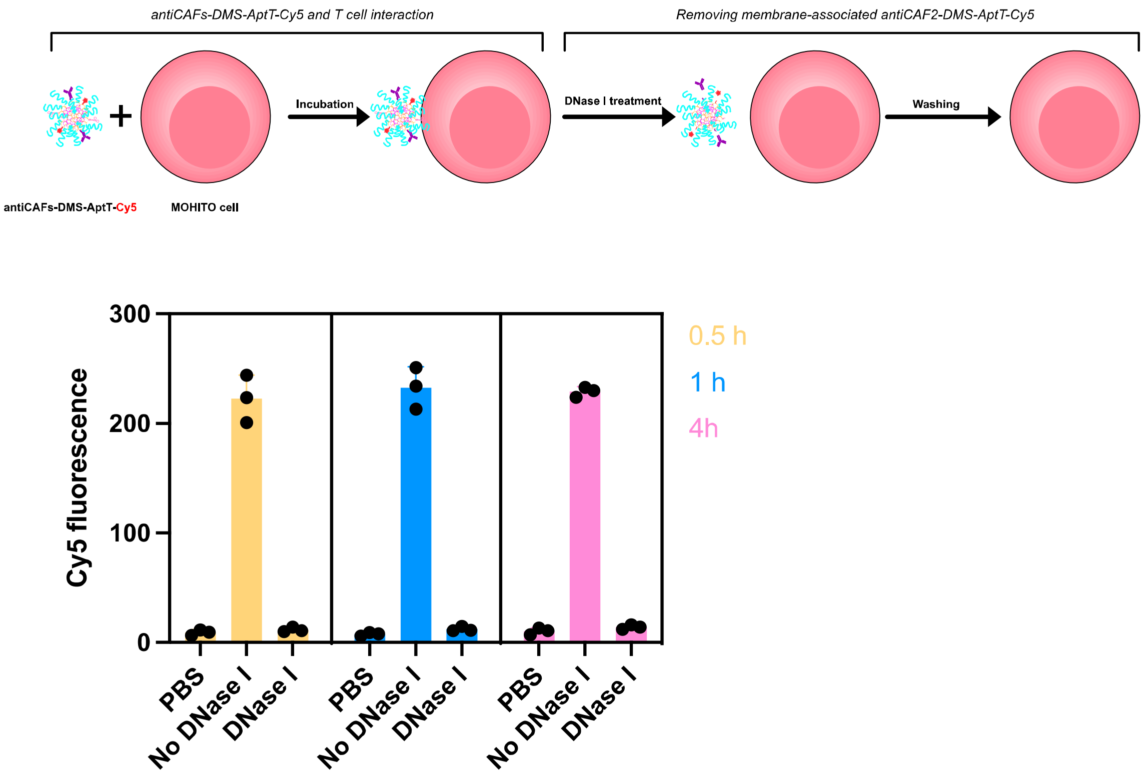

Fig#1-2. DNase I treatment workflow (Top chart) to study the association of antiCAFs-DMS-AptT-Cy5 with CD8$^+$ T cells. After different time points as indicated, Cy5 signals were detected and plotted (three experiments).

We also investigated viability of CD8$^+$ T cells (isolated from mouse spleens) after incubation with AG17724, DMS, antiCAFs-DMS or antiCAFs-DMS-AptT for 48 hours. It shows that, after being encapsulated into DMS, the toxicity of AG17724 to T cells decreased (**Fig#1-3A**). We can attribute this to the very low uptake of our DMS systems by T cells.

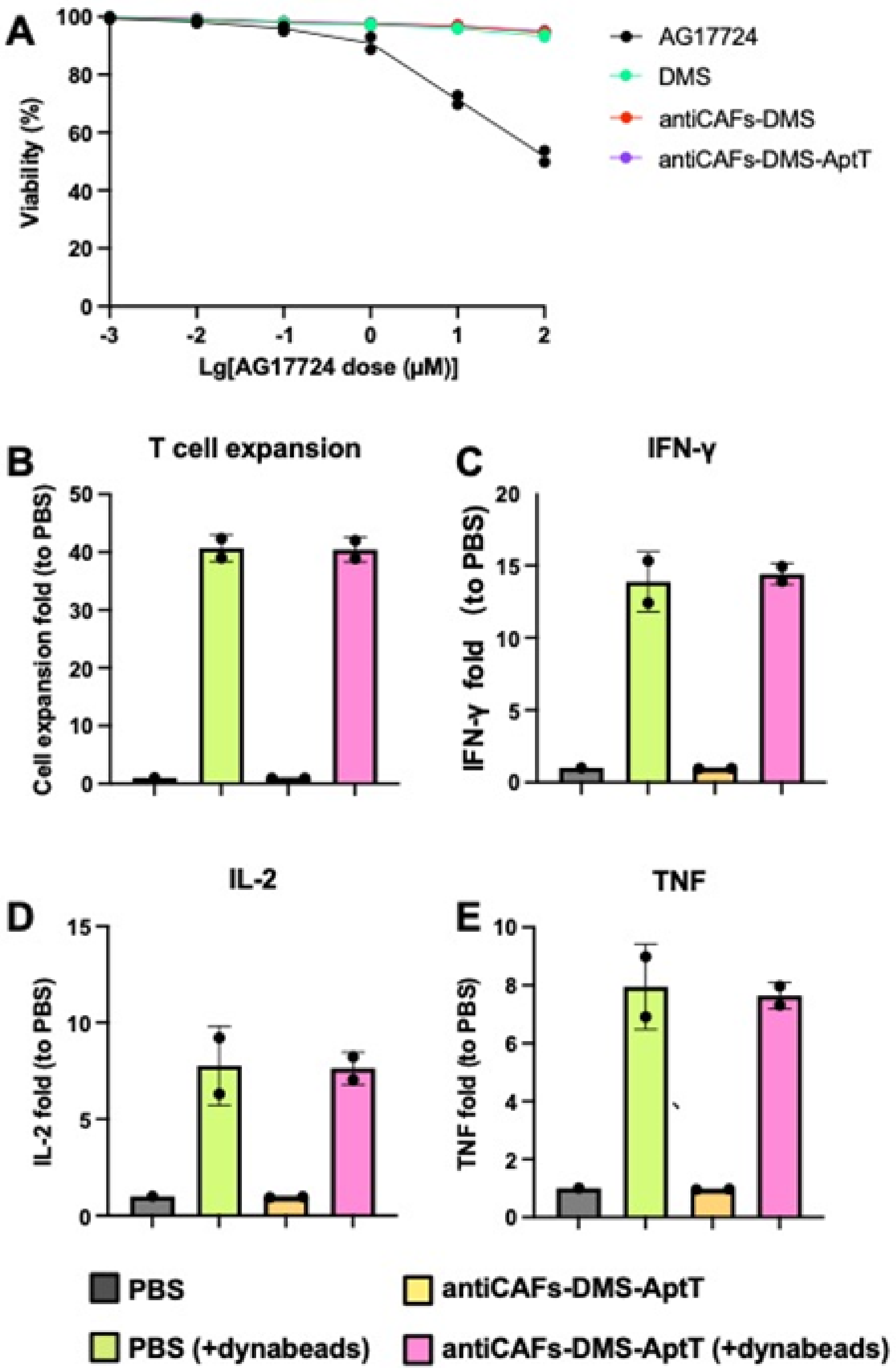

Fig#1-3. CD8⁺ T cell viability and function assays. **(A)**, Viability of CD8⁺ T cells with different treatments as indicated. **(B)** T cell expansion, **(C)** IFN-γ, **(D)** IL-2 and **(E)** TNF measurements after firstly treating the cells with/without antiCAFs-DMS-AptT for 48 hours then with/without antiCD3&antiCD28 Dynabeads for 6 days. Data points are plotted from two experiments.

We further tested $CD8^+$ T cell functions after treating them with 0.5-μM AG17724 corresponding antiCAFs-DMS-AptT, *via* measuring T cell expansion, IFN-γ, IL-2 and TNF. Our results (**Fig#1-3B, 3C, 3D, 3E**) show that antiCAFs-DMS-AptT does not affect functions of T cells on these four aspects.

(5) Figure 4E. How much concentration of AG17724 in anti-CAFs-DMS or anti-CAFs-DMS-AptT did the authors use in in vivo study? The authors should describe it. To show the predominance, it would be better to use same concentration.
REPONSE: For our *in vivo* study, the amount of AG17724 we used was 10 mg/kg. The amount of anti-CAFs-DMS or anti-CAFs-DMS-AptT corresponded to 10 mg/kg of AG17724. We have made it more clear in our revised manuscript.

(6) Figure 5A and 5B. The authors demonstrated that anti-CAFs-DMS-AptT depleted CAFs. However, the previous study presented that CAF depletion accelerated tumor growth in pancreatic cancer mouse model (Ref. Özdemir et al., Cancer Cell. 2014 Jun 16; 25(6):719-34). The authors should explain or discuss the difference between this study and the previous studies.

REPONSE: Thanks for letting us know this work. Yeah, this paper concluded that depletion of carcinoma-associated fibroblasts and fibrosis induces immunosuppression and accelerates pancreas cancer. We think the main difference here is that our bispecific delivery system could bring $CD8^+$ T cells to the tumor environment, thus changing the environment of only CAF depletion and initiating anti-tumor immune response.

(7) Figure 5G. The authors only use CD8 and CD3 antibodies to detect CD8+ T cells in flow cytometry. If the authors could remove dead cells and CD45 negative cells before gating CD8 and CD3, the result will be more accurate.
REPONSE: Thanks for your insightful comments. The process how we purified CD8+ T cells actually included the removal of dead cells and CD45 negative cells. We have indicated these more clearly in our revised manuscript.

(8) The authors described that " the anti-CAFs-DMS-AptT disrupts the desmoplastic and immunosuppressive TME" in discussion section (page 15), but they only investigate CAFs and CD8. To prove anti-CAFs-DMS-AptT disrupt

the desmoplastic and immunosuppressive TME, the authors should investigate extracellular matrix (ECM) volume and other immune suppressive cells such as Tregs, MDSCs, Macrophages in TME.

REPONSE: Thanks. We here have detected collagen, which is the most abundant ECM proteins in the PDAC ECM (comprising over 80% of all ECM mass) in tumor lysates using Total Collagen Assay Kit (Perchlorate-Free) (ab222942). It shows (**Fig#1-4**) that antiCAFs-DMS and antiCAFs-DMS-AptT treatments indeed significantly decreased the collagen abundance in pancreatic tumor [we checked the orthotopic PDAC at the 25th day of our treatment schedule (Figure 6A)].

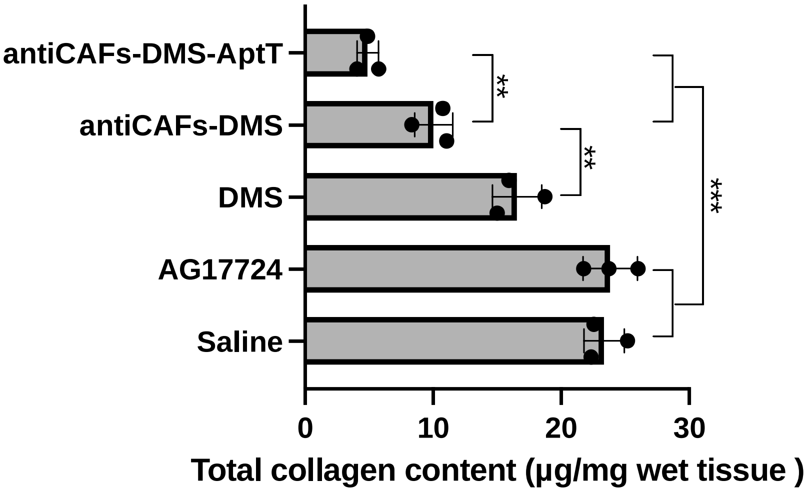

Fig#1-4. Total collagen content of tumors detected by Collagen kits. Experiments were repeated three times. Values are expressed as mean ± SD (n = 5, each dot represents a tumor sample)

Instead of analyzing other immune suppressive cells, we analyzed if tumor infiltrating CD3$^+$&CD8$^+$ T cells are active, suppressed or exhausted *via* profiling their expressions of PD-1, TIM-3 and LAG-3 over 3 days of activation with Dynabeads CD3/CD28. Pre-exhausted CD3+& CD8+ T cells [isolated from mouse spleens, stimulated with Dynabeads CD3/CD28 (1:1 bead-to-cell ratio) and re-stimulated every 2-3 days (5 stimulations total) prior to seeding.] were used as the control. From the result (**Fig#1-5**), we can see that CD3$^+$&CD8$^+$ T cells isolated from the tumor expressed low levels of exhaustion-related receptors. When we stimulated these cells with different concentrations Dynabeads CD3/CD28 for 3 days, we can see the increasing expression of these receptors, indicating the exhaustion progression of these cells. These data can tell us that these infiltrating

T cells in tumor are active rather than suppressed or exhausted, or at least not fully exhausted.

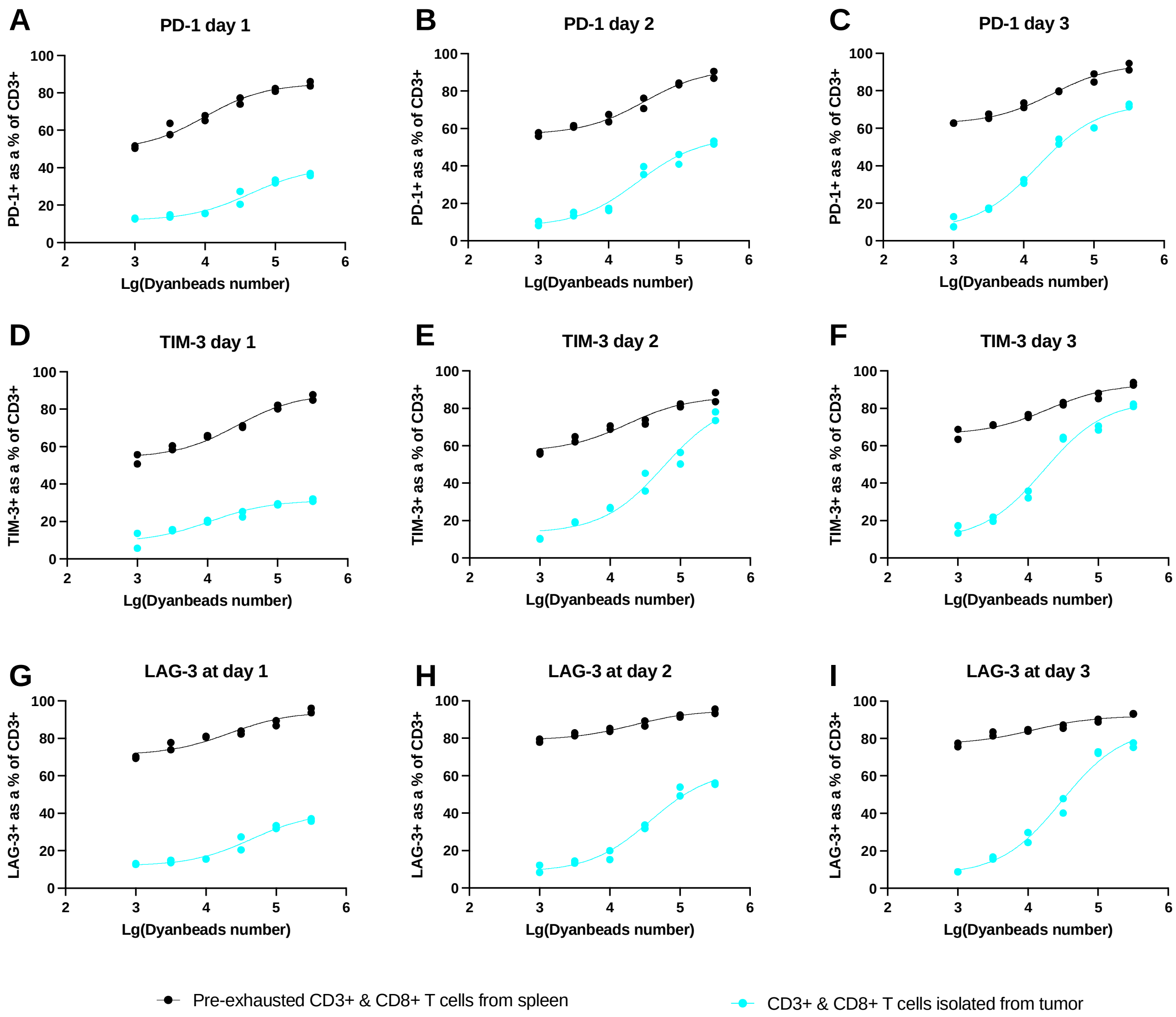

Fig#1-5. T cell exhaustion assay *via* measuring the expressions of PD-1(A-C), TIM-3 (D-F) and LAG-3 (G-I). At the 25th day of antiCAFs-DMS-AptT treatment, CD3+ & CD8+ T cells were isolated from tumor. These cells or pre-exhausted CD3+ & CD8+ T cells were seeded at 200K/well. Exhausted T cells had been repeatedly stimulated (5 times total, every 2-3 days) with Dynabeads CD3/CD28 (1:1 bead-to-cell ratio). In-well, activation was induced with Dynabeads CD3/CD28. Every 24h, 10uL samples were analysed using the iQue® Mouse T Cell Kit. Data points are plotted as n = 2 mice.

(9) The authors did not investigate cytotoxic ability of T lymphocytes. The authors should investigate Perforin or Granzyme B in CD8+ T lymphocytes to prove their cytotoxic ability.

REPONSE: Thanks and we now have investigated Granzyme B in CD8+ T cells *via* intracellular immunofluorescent staining with flow cytometric analysis. After we isolated CD8+ T cells (*via* FITC-antiCD8 and PE-antiCD3) from tumors treated with antiCAFs-DMS-AptT, cells were lysed to cell lysates and then analyzed using Mouse Granzyme B ELISA Kit PicoKine™. CD8 T cells isolated from healthy mouse spleen were used as control. We can see almost 7 times more Granzyme B in CD8+ T cells isolated from tumor (treated with antiCAFs-DMS-AptT) than the control (**Fig#1-6**), indicating that these T cells are cytotoxic. We have added this to our revised manuscript.

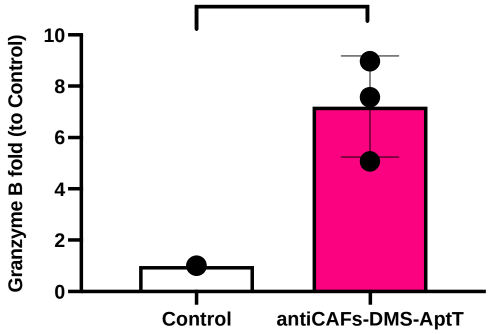

Fig#1-6. Intracellular Granzyme B analysis in CD8+ T cells (Control: CD8+ T cells isolated from spleen; antiCAFs-DMS-AptT: CD8+ T cells isolated from tumors treated with antiCAFs-DMS-AptT) using ELISA. Data points are plotted as n = 3 mice. ***$p < 0.001$.

Minor Comment

1) Figure 4-6. To understand the in vivo experiment, it would be better to put tumor photos after the treatment. In addition, to prove CAF and CD8+ T cells present in TME, it would be better to perform immunohistochemistry staining or immunofluorescent staining.

REPONSE: Thanks and we have now added corresponding results (**Fig#1-7**).

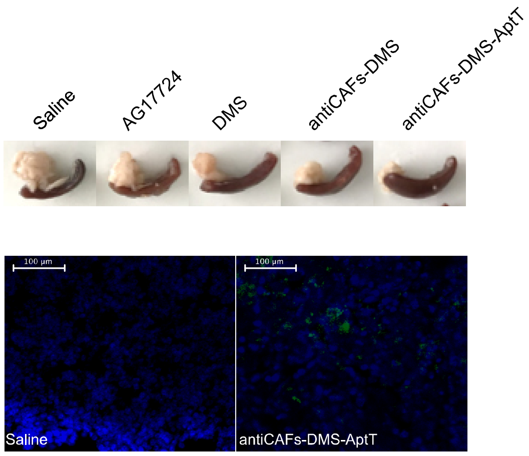

Fig#1-7. Representative tumor images from different treatment and fluorescent CD8 staining on tissue slices of tumors (tumors were collected at the 30th of our treatment schedule) treated with saline and antiCAFs-DMS-AptT, respectively.

Reviewer #2 (Remarks to the Author): with expertise in DNA barcoding – nanomedicine

The authors have described a nanomedicine-based system to deliver PIN1 inhibitors to CAFs, in order to treat cancer.

We thank the reviewer for the comments and for his/her work. Our revisions corresponding to your comments are colored in GREEN in the updated manuscript.

(1) In the introduction, please discuss and cite previously published DNA barcoding studies.

REPONSE: We have now cited corresponding studies **[**Nature Communications volume 7, Article number: 13325 (2016) **|** Proc Natl Acad Sci U S A. 2017 Feb 21;114(8):2060-2065. **|** Nature Nanotechnology volume 16, pages214–223 (2021)**]** and discussed them in the introduction of our revised manuscript.

(2) There are a few grammatical / writing errors. Please re-read the manuscript carefully to correct them.

REPONSE: Thanks and now we have carefully corrected these errors in our revised manuscript.

(3) The PDI of 0.05 is impressive, as is the stability for a week. Nice job!

REPONSE: Thanks!

(4) Figure 1c: If I'm interpreting this correctly, it would make sense to include a scrambled DNA control (i.e., one that does not bind the tagged sequence) in the studies.

REPONSE: Thanks for the suggestion. We have added the scrambled DNA control in our revised Figure 1C (also as in **Fig#2-1**). We ran three samples on 2% agarose gel: ①, sample **I**: DMS; ②, sample **II**: DMS incubated with Cy5-DNA for barcode1, Cy3.5-DNA for barcode2 and Alexa488-DNA for barcode3; ③, sample **III**: DMS incubated with Cy5-scrambled DNA, Cy3.5-scrambled DNA and Alexa488-scrambled DNA. As we can see under different imaging channel, scrambled DNA sequences do not bind to DMS, indicating that there are no observable interactions between DMS and random DNA sequences.

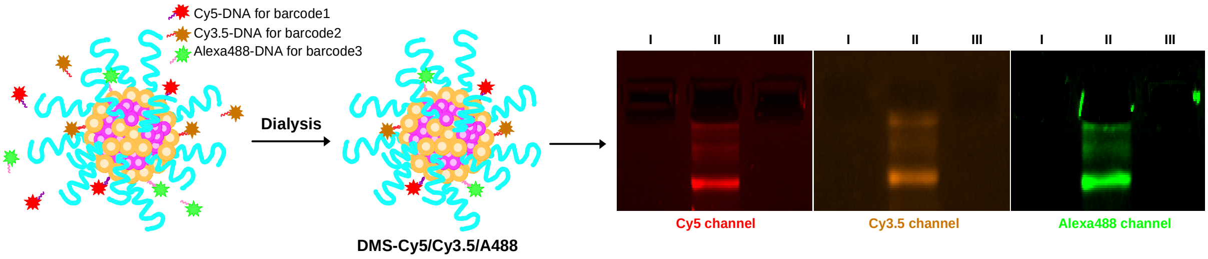

Fig#2-1. The accessibility and specificity of DNA barcodes on DMS.

(5) The claim of selectivity made in Figure 2 is important to the manuscript, given that the idea is to selectively deliver PIN1 to CAFs and not other cell types. However, the claim is not substantiated by the data. The claims need to be made with *in vivo* data, not by comparing different cell lines.

REPONSE: Thanks. In our *in vivo* experiments, we collected tumors at the 25th day for fluorescence-activated cell sorting analysis. We sorted cells into two different groups. Group#1: CAFs and Group#2: non-CAFs cells. We firstly analyzed PIN1 inhibition between CAFs and non-CAFs cells *via* western blot. We can clearly see that: ①, On CAFs (**Fig#2-2A, 2B**), antiCAFs-DMS can significantly inhibit Pin1 and its higly related proteins whereas free AG17724 can't; ②, On non-CAFs cells (**Fig#2-2C, 2D**), however, there are no statistic differences between free AG17724 treatment and antiCAFs-DMS treatment. This could indrectly refect on the *in vivo* cellular selectivity of antiCAFs-DMS. We have integrated these results into our revised Figure 5.

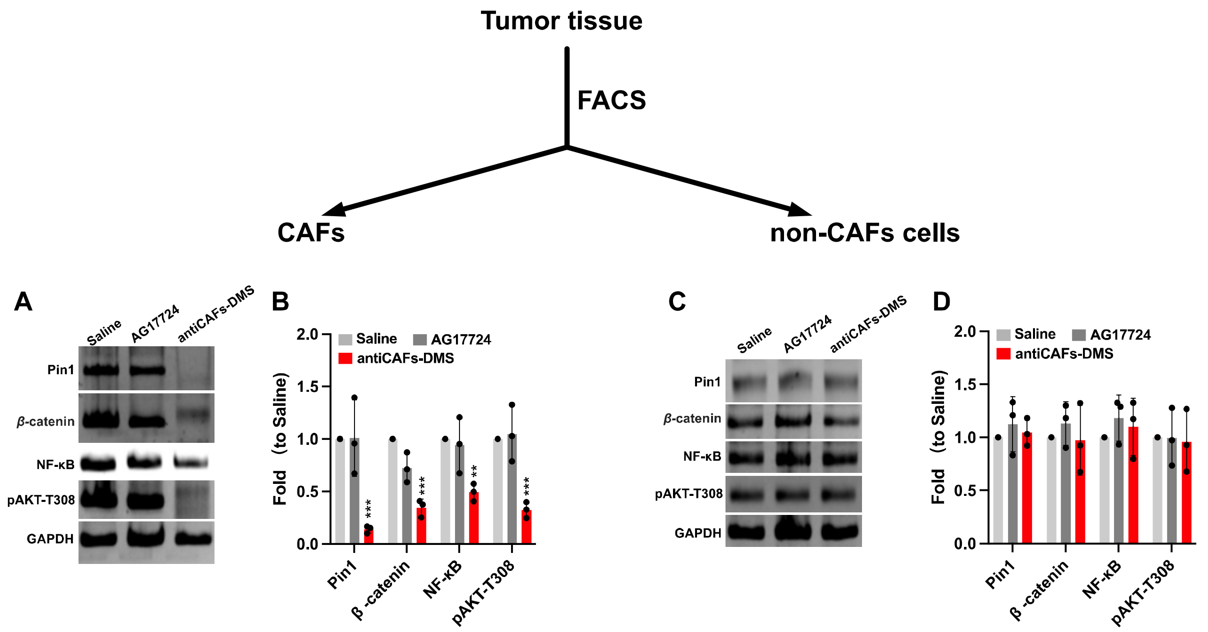

Fig#2-2. Pin1 inhibition analysis on CAFs and non-CAFs cells of tumor.

To more directly show the *in vivo* selectivity of antiCAFs-DMS on CAFs, we performed qPCR to comparatively quantify DNA barcodes (Note: our DMS system contains three different DNA barcodes, we thus can quantify these barcodes in cells *via* qPCR) in CAFs and non-CAFs cells of tumor. Our results show that much more DNA barcodes (around 22 folds) were detected in CAFs than in non-CAFs cells (**Fig#2-3**), directly indicating that antiCAFs-DMS has its cellular selectivity *in vivo* towards CAFs.

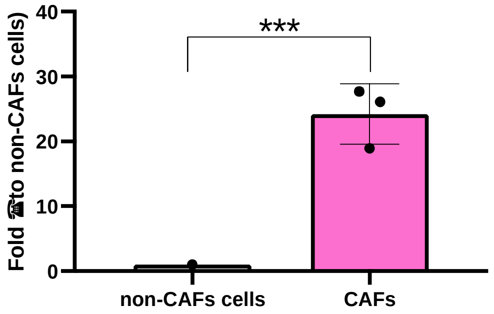

Fig#2-3. qPCR analysis to compare DNA barcode1 in CAFs and non-CAFs cells of tumor. Each dot stands for one mouse.

(6) The same is true for the data shown in Figure 3. Again, the claims of the dual system and its T cell mechanism need to be observed *in vivo*, not just in culture.

REPONSE: Thanks. We determined this *via* FACS-based quantification of $CD8^+$ T cells in tumors. Our data shows that antiCAFs-DMS does not effectively redirect

CD8⁺ T cells to tumors. Being different from antiCAFs-DMS, antiCAFs-DMS-AptT significantly increased intra-tumoral CD8⁺ T cells by around 15 folds (**Fig#2-4**). This can indicate that antiCAFs-DMS-AptT does have the bispecific function.

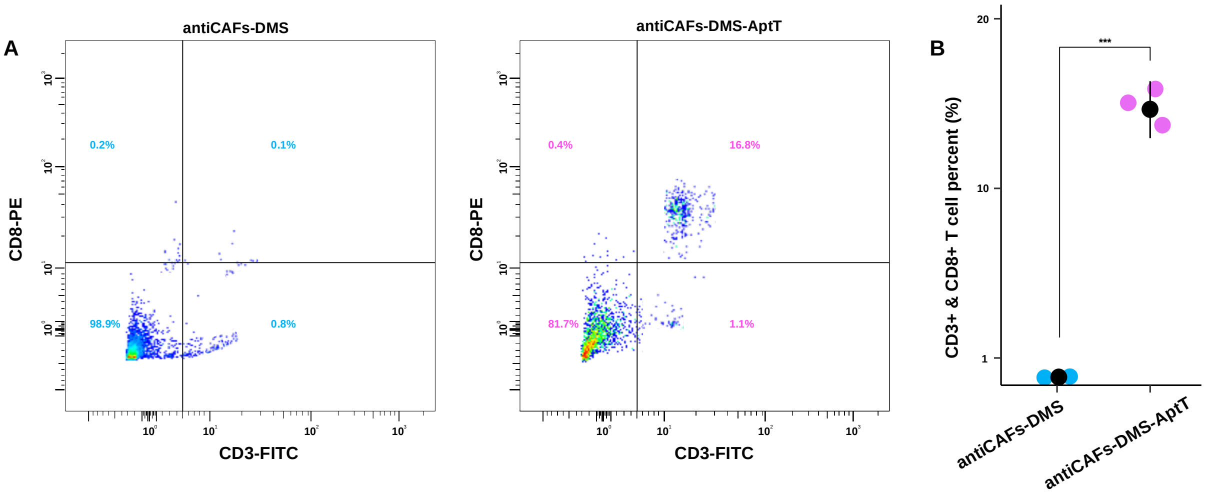

Fig#2-4. Quantitative analysis, *via* cell sorting and counting, of CD8⁺ T lymphocytes in tumors from mice treated with antiCAFs-DMS or antiCAFs-DMS-AptT.

(7) Please make the data in Figure 4 easier to see / read.

REPONSE: We have done this by increasing the font size of Figure 4. Thanks.

(8) The data in Figure 5c,d are convincing.

REPONSE: Thanks.

Reviewer #3 (Remarks to the Author): with expertise in pancreatic cancer, TME, CAF

Authors investigate novel therapeutic approaches using bispecific drug delivery systems (DDS) for the treatment of pancreatic cancer. This study has the potential to address unmet clinical needs since only a small subset of patients with pancreatic cancer respond favorably to treatment despite the advances in cancer immunotherapies. This poor response is attributed in part to the immunosuppressive tumor microenvironment (TME), and therefore developing therapies capable to modulate the TME and facilitate T cell infiltration would be of high interest in the biomedical field.

In the present study, authors have developed a bispecific DNA-barcoded micellular system (DMS) which allows to deliver PIN1 inhibitor specifically into stromal cells and engage with cytotoxic T cells reducing tumor growth in vitro and in vivo. As a result, authors demonstrate that DMS systems encapsulating PIN1 inhibitors are a feasible strategy to enhance the efficacy of this inhibitors in vivo. Interestingly, the screening of AG17724 in 3D and PDAC mice models pointed to an enhanced inhibition of the tumor progression by conjugating aptamers (Apt) to the DMS delivery system which favored T lymphocytes engagement to CAF and infiltration into the tumor compared to the unmodified DMS system.

Certainly, aptamer-based targeted DDS have attracted considerable attention because of their advantageous properties, nonetheless many aspects in this study remain unconclusive for which the manuscript requires major revisions to resolve the following questions:

We thank the reviewer for the comments and for his/her work. Our revisions corresponding to your comments are colored in PURPLE in the updated manuscript.

(1) Results showed that bispecific anti-CAFs-DMS-Apt system encapsulating AG17724 can be used to selectively deliver PIN1 inhibitors into CAFs. However, A17724 target engagement and specific inhibition of PIN1 remains to be fully demonstrated by PPIase isomerase inhibition assays and/or RNA-seq analysis. On-target effects are not conclusive in Fig 2H, since the protein expression analysis of PIN1 and relative proteins in CAFs and Pan02 cells do not prove direct inhibition of PIN1 itself. In addition, closer relative proteins to PIN1 could have been chosen such as CDK1 for the analysis of downstream effects instead of AKT or NFkB which might be modulated as a result of cross-talks with other signaling pathways. In any case, the authors need to specify in Fig 2 that the antibody used in the western blot was P-T308-AKT accordingly to material and methods and add total AKT to the figure. Better quality in the

band resolution might be achieved by using the Bolt system and/or Licor antibodies to improve the quantitative analysis of the western blot. On the same note, why is the protein expression of PIN1 decreased upon treatment (seen in fig 2J.) A17724 is claimed to be an inhibitor PIN1, supposedly reducing the activity of the protein. But also the level of protein is affected. How? Why is inhibition of PIN1 leading to lower expression of PIN1? This raises the question of PIN1 really is the target?

REPONSE: Thanks for your suggestions. We then assessed the ability of AG17724 [also used all-trans retinoic acid (ATRA), which is known as the more widely used Pin1 inhibitor, and Juglone, which is another widely used Pin1 inhibitor, as controls] to inhibit Pin1 enzymatic activity using a chymotrypsin-coupled PPIase assay. In this spectrophotometric assay, the peptidic substrate (Succ-Ala-p.Ser-Pro-Phe-pNA) is only cleaved by chymotrypsin when the peptidyl-prolyl bond is in the trans conformation. The results show that AG17724 (Ki is 0.03 μM, which is similar as the range of previous publication [Bioorg Med Chem Lett. 2014 Sep 1;24(17):4187-91.]) has a more potent Pin1 inhibition efficiency than Juglone (Ki is above 10 μM) and ATRA (Ki is 1.99 μM) (**Fig#3-1**). This result has been added into the fig.S5 of our revised manuscript.

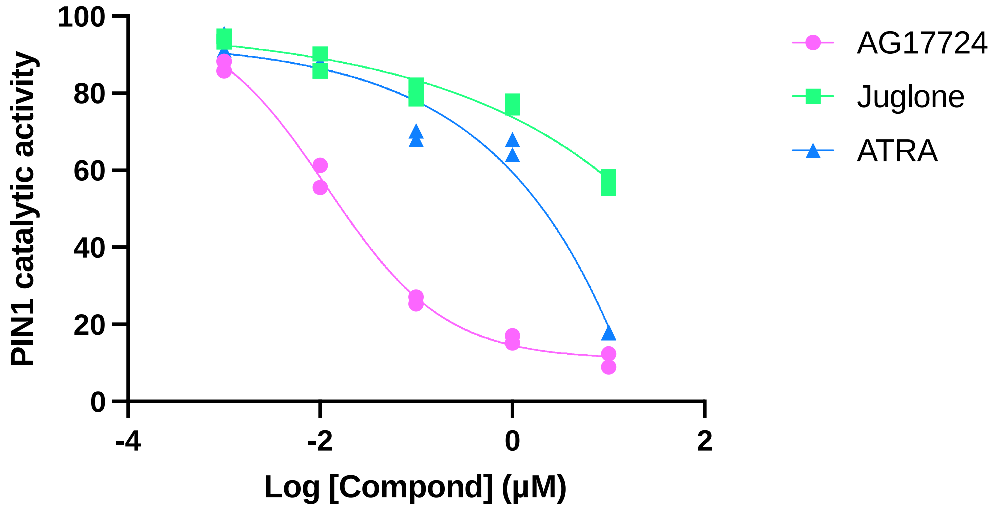

Fig#3-1. PPIase assay results for AG17724, Juglone and ATRA after a 12-h incubation with PIN1. Data points are plotted as n = 2 independent experiments, with each experiment having n = 1 independent samples.

We also added western blot results measuring the cellular amounts of both CDK1 and phosphor-CDK1 Y15, and result shows that CDK1 level is quite similar among different treatments, whereas the phosphor-CDK1 Y15 is elevated by Pin1

inhibition (**Fig#3-2**). This means that Pin1 inhibition caused by AG17724 then affects expression of downstream Pin1 substrates.

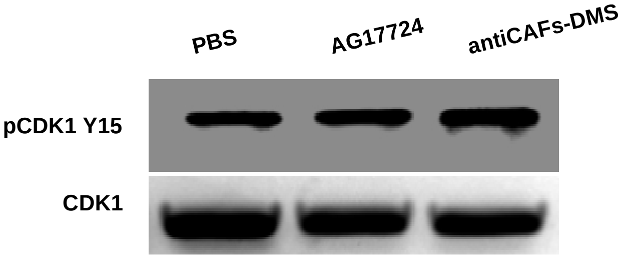

Fig#3-2. Western blotting of pCDK1-Y15 and CDK1 after 24-hour treatment with 0.1 μM of AG17724 or antiCAFs-DMS.

Yes, in our original Fig.2, they are the bands of pAKT-T308. We have labeled it correctly now. We also measured the total AKT *via* western blot, and it shows that total AKT among different treatments is very close (**Fig#3-3**).

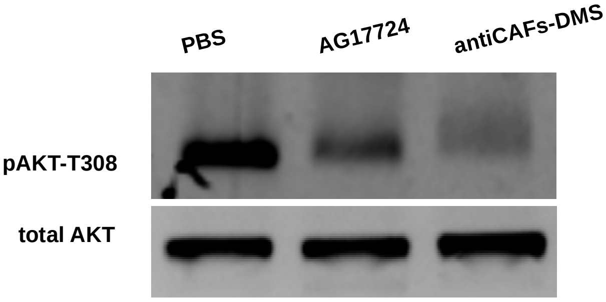

Fig#3-3. Western blotting of pAKT-T308 and total AKT after 24-hour treatment with 0.1 μM of AG17724 or antiCAFs-DMS.

We have added these new results into our revised Figure 2.

Why we got less Pin1 from AG17724 treatment? Thanks for asking this question, and we might speculate that AG17724 works not only *by* inhibiting Pin1 but also promoting its degradation afterwards. There might be a good example about this: as the more widely tested Pin1 inhibitor, retinoic acid (RA) binds, inhibits, and induces Pin1 degradation **[**Nature Communications volume 9, Article number: 3069 (2018) | Nature Medicine. 2015 May; 21(5): 457–466.**]**. With the treatment of ATRA, less Pin1 showed up on western blotting bands. A previous study has compared the downstream effects of Pin1 inhibition caused by RA or AG17724, and showed that RA and AG17724 caused similar pharmacological effects **[**Haematologica. 2021 Dec 1;106(12):3090-3099.**]**.

(2) Co-cultures of Pan02 spheroids with CAFs in ultra-low adherent 3D-speroid (Fig 3A-C) showed an enhanced effect of AG17724 on spheroid growth when

delivered using the anti-CAFs-DMS system. Notably, despite the significant reduction in the spheroid area in treated vs control (PBS), spheroids remained larger than 2000 um3 after 7days of treatment suggesting that the 3D model failed to predict the effects in vivo. In addition, results suggest that the treatment using the anti-CAFs-DMS system exerts an antiproliferative effect in viable cells while senescence and hypoxic cells (nuclei of the spheroid) remains intact. To clarify this point, Live/dead staining would allow to have a better insight on the effect at the different layers of the spheroid and further investigate on potential cytotoxic effects triggered by AG17724 either on CAFs or cancer cells.

REPONSE: Thanks. Please let us explain how we interpret it: Firstly, as described in our METHOD section, we pre-incubated CAFs with AG17724 or antiCAFs-DMS, these CAFs were then collected and seeded on the top of Matrigel which had Pan02 pancreatic cancer spheroids inside for indirect co-culture. During the 7-day coculture, we recorded recording organoid size. This workflow means that there is no direct adding of AG17724 or antiCAFs-DMS to the medium of co-culture. The purpose is to check whether selective PIN1 inhibition in CAFs affects their ability to act on pancreatic cancer spheroids rather than the direct toxicity of AG17724 or antiCAFs-DMS to the spheroids. Secondly, 0.5 μM was the concentration of AG17742 or antiCAFs-DMS for the pre-treatment of CAFs, and it was not supposed to cause high cyto-toxicities to either Pan02 cells or CAFs. Our cytotoxicity data in Figure 2E and 2F clearly shows that: ①, more than 80% of Pan02 cells and CAFs can survive after 48-hour treatments with 0.5-μM free AG17724. ②, more than 90% Pan02 cells and around 60% of CAFs survive after 48-hour treatments with antiCAF-DMS (corresponding to 0.5-μM AG17724), respectively. Based on these, we were not expecting the experiment of indirect CAFs and Pan02 spheroids co-culture would obviously decrease the original size of pre-formed Pan02 pancreatic cancer spheroids. It also shows that antiCAFs-DMS can stabilize sizes of Pan02 spheroids, which is already very good. Thirdly, our *in vivo* data in Figure 4 shows that antiCAFs-DMS can only slow down the growth of the subcutaneous PDAC tumor rather than eventually inhibit it. With these three aspects into consideration, we interpret that this experiment can properly predict the corresponding effects *in vivo*.

We now have added the experiment of live/dead staining on Pan02 spheroids at the 5th day of indirect co-culture. It shows (**Fig#3-4**) that CAFs pre-incubated with antiCAFs-DMS cause much more dead cells within Pan02 spheroids. We added this result into our revised supplementary information as fig.S6.

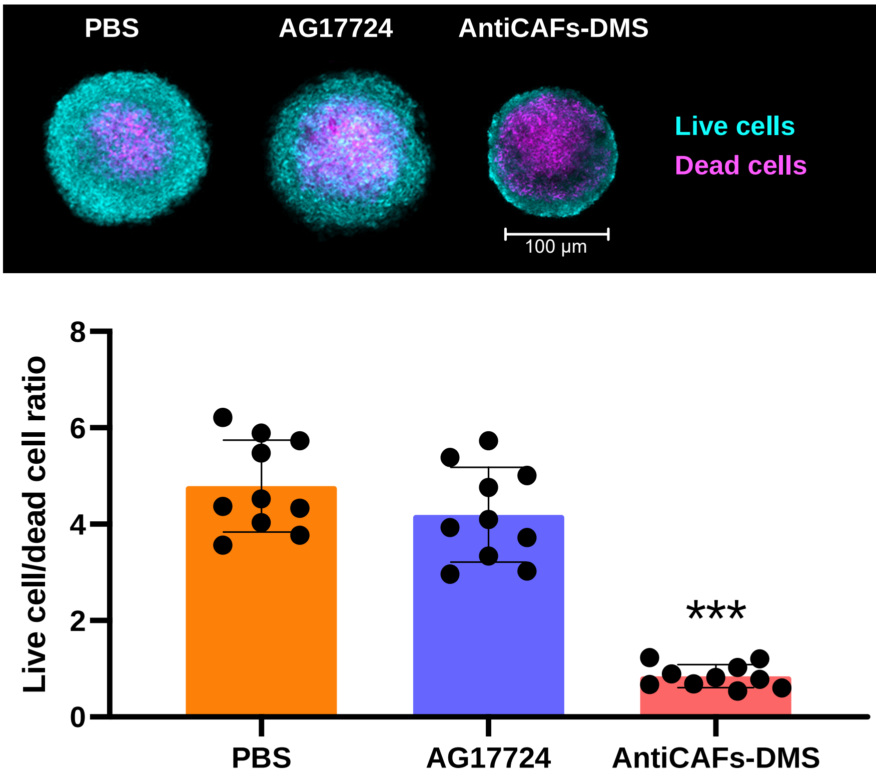

Fig#3-4. Treated spheroids were stained using the viability/cytotoxicity assay kit to determine live and dead cells. Images were generated using maximum projection from a Z-stack of seven images 15 µm apart. Bar plot at the bottom shows the live cell to dead cell ratios of spheroids (n = 10).

(3) Binding between T cells (MOHITO) and CAFs was validated in vitro (Fig 3). Further cytotoxic assays in co-culture with pancreatic cancer cells would demonstrate whether the T cells indeed exert a cytotoxic effect on cancer cells after treatment using the anti-CAFs-DMS-Apt system.

REPONSE: Thanks and we now have finished this coculture experiments: **First step:** we labeled luciferase cancer cell (Pan02_Luc) and CAFs with the membrane dye PKH-26 (Sigma–Aldrich), and co-cultured them at the ratio of 2/1 (Pan02_Luc/CAFs) for 12 hours. Only Pan02_Luc or only CAFs was cultured as controls; **Second step:** we added $CD8^+$ T cells as effector cells at an effector-to-target (target means Pan02_Luc and CAFs together) ratio of 3:1 in the presence of anti-CAFs-AptT (corresponds to 0.5 µM of AG17724) or empty antiCAFs-AptT (no AG17724 encapsulation) for 24-hour culture; **Third step:** The extent of cell lysis in the target cells was determined by staining with a LIVE/DEAD Fixable Violet Dead Cell Stain Kit (Life Technologies) and measured by the number of dead cells in the labeled target cell population. The viability of Pan02_Luc cells was analyzed via method of bioluminescence.

Our results show that: ① On only CAFs, antiCAFs-DMS-AptT can potently mediate CD8+ T cell to lyse CAFs (**Fig#3-5A**); on only Pan02_Luc cells, antiCAFs-DMS-AptT however can't really mediate cytotoxicity of T cells (**Fig#3-5A, 5B**). ② On CAFs and Pan02_Luc coculture, antiCAFs-DMS-AptT can significantly induce T cell-mediated lysis of Pan02_Luc cells (**Fig#3-5A, 5B**). ③ On CAFs and Pan02_Luc coculture, empty antiCAFs-DMS-AptT (without AG17724 encapsulation) had similar effects as antiCAFs-DMS-AptT (**Fig#3-5A, 5B**). These results together indicate that antiCAFs-DMS-AptT can work as bispecific antibody to engage T cells to CAFs, resulting in lysis of CAFs. Meanwhile, activated T cells can further exert cytotoxic effects on pancreatic cancer cells nearby.

We have added these results into our revised manuscript as fig.S7.

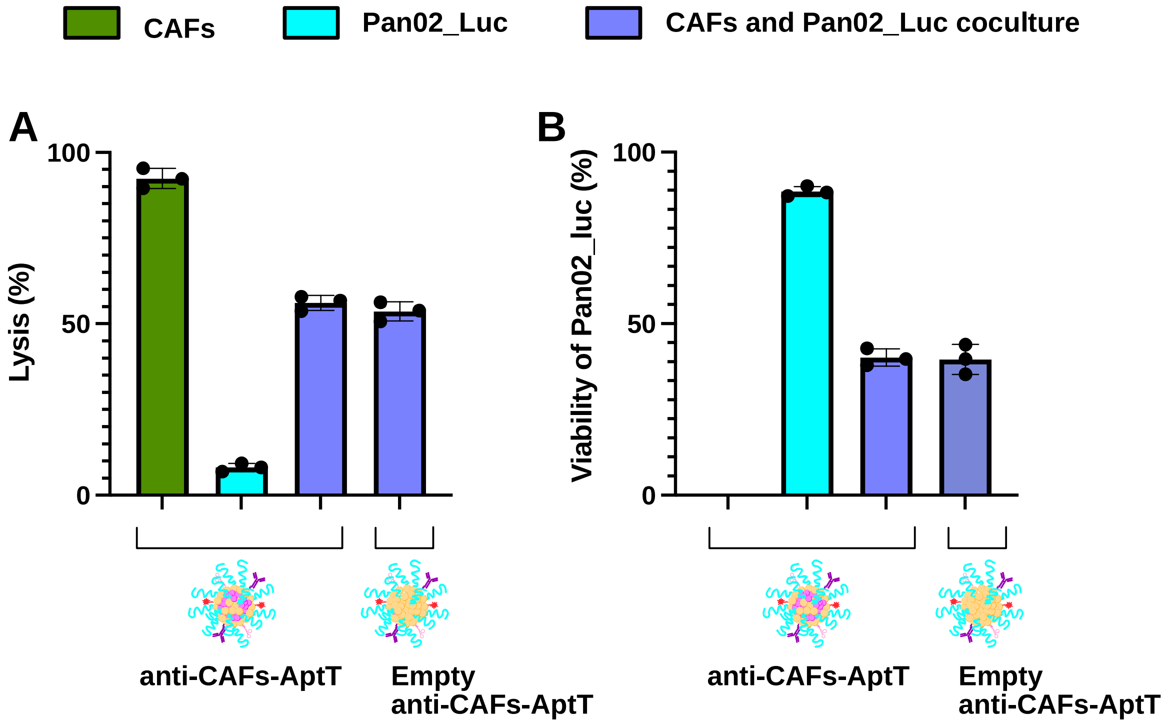

Fig#3-5. Coculture experiment to study T cell-mediated cytotoxicity. $CD8^+$ T cells as effector cells at an effector-to-target ("target" means Pan02_Luc and CAFs together, and Pan02_Luc/CAFs = 2/1) ratio of 3:1. (A), Cell lysis assay. (B), Viability assay of Pan02_Luc cells *via* the method of bioluminescence. Data points are plotted as n = 3 independent experiments.

(4) The proposed DMS system is bispecific. The effect of AG17724 is only checked in cancer cells and CAFs. With the current approach is AG17724 also delivered to T cells. What effect is AG17724 having on T cells? Is PIN1 activity affected in the T cells, and in that case, a relevant question is if this is affecting T cell function?

REPONSE: The aptamer recognizing CD8+T lymphocyte we use in our system was discovered by Nataly Kacherovsky et al. [Nature Biomedical Engineering volume 3, pages783–795 (2019)] and used for the traceless isolation of pure CD8+ T cells at low cost and high yield. Our bispecific DMS system AntiCAFs-DMS-AptT thus is designed to bind T cells but not get internalized by them. We now have added corresponding experimental result to prove this. We incubated T cells with antiCAFs-DMS-AptT-Cy5 for different time point and measured the Cy5 signal from T cells before and after DNase I treatment (DNase I treatment can degrade DNA aptamers bridging antiCAFs-DMS-AptT-Cy5 to T cells). It (**Fig#3-6)** shows that DNase I can almost completely decrease Cy5 signal from high level to the level of PBS treatment (control), indicating that antiCAFs-DMS-AptT mostly stays on cell surface of T cells rather than in the cells. We have added these results into our revised manuscript as fig.S8.

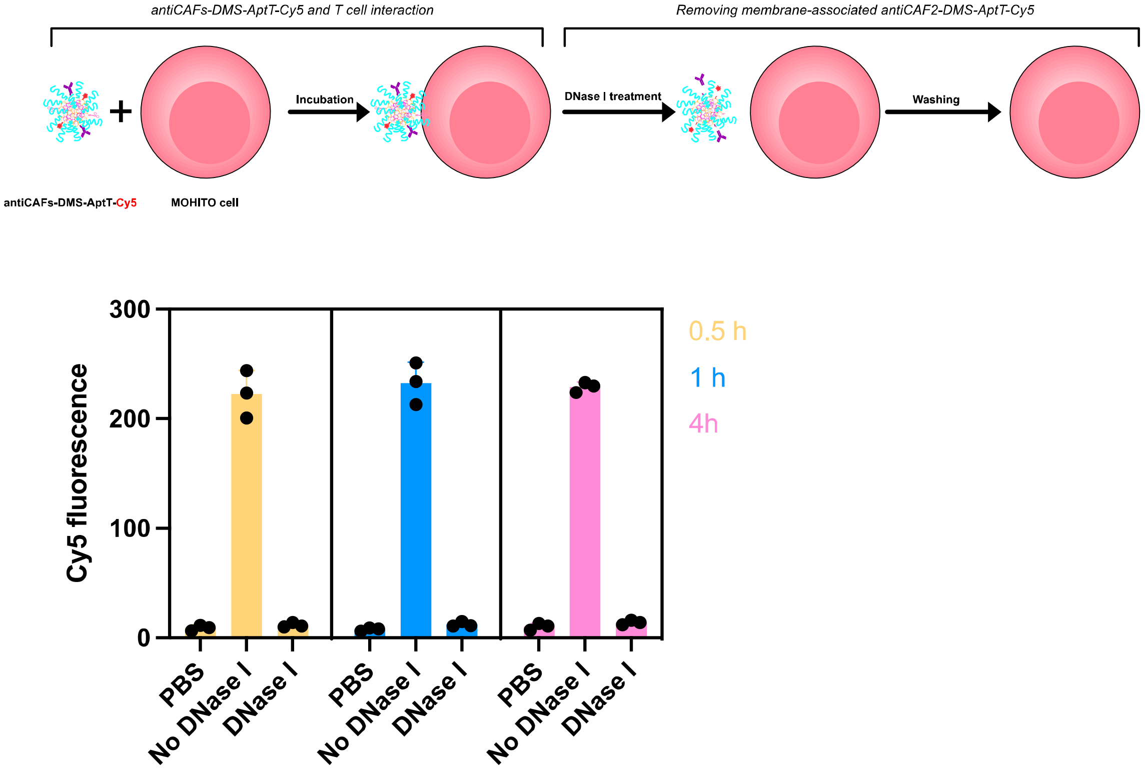

Fig#3-6. DNase I treatment workflow (Top chart) to study the association of antiCAFs-DMS-AptT-Cy5 with T cells. After different time points, Cy5 signals were detected and plotted. 3 independent experiments were carried out.

We also investigated viability of $CD8^+$ T cells (isolated from mouse spleens) after incubation with AG17724, DMS, antiCAFs-DMS or antiCAFs-DMS-AptT for 48 hours. It shows that, after being encapsulated into DMS, the toxicity of AG17724 to T cells

decreased (**Fig#3-7A**). We can attribute this to the very low uptake of our DMS systems by T cells.

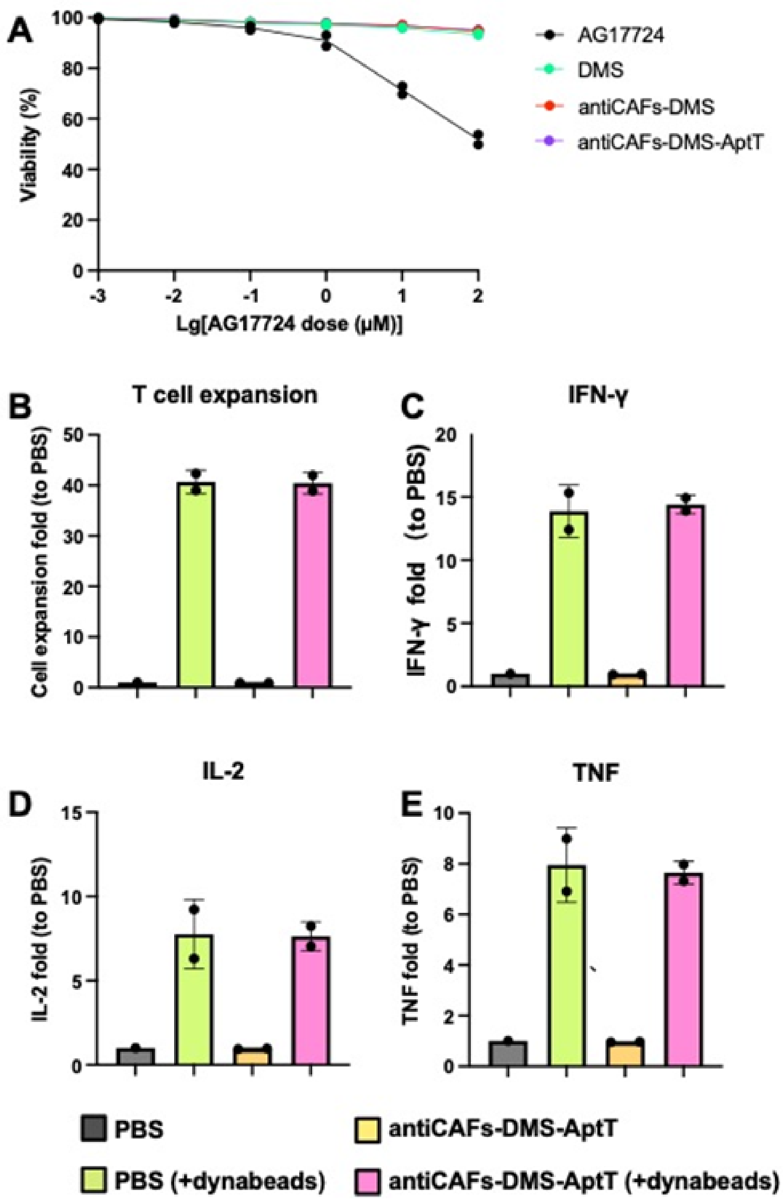

Fig#3-7. CD8⁺ T cell viability and function assays. **(A)**, Cytotoxicity on CD8⁺ T cells. **(B)** T cell expansion, **(C)** IFN-γ, **(D)** IL-2 and **(E)** TNF measurements after firstly treating the cells with/without antiCAFs-DMS-AptT for 48 hours then with/without antiCD3&antiCD28 dynabeads for 6 days. Data points are plotted as n = 2 independent experiments, with each experiment having n = 1 independent samples.

We further tested CD8⁺ T cell functions after treating them with 0.5-μM AG17724 corresponding antiCAFs-DMS-AptT, *via* measuring T cell expansion, IFN-γ, IL-2 and TNF. Our results (**Fig#3-7B, 7C, 7D, 7E**) show that antiCAFs-DMS-AptT does not

affect functions of T cells on these four aspects. We have added these results into our revised manuscript as fig.S9.

(5) The consequences of PIN1 inhibition on CAFs still needs to be addressed in further detail. In recent years it has been shown that CAFs can be further subdivided in different subtypes. The CAFs studied here are SMA positive, indicating that they have a myofibroblastic CAF phenotype. Does PIN1 inhibition lead to a switch in phenotype to a more inflammatory phenotype? Or do the CAFs become quiescent? A quite direct way of answering these questions would be to sort out the CAFs after treatment and perform bulk RNA-seq and to compare the transcriptome profiles between saline and anti-CAFs-DMS treated CAFs.

REPONSE: Thanks for your insightful suggestion. To answer this, we firstly performed lipid droplet accumulation assay to see if PIN1 inhibition *via* our method could transform myofibroblastic CAFs to quiescent phenotype. After incubating α-SMA positive CAFs isolated from tumor with 0.5-μM AG17724 or antiCAFs-DMS-AptT for 24 hours, it shows that antiCAFs-DMS-AptT could significantly increase the number of droplets in CAFs (**Fig#3-8A, 8B**). This means that efficient PIN1 inhibition can cause the metabolic deficiency of CAFs, leading them to be quiescent. Besides, we conducted cytokine assay to analyze cytokine productions of CAFs. Our results show that PIN1 inhibition caused by antiCAFs-DMS-AptT inhibited CAFs' ability to secrete a wide range of cytokines, whereas cells treated with AG17724 had similar profiles as control (**Fig#3-8C**). High expressions of cytokines, including IL-6 (**[**J. Exp. Med. 214, 579–596. | Cancer Discov. 9, 1102–1123.**]**) and IL-8 (**[**Cancer Discov. 9, 1102–1123. | https://doi.org/10.3389/fcell.2021.655152**]**), have been regarded as indicators of inflammatory CAFs. We see their decreased expressions after antiCAFs-DMS-AptT treatment. This tells us that our method doesn't really induce myofibroblastic CAFs to be inflammatory CAFs.

We agree with that RNA-seq can give us more insightful details about this. However, we have to say that, at current time, we lack corresponding experiences on RNA-seq. Importantly, our new results clearly tell us that CAFs tends to be quiescent by PIN1 inhibition, which also well matches the work of Kazuhiro Koikawa et al. [Cell. 2021 Sep 2;184(18):4753-4771.e27.] even though we use different PIN1 inhibitor. We have added these results to our revised manuscript.

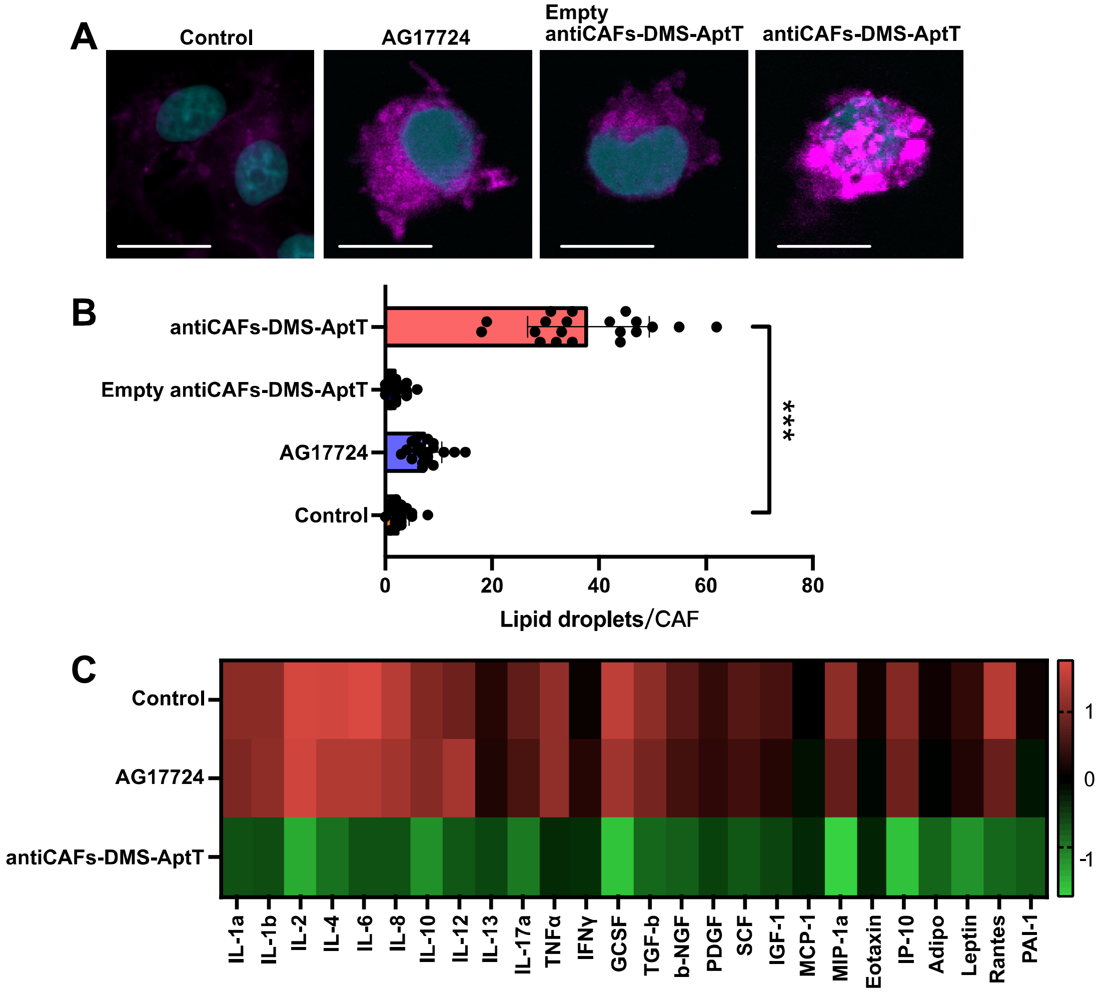

Fig#3-8. PIN1 inhibition by antiCAFs-DMS-AptT causes quiescent CAFs and inhibits a wide range of cytokines. (A-B), lipid droplet accumulation assay on α-SMA positive CAFs isolated from tumor *via* incubation with saline(control), 0.5-μM AG17724 or antiCAFs-DMS-AptT for 24 hours. Scale bars, 50 μm. Error bars, mean ± SD; ***$p < 0.001$ by one-way ANOVA. (C), cytokine production using cytokine array.

(6) In fig 5 G-H the infiltrating T cells are studied. Further profiling of these infiltrating T cells would be easy to. Are these T cells active? Are they degranulated? Exhausted? Since the T cells are identified with flowcytometry, adding an additional flow-based T cell functionality assay would be very informative.

REPONSE: We then analyzed if these tumor infiltrating $CD3^+$&$CD8^+$ T cells are exhausted *via* profiling their expressions of PD-1, TIM-3 and LAG-3 over 3 days of activation with Dynabeads CD3/CD28. Pre-exhausted CD3+& CD8+ T cells [isolated from mouse spleens, stimulated with Dynabeads CD3/CD28 (1:1 bead-to-

cell ratio) and re-stimulated every 2-3 days (5 stimulations total) prior to seeding.] were used as the control. From the result (**Fig#3-9**), we can see that CD3$^+$&CD8$^+$ T cells isolated from the tumor expressed low levels of exhaustion-related receptors. When we stimulated these cells with different concentrations Dynabeads CD3/CD28 for 3 days, we can see the increasing expression of these receptors, indicating the exhaustion progression of these cells. These data can tell us that these infiltrating T cells in tumor are not exhausted, or at least not fully exhausted.

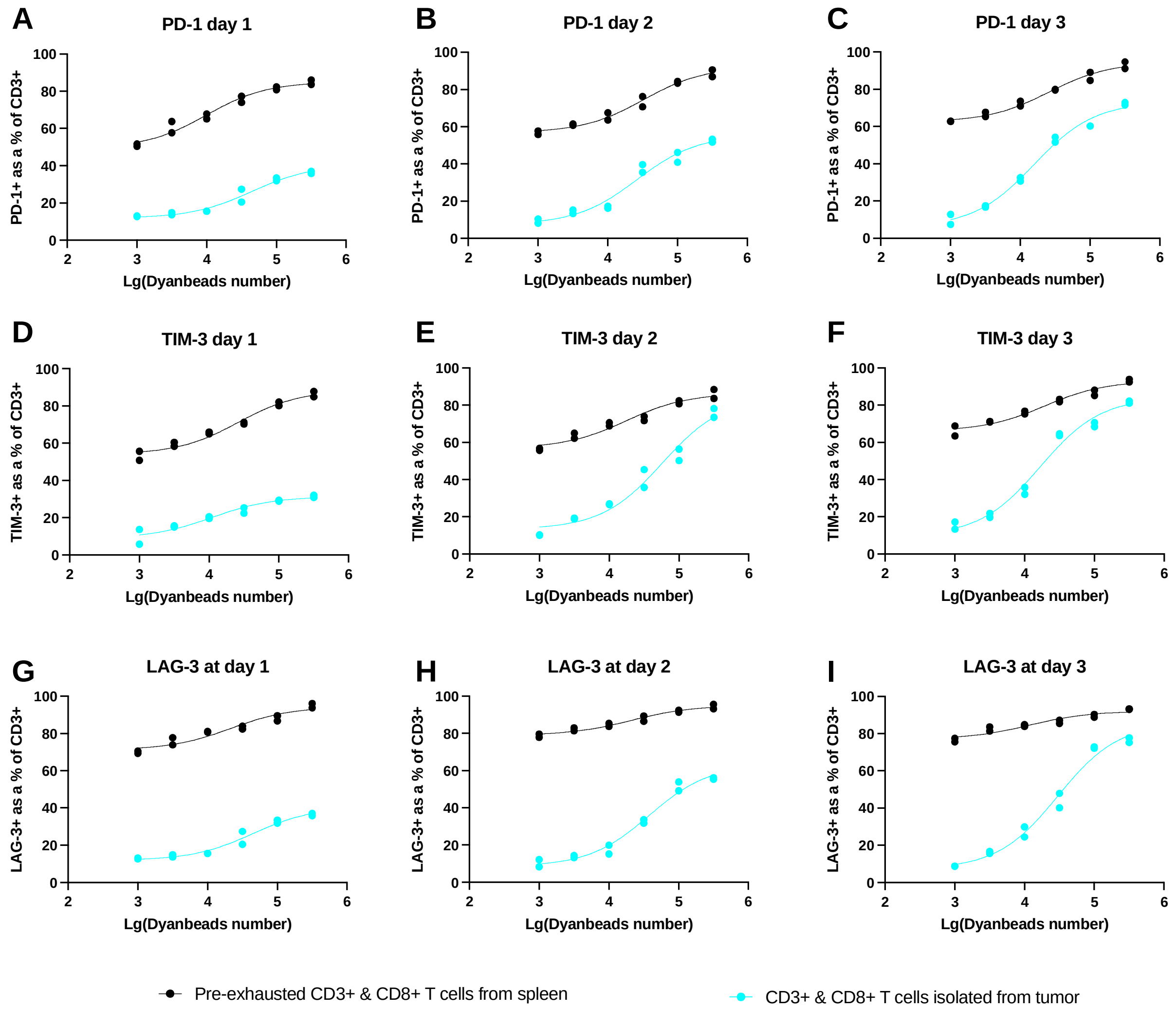

Fig#3-9. T cell exhaustion assay *via* measuring the expressions of PD-1(A-C), TIM-3 (D-F) and LAG-3 (G-I). At the 25th day of antiCAFs-DMS-AptT treatment, CD3+ & CD8+ T cells were isolated from tumor. These cells or pre-exhausted CD3+ & CD8+ T cells were seeded at 200K/well. Exhausted T cells had been repeatedly stimulated (5 times total, every 2-3 days) with Dynabeads CD3/CD28 (1:1 bead-to-cell ratio). In-well, activation was induced with Dynabeads CD3/CD28. Every 24h, 10uL samples were analysed using the iQue® Mouse T Cell Kit. Data points are plotted as n = 2 mice.

(7) Since the approach is bispecific, it is important to disentangle the effects of each component one by one, and then in combination. How much does the AptT component (and the fact that more CD8+ cells infiltrate the tumor) contribute to in vivo effect? What is PIN1 inhibition add? By adding experiments with unloaded DMS (without AG17724) this could be investigated.

REPONSE: Thanks for your insightful suggestion. To answer this, we conducted the animal study to evaluate *in vivo* therapeutic effect of unloaded DMS systems (without AG17724). On murine orthotopic model of pancreatic cancer, antiCAFs-DMS-AptT can't inhibit or slow down the progression of cancer (**Fig#3-10**). This means that, without Pin1 inhibition, empty antiCAFs-DMS-AptT (without AG17724 encapsulation) can't bridge T cells into the pancreatic tumor. This could be attributed to the inherent tumor heterogeneity and highly desmoplastic and immunosuppressive tumor microenvironment (TME) of pancreatic cancer, which limits T cell infiltration.

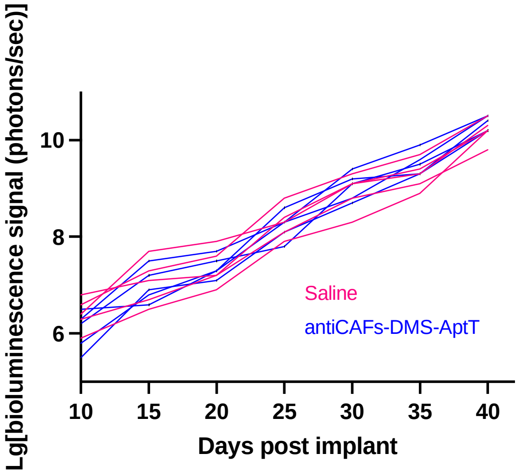

Fig#3-10. Treatment effects of empty antiCAFs-DMS-AptT (no AG17724 encapsulation) on orthotopic pancreatic cancer model. Tumor development of each mouse, quantified by bioluminescence signal.

Minor concerns:

(1) Efficient cellular uptake was demonstrated by flow cytometry-based analysis in CAFs compared to pancreatic cancer cells (Fig 2). In addition, a significant reduction of CAFs (Fig 5) together with antitumor efficacy (Fig 6) was achieved after 18 days of treatment in subcutaneous and orthotopic pancreatic ductal adenocarcinoma (PDAC) mice models. Remarkably, at day 42 a significant reduction in tumor volume was observed using the anti-CAF-

DMS-Apt system to deliver AG17724 without causing significant effects on body weight. Altogether, results suggest that the antibodies and aptamers conjugated to DMS might bind to targeted proteins with high affinity and specificity *in vivo*; nonetheless Fig 4A shows that there is a major fraction of antiCAF-DMS-Apt that accumulates in liver. Little is discussed about the repercussions that this observation might have in terms of toxicity, immunogenicity, long-term in vivo degradation, pharmacokinetics, or biocompatibility. Further safety and efficacy studies will be needed to discard toxicity of the DMS system itself. The DMS system showed high drug encapsulation and stability for one week at room temperature. Nonetheless, it is plausible that a certain fraction of the drug might be released in a non-controlled manner due to issues related to the stability of DDS *in vivo*. Therefore, the stability of anti-CAFs-DMS-Apt systems needs to be tested *in vivo*; as well as potential effects on non-targeted cells over time.

REPONSE: Yes, our biodistribution results of antiCAF-DMS-Apt in Figure 4A shows that a bigger fraction of antiCAF-DMS-Apt would accumulate in liver than in tumor. Firstly, we would say it is very common that the *in vivo* therapeutic efficacy of nanosized drugs is often significantly limited by the reticuloendothelial system (RES) barrier, with a major fraction of the intravenously administered dose distributed in the organs including liver. Overcoming the RES thus has long been a vital challenge to the field. Our main goal of this project is not to overcome this challenge.

Secondly, here the term of "targeting drug delivery" is a comparative concept: being compared with free AG17724 compound, antiCAFs-DMS and antiCAFs-DMS-Apt can change its biodistribution *via* the enhanced permeability and retention (EPR) effect **[**Nature Reviews Cancer volume 17, pages20–37 (2017).**]** and antibody-mediated targeting effect, increasing AG17724 accumulation in tumor. One very important index would be the tumor-to-liver ratio for DMS, antiCAFs-DMS and antiCAFs-DMS-AptT. Based on our imaging results in Figure 4C, we can calculate this ratio and plot it. It shows that, being compared to DMS, the accumulation ratio of tumor to liver (T/Li) of antiCAFs-DMS or antiCAFs-DMS-AptT can be improved by around 3 times. This is already a very big improvement. T/Li of antiCAFs-DMS or antiCAFs-DMS-AptT is around 0.9 (**Fig#3-11**), which already reflects on a very good tumor-targeting efficiency **[**Nature Nanotechnology volume 9, pages907–912 (2014)**]**.

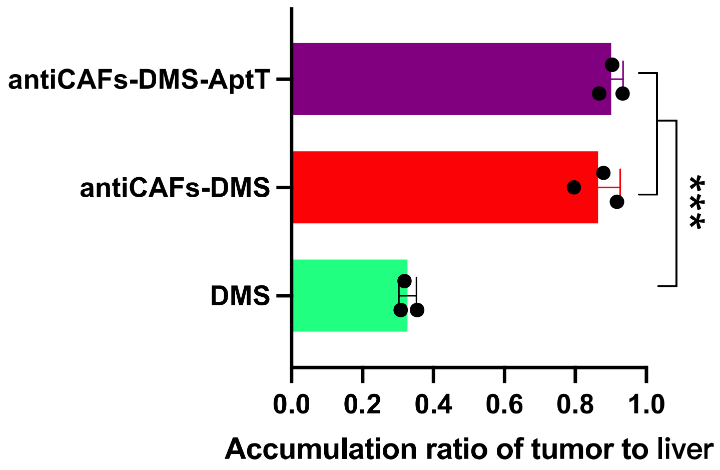

Fig#3-11. Tumor-to-liver ratio for DMS, antiCAFs-DMS and antiCAFs-DMS-AptT at 4 hours post-injection. antiCAFs-DMS and antiCAFs-DMS-AptT showed improved tumor selectivity, compared with DMS (n = 3, mean ± s.d., ***P < 0.001).

It is true that *in vivo* the DSM system will not perform an exclusive drug release only to CAFs of tumors. As we have learned, it is very difficult to directly test the stability of drug delivery systems in *vivo*. We currently can't come up with a proper method to directly assess stability of our systems *in vivo*. Since it showed that a major distribution of antiCAFs-DMS-AptT in liver, we now measured 6 indicators {GPT [glutamic-pyruvate transaminase (alanine aminotransferase)], GOT1/aspartate transaminase [glutamic-oxaloacetic transaminase 1, soluble], total protein [TP], albumin [ALB], total bilirubin [TBIL] and ALPI/alkaline phosphatase} of hepatic function at the 40th day of our treatment schedule (Figure 4D). Even though the values of these indicators were fluctuated by free AG17724 or antiCAFs-DMS-AptT treatment, they were within the normal range (**Fig#3-12**). This indicates that neither free AG17724 nor antiCAFs-DMS-AptT elicited significant liver toxicity, representing that antiCAFs-DMS-AptT is biocompatible (at least the dose of 10 mg/kg). We have added these new results into our revised manuscript.

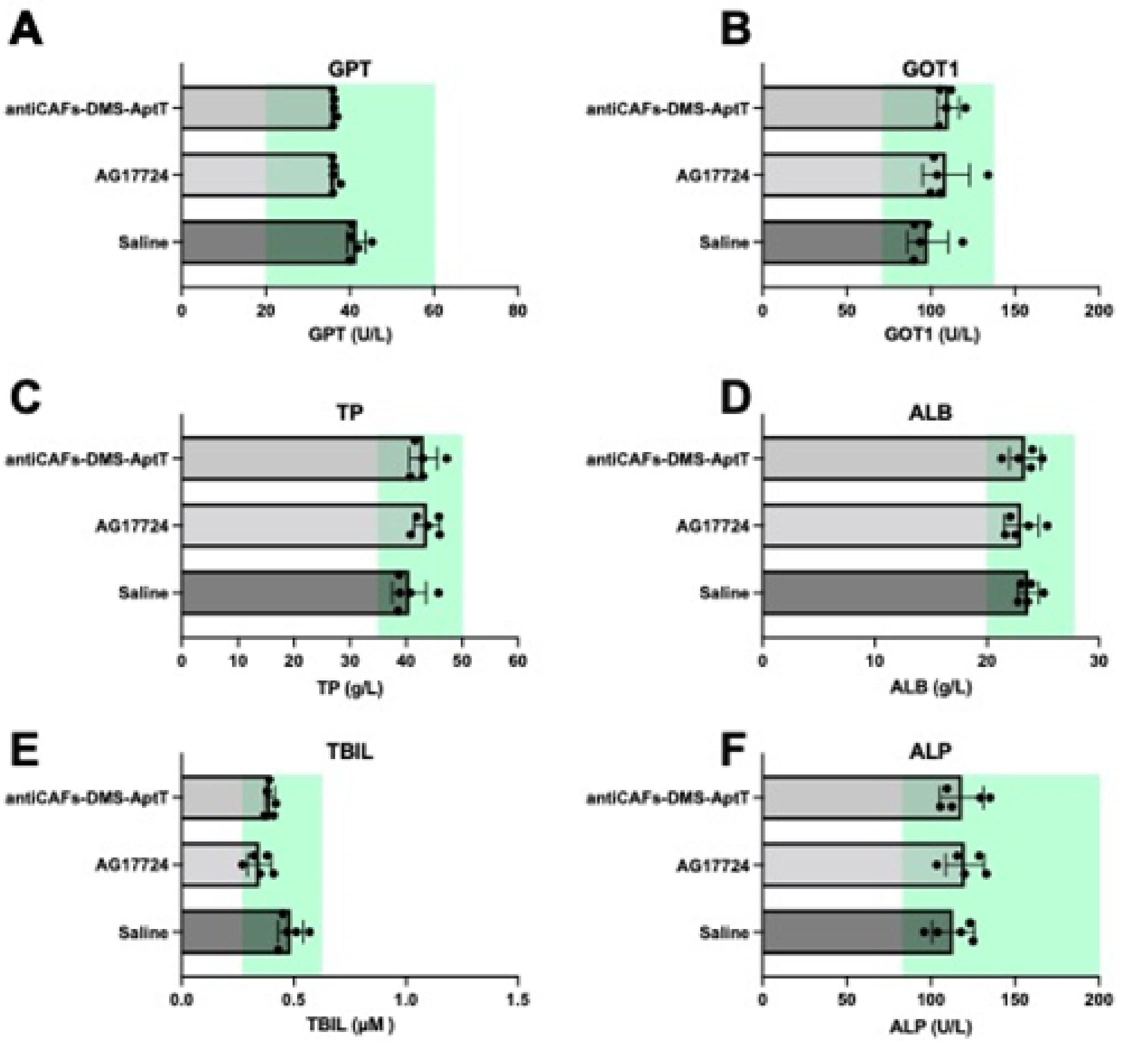

Fig#3-12. Biochemistry results from mice (n=5) treated with saline, AG17724 or antiCAFs-DMS-AptT. (A-F) These results show mean and standard deviation of the biochemistry index as indicated at the 40th day of our treatment schedule. Green areas show the normal ranges.

(2) The need and the function of the DNA barcodes needs to be explained more in detail.

REPONSE: We have added corresponding references and discussions upon the function of DNA barcodes in our revised manuscript.

(3) Grammar and proof-reading will be needed throughout the manuscript (i.e. lines 76, 88, 106, 130, 169, 246-49, 253-256, 290, 321, 332, 336-338, 354-355). Some missing references were also found (i.e. lines 208 and 218). Authors need to specify the number of technical and biological replicates in each experiment.

REPONSE: Thanks. We have carefully corrected these errors in our revised manuscript. Besides, we have specified the number of technical and biological replicates in the caption of each figure.

Taken it altogether, the authors conclude that bispecific aptamer-functionalized targeted DDS anti-CAFs-DMS-Apt system encapsulating AG17724 have shown its efficacy and potency to eradicate subcutaneous and orthotopic pancreatic ductal adenocarcinoma (PDAC). This conclusion might be considered a bit premature and overstated at this point since effective PIN1 inhibition in CAFs is not demonstrated yet, and issues regarding safety and efficacy in vivo need to be further addressed. Having said that, the anti-CAFs-DMS-Apt system is a promising therapeutic approach which offers the possibility to have a better insight into cell-level antitumor targeting therapy, as well as to better understand the biological functions of PIN1 in different cellular contexts including specific CAF subpopulations which contribute to the TME and are involved in the tumor progression.

REVIEWERS' COMMENTS

Reviewer #1 (Remarks to the Author):

Liu et al performed a lot of additional experiments to address the reviewer's concerns, and, thereby significantly improved the manuscript as well. The bispecific anti-CAFs-DMS-AptT system is very impressive, but there are still some unresolved issues. For instance, how anti-CAFs-DMS-AptT works in vivo, especially whether the CD8+ T-cells targeted by the anti-CAFs-DMS-AptT indeed exert a cytotoxic effect on cancer cells is unclear. In addition, whether the observed immune response is due to tumor antigen specific killing or whether a more non-specific cytokine based tumor cell killing mechanism is at play is uncertain. If the authors could address the concerns, the study would be more impressive.

1) The authors explained how anti-CAFs-DMS-AptT works in vivo depending on the treatment stages, as follows.
1. In the beginning, anti-CAFs-DMS-AptT might not be able to bring CD8+ T lymphocytes into the pancreatic tissues.
2. During the next treatment stage, anti-CAFs-DMS-AptT then can redirect CD8+ T lymphocytes into pancreatic tumor tissues.
However, they did not provide any convincing evidence. If the authors could investigate the tumor infiltrating CD8+ T-cells in TME in each stage in vivo using flow cytometry or IHC, the hypothesis would be convincing.

2) In Figure S7A, and S7B, cytotoxic assays in co-culture with pancreatic cancer would be important to prove whether the T-cells indeed exert a cytotoxic effect on cancer cells. The authors performed the assay using Pan02 and CD8+ T-cells (MOHITO). Could the CD8+ T-cell recognize the Pan02 cells? If so, do these two cells have the same background? Is the cytotoxic effect on cancer cells due to tumor antigen-specific immune killing or a more non-specific cytokine based cancer cell killing?

3) In Fig S9A, the authors investigated the toxicity of AG17724 to T cells in Fig#1-3A. However, it is hard to see the line and dots of anti-CAFs-DMS-AptT which should be purple. In addition, AG17724 has 2 points on each 0, 1 and 2 uM. Please update the graph.

4) In Fig S9B, the authors should explain how to investigate the T cell expansion in the Method section.

5) Again, to prove the immunosuppressive TME, the authors should investigate the immunosuppressive cells such as Tregs, MDSCs, Macrophages, not only CD8+ T-cells. Flow cytometry assay would be informative.

6) Reference #23 and #41 are the same paper.

Reviewer #2 (Remarks to the Author):

The authors addressed all of my concerns, adding controls and data that are convincing. Good job!

Reviewer #3 (Remarks to the Author):

Congratulate the authors for such a complete revised version of the manuscript. The authors have now addressed major issues in the original version adding value and relevance to their study. They have demonstrated target engagement and on-target effects through PPIase activity assays, rescue experiments in Pin1 knock-down models and downstream effects (Fig 1-1, 3-1 to 3-3). In this regard,

results showed clear downregulation of genes regulated by Pin1 after antiCAFs-DMS-AptT treatment. Minor concerns would be regarding the effects at the protein level though, so it might be good to quantify the increased in phospho-CDK1 Y15 after the antiCAFs-DMS treatment and normalized it towards the total protein.

They further demonstrated reduction of the ECM matrix after antiCAFs-DMS-Apt treatment and infiltration of active and cytotoxic CD3+ and CD8+ T cells using Collagen and Granzyme B assays in (Fig 1-2 to 1-6); as well as reduction of the tumor cell viability in both the 3D cell model (Fig 4-3) and the coculture models of CAFs and tumor cells (Fig 3-5). In addition, they show further data supporting the antiCAFs-DMS-AptT bispecific function by FACS analysis demonstrating increased intra-tumoral CD8+ T cells in vivo (Fig 2-4).

Despite more exhaustive analysis of CAFs would be needed using specific markers, the authors have attempted to address the effect on different CAF subtypes by isolating myCAFs from the tumor (SMA+ cells) and conducting cytokine arrays (Fig 3-8); which altogether suggested that CAFs go into a more quiescent phenotype with less secreted cytokines after PIN1 inhibition. There is potential for more detailed analyses here, but this can be explored in future publications.

They have also addressed questions regarding efficacy and safety of this model proving no major effects in liver cells and discarding toxicity of the DMS system itself (Fig 3-12).

In addition, and despite minor issues couldn´t be addressed due to limitations of the anti-CAFs-DMS-Apt system itself related its stability in vivo, authors have discussed and provided reasonable explanations to important question such as the CD8+ T cell infiltration and Pin1 function in CD8+ T lymphocytes, as well as its biocompatibility in vivo.

Responses to the remaining points from reviewer #1

Reviewer's Comments:

Reviewer #1 (Remarks to the Author)

Liu et al performed a lot of additional experiments to address the reviewer's concerns, and, thereby significantly improved the manuscript as well. The bispecific anti-CAFs-DMS-AptT system is very impressive, but there are still some unresolved issues. For instance, how anti-CAFs-DMS-AptT works in vivo, especially whether the CD8+ T-cells targeted by the anti-CAFs-DMS-AptT indeed exert a cytotoxic effect on cancer cells is unclear. In addition, whether the observed immune response is due to tumor antigen specific killing or whether a more non-specific cytokine based tumor cell killing mechanism is at play is uncertain. If the authors could address the concerns, the study would be more impressive.

1) The authors explained how anti-CAFs-DMS-AptT works in vivo depending on the treatment stages, as follows.
1. In the beginning, anti-CAFs-DMS-AptT might not be able to bring CD8+ T lymphocytes into the pancreatic tissues.
2. During the next treatment stage, anti-CAFs-DMS-AptT then can redirect CD8+ T lymphocytes into pancreatic tumor tissues.
However, they did not provide any convincing evidence. If the authors could investigate the tumor infiltrating CD8+ T-cells in TME in each stage in vivo using flow cytometry or IHC, the hypothesis would be convincing.
Response: Thanks. This is our explanation (or discussion) to your previous question regarding "how antiCAFs-DMS-AptT deliver CD8+T-cell into the tumor microenvironment in vivo?". Our *in vivo* anti-PDAC therapeutic results (Figure 6) and intra-tumoral T cell profiling data (Figure 5G) support that antiCAFs-DMS-AptT indeed efficiently direct CD8+ T cells to tumor and result in PDAC eradication. It's true that the dense composition of PDAC limits the infiltration of immune cells. Based our results and this characteristic of PDAC together, we thus explained/discussed the potential mechanism with "dependency of the treatment stages". We think it is a reasonable explanation/discussion. With our current data, we think the core novelty and completeness of our work is about the nano-system itself and its function to specifically inhibit PIN in CAFs and mediate T cell cytotoxicity to PDAC. We would more specifically investigate this [if it is true that how anti-CAFs-DMS-AptT works *in vivo* depends on the treatment stages?] and show more evidence in our future work.

2) In Figure S7A, and S7B, cytotoxic assays in co-culture with pancreatic cancer would be important to prove whether the T-cells indeed exert a cytotoxic effect on cancer cells. The authors performed the assay using Pan02 and CD8+ T-cells (MOHITO). Could the CD8+ T-cell recognize the Pan02 cells? If so, do these two cells have the same background? Is the cytotoxic effect on cancer cells due to tumor antigen-specific immune killing or a more non-specific cytokine based cancer cell killing?
Response: Thanks. In term of mediating the T cell killing, our nano-system is designed to function similarly as bispecific antibodies like bispecific T cell engagers (BiTEs). As proven in many previous publications, BiTEs works *via* bridging cytotoxic T cell with targeting cell without the need of specific recognition of CD8+ T cell to cancer cells [Nature Reviews Clinical Oncology volume 17, pages418–434 (2020)]. This is another concept which differs from the tumor antigen recognition by TCR.

3) In Fig S9A, the authors investigated the toxicity of AG17724 to T cells in Fig#1-3A. However, it is hard to see the line and dots of anti-CAFs-DMS-AptT which should be purple. In addition, AG17724 has 2 points on each 0, 1 and 2 uM. Please update the graph.
Response: Thanks. Because the lines and dots of DMS, antiCAFs-DMS and antiCAFs-DMS-AptT are quite overlapping with each other, they don't show separately.

4) In Fig S9B, the authors should explain how to investigate the T cell expansion in the Method section.
Response: After stain the cells with trypan blue, we count the live and dead T cells by using automated cell counter (Countess™ 3, Invitrogen). We have added this to our Methods section.

5) Again, to prove the immunosuppressive TME, the authors should investigate the immunosuppressive cells such as Tregs, MDSCs, Macrophages, not only CD8+ T-cells. Flow cytometry assay would be

informative.
Response: Thanks. In this work, since our bispecific nano-system is designed to bring CD8+ T cells to tumor, we thus mainly focus on the activity of CD8+ T cells in TME. As we have shown, CD8+ T cells were successfully brought to tumors by our bispecific nano-system, also these infiltrating CD8+ T cells in tumors are active rather than suppressed or exhausted. We agree that investigating populations of all immune cells in the tumor is more informative, and we will profile them in our future work. But we think that the more important thing is the final outcome---the tumors were eradicated by our bispecific nano-system, and it is directly linked to the activity of CD8+ T cells in tumors. We have now also analyzed the cell populations of orthotopic PDAC. It shows that antiCAFs-DMS-AptT treatment resulted in not only CAFs depletion (Fig.7E) but also the increase of CD8+ T cells (Fig.7F, 7G) in tumors. We think this would be another set of strong evidence to support our current discovery.

6) Reference #23 and #41 are the same paper.
Response: Thanks for pointing this mistake out. We have carefully checked and managed our references now.

Reviewer #2 (Remarks to the Author)

The authors addressed all of my concerns, adding controls and data that are convincing. Good job!

Reviewer #3 (Remarks to the Author)

Congratulate the authors for such a complete revised version of the manuscript. The authors have now addressed major issues in the original version adding value and relevance to their study. They have demonstrated target engagement and on-target effects through PPIase activity assays, rescue experiments in Pin1 knock-down models and downstream effects (Fig 1-1, 3-1 to 3-3). In this regard, results showed clear downregulation of genes regulated by Pin1 after antiCAFs-DMS-AptT treatment. Minor concerns would be regarding the effects at the protein level though, so it might be good to quantify the increased in phospho-CDK1 Y15 after the antiCAFs-DMS treatment and normalized it towards the total protein.

They further demonstrated reduction of the ECM matrix after antiCAFs-DMS-Apt treatment and infiltration of active and cytotoxic CD3+ and CD8+ T cells using Collagen and Granzyme B assays in (Fig 1-2 to 1-6); as well as reduction of the tumor cell viability in both the 3D cell model (Fig 4-3) and the coculture models of CAFs and tumor cells (Fig 3-5). In addition, they show further data supporting the antiCAFs-DMS-AptT bispecific function by FACS analysis demonstrating increased intra-tumoral CD8+ T cells in vivo (Fig 2-4).

Despite more exhaustive analysis of CAFs would be needed using specific markers, the authors have attempted to address the effect on different CAF subtypes by isolating myCAFs from the tumor (SMA+ cells) and conducting cytokine arrays (Fig 3-8); which altogether suggested that CAFs go into a more quiescent phenotype with less secreted cytokines after PIN1 inhibition. There is potential for more detailed analyses here, but this can be explored in future publications.

They have also addressed questions regarding efficacy and safety of this model proving no major effects in liver cells and discarding toxicity of the DMS system itself (Fig 3-12).

In addition, and despite minor issues couldn´t be addressed due to limitations of the anti-CAFs-DMS-Apt system itself related its stability in vivo, authors have discussed and provided reasonable explanations to important question such as the CD8+ T cell infiltration and Pin1 function in CD8+ T lymphocytes, as well as its biocompatibility in vivo.
